# Translates of rational points along expanding closed horocycles on the modular surface

**DOI:** 10.1007/s00208-021-02267-7

**Published:** 2021-09-20

**Authors:** Claire Burrin, Uri Shapira, Shucheng Yu

**Affiliations:** 1 Department of Mathematics, Rutgers University, 110 Frelinghuysen Rd, Piscataway, NJ 08854 USA; 2 Present Address: Department Mathematik, ETH Zürich, 8092 Zurich, Switzerland; 3 Department of Mathematics, Technion, Haifa, Israel; 4 Present Address: Department of Mathematics, Uppsala University, Box 480, 75106 Uppsala, Sweden

## Abstract

We study the limiting distribution of the rational points under a horizontal translation along a sequence of expanding closed horocycles on the modular surface. Using spectral methods we confirm equidistribution of these sample points for any translate when the sequence of horocycles expands within a certain polynomial range. We show that the equidistribution fails for generic translates and a slightly faster expanding rate. We also prove both equidistribution and non-equidistribution results by obtaining explicit limiting measures while allowing the sequence of horocycles to expand arbitrarily fast. Similar results are also obtained for translates of primitive rational points.

## Introduction

Let $\{S_n\}_{n\in {\mathbb {N}}}$ be a sequence of “nice” subsets that become equidistributed in their ambient space. Given a sequence of discrete subsets $\{R_n\}_{n\in {\mathbb {N}}}$ with $R_n\subset S_n$, an interesting question is to study to what extent does the distribution behavior of $\{R_n\}_{n\in {\mathbb {N}}}$ mimic that of $\{S_n\}_{n\in {\mathbb {N}}}$. One naturally expects that when the size of $R_n$ is relatively large, it is more likely that $\{R_n\}_{n\in {\mathbb {N}}}$ inherits some distribution property from $\{S_n\}_{n\in {\mathbb {N}}}$; on the other hand if $R_n$ lies on $S_n$ sparsely, then it is more likely that points in $\{R_n\}_{n\in {\mathbb {N}}}$ become decorrelated and distribute like random points on the ambient space.

In the setting of unipotent dynamics, the most typical example of a sequence $\{S_n\}_{n\in {\mathbb {N}}}$ is a sequence of *expanding closed horocycles* on a non-compact finite-area hyperbolic surface ${\mathcal {M}}$. More precisely, we can realize ${\mathcal {M}}$ as a quotient $\Gamma \backslash {\mathbb {H}}$ where $\Gamma$ is a co-finite Fuchsian subgroup and ${\mathbb {H}}=\{z=x+iy\in {\mathbb {C}}:y>0\}$ is the Poincaré upper half-plane, equipped with the hyperbolic metric $ds=|dz|/y$, where $dz=dx+idy$ is the complex line element. Up to conjugating by an appropriate isometry, we may assume that ${\mathcal {M}}=\Gamma \backslash {\mathbb {H}}$ has a width one cusp at infinity, that is, that the isotropy group $\Gamma _{\infty }< \Gamma$ is generated by the translation sending $z\in {\mathbb {H}}$ to $z+1$. A *closed horocycle of height* $y>0$ is a closed set of the form

$$\begin{aligned} {\mathcal {H}}_y:=\{\Gamma (x+iy): x\in {\mathbb {R}}/{\mathbb {Z}}\} \subset {\mathcal {M}}, \end{aligned}$$

and its period, i.e., its hyperbolic length, is $y^{-1}$. As ${\mathcal {H}}_y$ gets longer, that is, as $y\rightarrow 0^+$, it becomes equidistributed on ${\mathcal {M}}$ with respect to the hyperbolic area $d\mu (z)=y^{-2}dxdy$. The first effective version of this result is due to Sarnak [28] who, using spectral arguments, proved that for every $\Psi \in C_c^\infty (\Gamma \backslash {\mathbb {H}})$ and any $y>0$,

1.1 $$\begin{aligned} \int _0^1 \Psi (x+iy) dx =\frac{\int _{{\mathcal {M}}}\Psi (z)d\mu (z)}{\mu ({\mathcal {M}})} +O\left( {\mathcal {S}}(\Psi ) y^{\alpha }\right) , \end{aligned}$$

where ${\mathcal {S}}$ is some Sobolev norm, and $0<\alpha <1$ is a constant depending on the first non-trivial residual hyperbolic Laplacian eigenvalue of $\Gamma$. In the case of the modular surface ${\text {SL}}_2({\mathbb {Z}})\backslash {\mathbb {H}}$, $\alpha =\frac{1}{2}$, while Zagier [32] observed that the Riemann hypothesis is equivalent to the equidistribution rate $O_{\epsilon }\left( y^{3/4-\epsilon }\right)$.

In this setting, this problem was first investigated by Hejhal in [12] with a heuristic and numerical study of the value distribution of the sample points

1.2 $$\begin{aligned} \Gamma \left( \tfrac{x+j}{n}+iy\right) : 0\le j\le n-1 \end{aligned}$$

for some Hecke triangle groups $\Gamma ={\mathbb {G}}_q$ under the assumption that *ny* is small. Set

$$\begin{aligned} S_{y,n,\Psi }(x):=\sum _{j=0}^{n-1}\Psi \left( \tfrac{x+j}{n}+iy\right) , \end{aligned}$$

where $\Psi$ is some mean-zero step function on a fixed fundamental domain for $\Gamma \backslash {\mathbb {H}}$ (automorphically extended to ${\mathbb {H}}$). The numerics show that the value distribution of $n^{-1/2}S_{n,y,\Psi }(x)$ with respect to $x\in [0,1)$ approaches a Gaussian curve for the non-arithmetic Hecke triangle groups ${\mathbb {G}}_5$ and ${\mathbb {G}}_7$, while this phenomenon breaks down for ${\mathbb {G}}_3={\text {PSL}}_2({\mathbb {Z}})$. Hejhal gave an explanation of this difference based on the existence of Hecke operators on ${\mathbb {G}}_3$. The convergence to a Gaussian distribution for general non-arithmetic Fuchsian groups was later confirmed by Strömbergsson [30, Corollary 6.5], under the assumption that the sequence $\{y_n\}_{n\in {\mathbb {N}}}$ decays sufficiently rapidly.

Other such problems have since been investigated. Marklof and Strömbergsson [27] proved the equidistribution of generic Kronecker sequences

1.3 $$\begin{aligned} \{\Gamma (j\beta +iy_n)\in {\mathcal {M}}:1\le j\le n\} \subset {\mathcal {M}}\end{aligned}$$

along a sequence of closed horocycles expanded at a certain rate $y_n$ on $T_1{\mathcal {M}}$, the unit tangent bundle of ${\mathcal {M}}$. The equidistribution of Hecke points proved by Clozel–Ullmo [4] (see also [3, 10]) implies the equidistribution of the primitive rational points

$$\begin{aligned} \left\{ \Gamma \left( \tfrac{j}{n}+\tfrac{i}{n}\right) : 1\le j\le n-1,\ \gcd (j,n)=1\right\} \end{aligned}$$

at prime steps on the modular surface, see [10, Remark on p. 171]. More recently, the equidistribution of the above sequence along the full sequence of positive integers was proved by Einsiedler–Luethi–Shah [8] in a slightly more general setting, namely on the product of the unit tangent bundle of the modular surface and a torus. Various sparse equidistribution results have also been obtained for expanding horospheres in the space of lattices ${\text {SL}}_n({\mathbb {R}})/{\text {SL}}_n({\mathbb {Z}})$ for $n\ge 3$ [7, 9, 22, 23, 26] and in Hilbert modular surfaces [24].

For each of these equidistribution results, assumptions on the expanding rate of the sequence $\{S_n\}_{n\in {\mathbb {N}}}$ are crucial; the discrete subsets $\{R_n\}_{n\in {\mathbb {N}}}$ lying on $\{S_n\}_{n\in {\mathbb {N}}}$ can not be too sparse.

This paper emerged from an attempt to prove a result which turned out to be false. We consider the *sparse equidistribution problem* for the subset of rational points (with denominator *n*) under a horizontal translation $x\in {\mathbb {R}}/{\mathbb {Z}}$ on a horocycle ${\mathcal {H}}_{y}$ on the modular surface; we denote this subset by ${\mathcal {R}}_{n}(x,y_n)$ (cf. (1.4)). We thought that since the closed horocycles ${\mathcal {H}}_y$ equidistribute as $y\rightarrow 0^+$, if we fix a sequence $\{y_n\}_{n\in {\mathbb {N}}}$ approaching zero, then the normalized counting measures on ${\mathcal {R}}_n(x,y_n)$ (and its primitive counterpart) should equidistribute for Lebesgue almost every *x* as $n\rightarrow \infty$. See the recent paper of Bersudsky [1, Theorem 1.5] for an analogue situation where such a result is true. Note the order of quantifiers; we first fix the sequence $\{y_n\}_{n\in {\mathbb {N}}}$ and only then choose the horizontal translation *x*. It is not hard to see that if one flips the quantifiers, for any fixed horizontal translation *x*, there are sequences $\{y_n\}_{n\in {\mathbb {N}}}$ (approaching zero rapidly) such that equidistribution fails. We were very surprised to learn though, that in stark contrast to our initial expectation, equidistribution fails. The main novel result of this paper (Theorem 1.5) says that there are sequences $\{y_n\}_{n\in {\mathbb {N}}}$ approaching zero arbitrarily fast such that for almost every horizontal translation *x* the normalized counting measures ${\mathcal {R}}_n(x,y_n)$ and its primitive counterpart do not equidistribute. In fact, we show the collection of limit measures contains the uniform measure $\mu _{{\mathcal {M}}}$, the zero measure and certain singular measures. Although these should be considered as the main contribution of this paper, we also complement our analysis with answering natural questions concerning sequences $\{y_n\}_{n\in {\mathbb {N}}}$ approaching zero in a polynomial rate.

The next subsections describe more precisely the setting and results obtained.

### Context of the present paper

Let $\Gamma ={\text {SL}}_2({\mathbb {Z}})$ and let ${\mathcal {M}}=\Gamma \backslash {\mathbb {H}}$ be the modular surface. In this paper, generalizing the setting of [8], we study the equidistribution problem for the sets of rational and primitive rational points under an arbitrary horizontal translation $x\in {\mathbb {R}}/{\mathbb {Z}}$ along a given sequence of expanding closed horocycles on ${\mathcal {M}}$. The set of rational points is the obvious choice of a sparse set with identical spacings, while primitive rational points constitute the simplest pseudorandom sequence (via the linear congruential generator). For any $n\in {\mathbb {N}}$, $x\in {\mathbb {R}}/{\mathbb {Z}}$ and $y>0$ we denote by

1.4 $$\begin{aligned} {\mathcal {R}}_n(x,y):=\left\{ \Gamma \left( x+\tfrac{j}{n}+iy\right) \in {\mathcal {H}}_y:0\le j\le n-1\right\} \end{aligned}$$

and respectively

1.5 $$\begin{aligned} {\mathcal {R}}_n^{\mathrm{pr}}(x,y):=\left\{ \Gamma \left( x+\tfrac{j}{n}+iy\right) \in {\mathcal {H}}_y:j\in ({\mathbb {Z}}/n{\mathbb {Z}})^{\times }\right\} , \end{aligned}$$

the set of rational and respectively primitive rational points with denominator *n* on the closed horocycle ${\mathcal {H}}_y$ translated to the right by *x*. As usual, $({\mathbb {Z}}/n{\mathbb {Z}})^{\times }$ denotes here the multiplicative group of integers modulo *n*.

Let $\{y_n\}_{n\in {\mathbb {N}}}$ be a sequence of positive numbers such that $y_n\rightarrow 0$ as $n\rightarrow \infty$. We investigate the *limiting distribution* of the sequences of sample points $\left\{ {\mathcal {R}}_n(x,y_n)\right\} _{n\in {\mathbb {N}}}$ and $\left\{ {\mathcal {R}}^{\mathrm{pr}}_n(x,y_n)\right\} _{n\in {\mathbb {N}}}$ under various assumptions on the expanding rate of the sequence of horocycles $\{{\mathcal {H}}_{y_n}\}_{n\in {\mathbb {N}}}$, or equivalently, the decay rate of $\{y_n\}_{n\in {\mathbb {N}}}$.

This problem is naturally easier when the sequence $\{y_n\}_{n\in {\mathbb {N}}}$ decays slowly since then at each step we have relatively more sample points on the underlying horocycle. For instance, if $ny_n\rightarrow \infty$ as $n\rightarrow \infty$, the hyperbolic distance between two adjacent points in ${\mathcal {R}}_n(x,y_n)$ decays to zero as $n\rightarrow \infty$. Since the points in ${\mathcal {R}}_n(x,y_n)$ distribute evenly on ${\mathcal {H}}_{y_n}$, the distribution behavior of ${\mathcal {R}}_n(x,y_n)$ then mimics that of ${\mathcal {H}}_{y_n}$. In particular, for any $x\in {\mathbb {R}}/{\mathbb {Z}}$ the sequence $\left\{ {\mathcal {R}}_n(x,y_n)\right\} _{n\in {\mathbb {N}}}$ becomes equidistributed on ${\mathcal {M}}$ with respect to the hyperbolic area $\mu$ as $n\rightarrow \infty$, following from the equidistribution of the sequence $\{{\mathcal {H}}_{y_n}\}_{n\in {\mathbb {N}}}$.

Regarding $\left\{ {\mathcal {R}}^{\mathrm{pr}}_n(x,y_n)\right\} _{n\in {\mathbb {N}}}$, its distribution behavior is well understood when $x=0$. Indeed, it was shown by Luethi [24] that if $y_n=c/n^{\alpha }$ for some $c>0$ and some $\alpha \in (0,1)$, then ${\mathcal {R}}_n^{\mathrm{pr}}(0,y_n)$ becomes equidistributed on ${\mathcal {M}}$ with respect to $\mu$ as $n\rightarrow \infty$. Moreover, under the simple symmetry relation that for $\gcd (j,n)=1$ and $y>0$

1.6 $$\begin{aligned} \Gamma \left( \tfrac{j}{n}+iy\right) =\Gamma \left( -\tfrac{{\overline{j}}}{n}+ \tfrac{i}{n^2y}\right) , \end{aligned}$$

one can extend this equidistribution result to the range $\alpha \in (1,2)$; this improves the previous work of Demirci Akarsu [5, Theorem 2] which confirms equdistribution of $\{{\mathcal {R}}_n^{\mathrm{pr}}(0, c/n^{\alpha })\}_{n\in {\mathbb {N}}}$ for $\alpha \in (\frac{3}{2}, 2)$. Here ${\overline{j}}\in ({\mathbb {Z}}/n{\mathbb {Z}})^{\times }$ denotes the multiplicative inverse of $j\in ({\mathbb {Z}}/n{\mathbb {Z}})^{\times }$. The equidistribution for the case $\alpha =1$ was later proved by Einsiedler–Luethi–Shah [8]; Jana [16, Theorem 1] recently gave an alternative spectral proof to this equidistribution result. We also mention that both [5, Theorem 2] and [16, Theorem 1] are valid in the same setting as [8], namely, on the product of the unit tangent bundle of the modular surface and a torus. When $\alpha =2$ the equidistribution fails as the aforementioned symmetry implies that ${\mathcal {R}}^{\mathrm{pr}}_n(0,c/n^2)={\mathcal {R}}_n^{\mathrm{pr}}(0, 1/c)$ is always trapped in the closed horocycle ${\mathcal {H}}_{1/c}$. For the same reason, when $\alpha >2$ (or more generally for any sequence satisfying $n^2y_n\rightarrow 0$), one has with ${\mathcal {R}}^{\mathrm{pr}}_n(0,c/n^{\alpha })={\mathcal {R}}_n^{\mathrm{pr}}(0, n^{\alpha -2}/c)\subset {\mathcal {H}}_{n^{\alpha -2}/c}$ a full escape to the cusp of ${\mathcal {M}}$ as $n\rightarrow \infty$. It is worth noting that while the symmetry (1.6) still holds for rational translates (cf. Lemma 3.6), it breaks down for irrational translates.

### Statements of the results

We will state here the main results of this paper, and postpone the discussion of their proofs to the next subsection. Let $\mu _{{\mathcal {M}}}:=\mu ({\mathcal {M}})^{-1}\mu$ be the normalized hyperbolic area on ${\mathcal {M}}$. For any $n\in {\mathbb {N}}$, $x\in {\mathbb {R}}/{\mathbb {Z}}$ and $y>0$ let $\delta _{n,x,y}$ and $\delta _{n,x,y}^{\mathrm{pr}}$ denote the normalized probability counting measure supported on ${\mathcal {R}}_n(x,y)$ and ${\mathcal {R}}_n^{\mathrm{pr}}(x,y)$ respectively. That is, for any $\Psi \in C_c^{\infty }({\mathcal {M}})$,

$$\begin{aligned} \delta _{n,x,y}(\Psi )=\frac{1}{n}\sum _{j=0}^{n-1}\Psi \left( x+\tfrac{j}{n}+iy\right) , \end{aligned}$$

and

$$\begin{aligned} \delta _{n,x,y}^{\mathrm{pr}}(\Psi )=\frac{1}{\varphi (n)}\sum _{j\in ({\mathbb {Z}}/n{\mathbb {Z}})^{\times }}\Psi \left( x+\tfrac{j}{n}+iy\right) , \end{aligned}$$

where $\varphi$ is Euler’s totient function. Here and throughout, for any measure $\nu$ on ${\mathcal {M}}$, we set $\nu (\Psi ):=\int _{{\mathcal {M}}}\Psi (z)d\nu (z)$.

Using spectral expansion and collecting estimates on the Fourier coefficients of Hecke–Maass forms and Eisenstein series, we obtain the following effective result, which yields equidistribution when the sequence is within a certain polynomial range.

#### Theorem 1.1

Let ${\mathcal {M}}$ be the modular surface. For any $\Psi \in C_c^{\infty }({\mathcal {M}})$, for any $n\in {\mathbb {N}}$, $x\in {\mathbb {R}}/{\mathbb {Z}}$ and $y>0$ we have

$$\begin{aligned} \left| \delta _{n,x,y}(\Psi )-\mu _{{\mathcal {M}}}(\Psi )\right| \ll _{\epsilon }{\mathcal {S}}_{2,2}(\Psi )\left( y^{1/2}+ n^{-1}y^{-(1/2+\theta +\epsilon )}\right) , \end{aligned}$$

and

$$\begin{aligned} \left| \delta _{n,x,y}^{\mathrm{pr}}(\Psi )-\mu _{{\mathcal {M}}}(\Psi )\right| \ll _{\epsilon }{\mathcal {S}}_{2,2}(\Psi )\left( y^{1/2}+n^{-1+\epsilon }y^{-(1/2+\theta +\epsilon )}\right) , \end{aligned}$$

where $\theta =7/64$ is the current best known bound towards the Ramanujan conjecture (which implies $\theta =0$) and ${\mathcal {S}}_{2,2}$ is a ”$L^2$, order-2” Sobolev norm on $C_c^{\infty }({\mathcal {M}})$, see Sect. 2.1.

If $\{y_n\}_{n\in {\mathbb {N}}}$ is a sequence of positive numbers satisfying $\lim \nolimits _{n\rightarrow \infty }y_n=0$ and $y_n\gg 1/n^{\alpha }$ for some fixed $\alpha \in \left( 0, \frac{2}{1+2\theta }\right) =(0,\tfrac{64}{39})$, then Theorem 1.1 implies that for any translate $x\in {\mathbb {R}}/{\mathbb {Z}}$, both $\left\{ {\mathcal {R}}_n(x,y_n)\right\} _{n\in {\mathbb {N}}}$ and $\left\{ {\mathcal {R}}_n^{\mathrm{pr}}(x,y_n)\right\} _{n\in {\mathbb {N}}}$ become equidistributed on ${\mathcal {M}}$ with respect to $\mu _{{\mathcal {M}}}$ as $n\rightarrow \infty$. In particular, it gives an alternative – spectral – proof to the aforementioned results of Luethi [24] and Einsiedler–Luethi–Shah [8]. The upper bound $\tfrac{2}{1+2\theta }$ is the natural barrier for our spectral methods. Nevertheless, when *x* is a rational translate, a generalization of the symmetry (1.6) allows to go beyond this barrier, and to prove unconditionally the remaining range $\alpha \in [\tfrac{2}{1+2\theta },2)$, as holds in the case of $\{{\mathcal {R}}_n^{\mathrm{pr}}(0,y_n)\}_{n\in {\mathbb {N}}}$.

#### Theorem 1.2

Let $x=p/q$ be a primitive rational number, i.e. $\gcd (p,q)=1$. Let $\{y_n\}_{n\in {\mathbb {N}}}$ be a sequence of positive numbers satisfying $y_n\asymp 1/n^{\alpha }$ for some fixed $\alpha \in [\tfrac{2}{1+2\theta },2)$. Then both $\left\{ \delta _{n,x,y_n}\right\} _{n\in {\mathbb {N}}_{q}}$ and $\left\{ \delta _{n,x,y_n}^{\mathrm{pr}}\right\} _{n\in {\mathbb {N}}_{q}^{\mathrm{pr}}}$ weakly converge to $\mu _{{\mathcal {M}}}$ as *n* goes to infinity, where

$$\begin{aligned} {\mathbb {N}}_q:=\{n\in {\mathbb {N}}: \gcd (n^2, q)\mid n\}\quad \text {and}\quad {\mathbb {N}}_q^{\mathrm{pr}}:=\{n\in {\mathbb {N}}: \gcd (n,q)=1\}. \end{aligned}$$

#### Remark 1.7

If *q* is squarefree, then the condition $\gcd (n^2, q)\mid n$ is void. Thus for such *q*, Theorem 1.2 (together with Theorem 1.1) confirms the equidistribution of the sample points ${\mathcal {R}}_n(p/q, y_n)$ (with $y_n\asymp 1/n^{\alpha }$) along the full set of positive integers for any $0<\alpha <2$.

As a byproduct of our analysis, we also have the following non-equidistribution result for rational translates, giving infinitely many explicit limiting measures. Let us first fix some notation. For each $m\in {\mathbb {N}}$, let

1.8 $$\begin{aligned} {\mathbb {P}}_{m}:=\{n=m\ell \in {\mathbb {N}}: \ell \,\text {is a prime number and}\, \ell \not \mid m\}. \end{aligned}$$

For each $Y>0$, we denote by $\mu _{Y}$ the uniform probability measure supported on the closed horocycle ${\mathcal {H}}_Y$. For each $m\in {\mathbb {N}}$ and $Y>0$, we define the probability measure $\nu _{m,Y}$ on ${\mathcal {M}}$ by

1.9 $$\begin{aligned} \nu _{m,Y}:=\frac{1}{m}\sum _{d\mid m}\varphi (\tfrac{m}{d})\mu _{d^2Y}. \end{aligned}$$

#### Theorem 1.3

Keep the notation as above. Let $x=p/q$ be a primitive rational number and let $\{y_n\}_{n\in {\mathbb {N}}}$ be a sequence of positive numbers.

1. If $y_n=c/n^2$ for some constant $c>0$, then for any $m\in {\mathbb {N}}_q$ and for any $\Psi \in C_c^{\infty }({\mathcal {M}})$
  $$\begin{aligned} \lim \limits _{\begin{array}{c} n\rightarrow \infty \\ \gcd (n,q)=1 \end{array}}\delta _{n,x,y_n}^{\mathrm{pr}}(\Psi )=\mu _{\tfrac{1}{cq^2}}(\Psi )\quad \text {and}\quad \lim \limits _{\begin{array}{c} n\rightarrow \infty \\ n\in {\mathbb {P}}_{m} \end{array}}\delta _{n,x,y_n}(\Psi )=\nu _{m,\tfrac{\gcd (m,q)^2}{cq^2}}(\Psi ). \end{aligned}$$
2. If $\lim \nolimits _{n\rightarrow \infty }n^2y_n=0$, then both sequences $\{{\mathcal {R}}_n(x,y_n)\}_{n\in {\mathbb {N}}}$ and $\{{\mathcal {R}}^{\mathrm{pr}}_n(x,y_n)\}_{n\in {\mathbb {N}}}$ fully escape to the cusp of ${\mathcal {M}}$.

Our next result shows that, similar to the rational translate case, equidistribution fails for generic translates as soon as $\{y_n\}_{n\in {\mathbb {N}}}$ decays logarithmically faster than $1/n^2$.

#### Theorem 1.4

Let $d_{{\mathcal {M}}}(\cdot ,\cdot )$ be the distance function on ${\mathcal {M}}$ induced from the hyperbolic distance function on ${\mathbb {H}}$. Fix $\Gamma z_0\in {\mathcal {M}}$. Let $\{y_n\}_{n\in {\mathbb {N}}}$ be a sequence of positive numbers satisfying $y_n\asymp 1/(n^2\log ^{\beta } n)$ for some fixed $0< \beta < 2$. Then for almost every $x\in {\mathbb {R}}/{\mathbb {Z}}$

1.10 $$\begin{aligned} \mathop {{\overline{\lim }}}_{n\rightarrow \infty }\frac{\inf _{\Gamma z\in {\mathcal {R}}_n(x,y_n)}d_{{\mathcal {M}}}\left( \Gamma z_0, \Gamma z\right) }{\log \log n}\ge \min \{\beta , 2-\beta \}. \end{aligned}$$

This implies that for almost every $x\in {\mathbb {R}}/{\mathbb {Z}}$, there exists an unbounded subsequence of ${\mathbb {N}}$ such that along this subsequence

$$\begin{aligned} \inf _{\Gamma z\in {\mathcal {R}}_n(x,y_n)}d_{{\mathcal {M}}}\left( \Gamma z_0, \Gamma z\right) \ge \left( \alpha -\epsilon \right) \log \log n, \end{aligned}$$

where $\alpha =\min \{\beta ,2-\beta \}$. That is, for almost every $x\in {\mathbb {R}}/{\mathbb {Z}}$, all the sample points ${\mathcal {R}}_{n}(x,y_n)$ (and hence also ${\mathcal {R}}_n^{\mathrm{pr}}(x,y_n)$) are moving towards the cusp of ${\mathcal {M}}$ along this subsequence, and eventually escape to the cusp as *n* in this subsequence goes to infinity.

Our proof of Theorem 1.4 relies on connections to Diophantine approximation theory. This viewpoint comes with inherent limitations; in the specific setting $y_n\asymp 1/(n^2\log ^\beta n)$, Khintchine’s approximation theorem guarantees full escape to the cusp almost surely, but this argument does not extend to any sequence $\{y_n\}_{n\in {\mathbb {N}}}$ that decays polynomially faster than $1/n^2$, see Sect. 1.3 for a more detailed discussion. It is thus interesting to study the cases when $\{y_n\}_{n\in {\mathbb {N}}}$ is beyond the ranges in Theorems 1.1 and  1.4.

Indeed, the rest of our results deal with sequences $\{y_n\}_{n\in {\mathbb {N}}}$ that can decay *arbitrarily fast*, and give both positive and negative results. This is the main novelty of this paper; the handling of cases in which the sample points can be *arbitrarily sparse* on the closed horocycles they lie on. We now state the main novel aspect of this paper:

#### Theorem 1.5

For any sequence of positive numbers $\{c_n\}_{n\in {\mathbb {N}}}$, there exists a sequence $\{y_n\}_{n\in {\mathbb {N}}}$ satisfying $0<y_n<c_n$ for each $n\in {\mathbb {N}}$ and such that for almost every $x\in {\mathbb {R}}/{\mathbb {Z}}$ the set of limiting measures of $\{\delta _{n,x,y_n}\}_{n\in {\mathbb {N}}}$ and $\{\delta _{n,x,y_n}^{\mathrm{pr}}\}_{n\in {\mathbb {N}}}$ both contain the uniform measure $\mu _{{\mathcal {M}}}$, the zero measure, and singular probability measures.

Theorem 1.5 is a sum of three more precise theorems, which each handles a specific limiting measure, and which we discuss in the next subsection.

### Discussion of the results

Our proofs of Theorems 1.1 and  1.2 rely on spectral estimates collected in the recent paper of Kelmer and Kontorovich [18], with a necessary refinement of [18, (3.6)] in the form of Proposition 3.3, which comes at the cost of a higher degree Sobolev norm. This strategy is standard and is also found in [4, 16, 27, 31], to name just a few recent papers on related problems. The analysis in [18] was carried out in a more general setting, namely for the congruence covers $\Gamma _0(p)\backslash {\mathbb {H}}$ with *p* a prime number. Theorem 1.1 can be extended to that more general setting, see Remark 3.11. With these spectral estimates in hand, we further prove an effective non-equidistribution result for rational translates from which part (1) of Theorem 1.3 follows, see Theorem 3.10. Part (2) of Theorem 1.3 is an easy application of the symmetry (1.6).

#### Remark 1.11

As was pointed out to us by Asaf Katz, we could also have used the estimates from [31, Proposition 3.1] in place of [18, Proposition 3.4], which in our specific setting, give the same equidistribution range (with a higher degree Sobolev norm). We also mention that the estimates in [31, Proposition 3.1] are valid in the setting of $\Gamma _0(q)\backslash {\text {SL}}_2({\mathbb {R}})$ with $q\in {\mathbb {N}}$, and thus imply an effective equidistribution result analogous to Theorem 1.1 in this generality.

As mentioned earlier, a generalization of the symmetry (1.6) is available for rational translates but breaks down for irrational translates. To handle irrational translates, we approximate them by rational ones to apply the symmetry relation, see Lemma 4.2. This is where Diophantine approximation kicks in. Similar ideas were also used in [27, Section 7] to construct counterexamples in their setting. In fact, we prove Theorem 1.4 by proving a more general result that captures the cusp excursion rates of the sample points ${\mathcal {R}}_n(x,y_n)$ in terms of the Diophantine properties of the translate *x*, see Theorem 4.3. Theorem 1.4 will then follow from Theorem 4.3 by imposing a Diophantine condition which ensures cusp excursion, while also holds for almost every translate thanks to Khintchine’s approximation theorem. This Diophantine condition accounts for the tight restrictions on $\{y_n\}_{n\in {\mathbb {N}}}$ in Theorem 1.4. On the other hand, assuming an even stronger Diophantine condition (which holds for a null set of translates), we can handle sequences decaying polynomially faster than $1/n^2$ with a much faster excursion rate towards the cusp, see Theorem 4.4. We also prove a non-equidistribution result (which, this time, holds for *every* *x*) when $y_n=c/n^2$ and the constant *c* is restricted to some range, see Theorem 4.5. The trade-off of this upgrade from Theorem 1.4 to the everywhere non-equidistribution result is that we can no longer prove the full escape to the cusp along subsequences as in Theorem 1.4.

As mentioned before, Theorem 1.5 follows from three more precise theorems which each handles a specific limiting measure. Our first result confirms equidistribution almost surely along a fixed subsequence of ${\mathbb {N}}$ for *any* sequence $\{y_n\}_{n\in {\mathbb {N}}}$ decaying at least polynomially.

#### Theorem 1.6

Fix $\alpha >0$. Then there exists a fixed unbounded subsequence ${\mathcal {N}}\subset {\mathbb {N}}$ such that for any sequence of positive numbers $\{y_n\}_{n\in {\mathbb {N}}}$ satisfying $y_n\ll n^{-\alpha }$ and for almost every $x\in {\mathbb {R}}/{\mathbb {Z}}$, both $\delta _{n,x,y_n}$ and $\delta ^{\mathrm{pr}}_{n,x,y_n}$ weakly converge to $\mu _{{\mathcal {M}}}$ as $n\in {\mathcal {N}}$ goes to infinity.

#### Remark 1.12

It will be clear from our proof that one can take ${\mathcal {N}}\subset {\mathbb {N}}$ to be any subsequence satisfying $\sum _{n\in {\mathcal {N}}}n^{-c}<\infty$ for some positive $c<\min \{\frac{\alpha }{2}, 1-2\theta \}$, e.g. we may take ${\mathcal {N}}=\left\{ \left\lfloor {n^{\kappa }}\right\rfloor \right\} _{n\in {\mathbb {N}}}$ for any $\kappa >1/\min \{\frac{\alpha }{2}, 1-2\theta \}$.

Theorem 1.6 follows from a second moment estimate for the discrepancies $|\delta _{n,x,y}-\mu _{{\mathcal {M}}}|$ and $|\delta _{n,x,y}^{\mathrm{pr}}-\mu _{{\mathcal {M}}}|$ along the closed horocycle ${\mathcal {H}}_y$ (Theorem 5.2) together with a standard Borel–Cantelli type argument. This was also the strategy used in [27] when studying the Kronecker sequences in (1.3). Along these lines, they deduce from spectral estimates the equidistribution for almost every $\beta \in {\mathbb {R}}$ along a fixed subsequence $\{n^k\}_{n\in {\mathbb {N}}}$ when $y_n\asymp n^{-\alpha }$ with $k\in {\mathbb {N}}$ depending on $\alpha >0$. Then, using a continuity argument, this result is upgraded to the equidistribution along the full sequence of positive integers, see [27, Section 4]. This continuity argument fails in our situation. Instead of applying directly spectral estimates to the second moment formulas, we express the latter in terms of certain Hecke operators (Proposition 5.1), and rely on available (spectral) bounds for their operator norm, see [10]. Contrarily to spectral estimates, the recourse to Hecke operators allows us to have a uniform subsequence ${\mathcal {N}}$ which is valid for all $\{y_n\}_{n\in {\mathbb {N}}}$ decaying at least polynomially.

Next, we show that there exists a sequence $\{y_n\}_{n\in {\mathbb {N}}}$ decaying arbitrarily rapidly such that for almost every *x*, ${\mathcal {R}}_n(x,y_n)$ (and thus also ${\mathcal {R}}_n^{\mathrm{pr}}(x,y)$) escapes to the cusp with a certain rate along subsequences.

#### Theorem 1.7

Fix $\Gamma z_0\in {\mathcal {M}}$. For any sequence of positive numbers $\left\{ c_n\right\} _{n\in {\mathbb {N}}}$, there exists a sequence $\{y_n\}_{n\in {\mathbb {N}}}$ satisfying $0<y_n< c_n$ for each $n\in {\mathbb {N}}$ and such that for almost every $x\in {\mathbb {R}}/{\mathbb {Z}}$

1.13 $$\begin{aligned} \mathop {{\overline{\lim }}}_{n\rightarrow \infty }\frac{\inf _{\Gamma z\in {\mathcal {R}}_n(x,y_n)}d_{{\mathcal {M}}}\left( \Gamma z_0, \Gamma z\right) }{\log \log n}\ge 1. \end{aligned}$$

Finally, we show that escape to the cusp is not the only obstacle to equidistribution.

#### Theorem 1.8

Let $m\in {\mathbb {N}}$ and $Y>0$ satisfy $m^2Y>1$. Let ${\mathbb {P}}_m\subset {\mathbb {N}}$ and $\nu _{m,Y}$ be as defined in (1.8) and (1.9) respectively. For any sequence of positive numbers $\{c_n\}_{n\in {\mathbb {P}}_{m}}$, there exists a sequence $\{y_n\}_{n\in {\mathbb {P}}_m}$ satisfying $0<y_n< c_n$ for all $n\in {\mathbb {P}}_m$ such that for almost every $x\in {\mathbb {R}}/{\mathbb {Z}}$, the set of limiting measures of $\{\delta _{n,x,y_n}\}_{n\in {\mathbb {P}}_m}$ contains $\nu _{m,Y}$.

#### Remark 1.14

We note that ${\mathbb {P}}_1$ is the set of prime numbers and $\nu _{1,Y}=\mu _Y$. Since

$$\begin{aligned} \delta ^{\mathrm{pr}}_{p,x,y}(\Psi )=\tfrac{p}{p-1}\delta _{p,x,y}(\Psi )+O(p^{-1}\Vert \Psi \Vert _{\infty }) \end{aligned}$$

whenever *p* is a prime number, when $m=1$ the conclusion of Theorem 1.8 also holds for the sequence $\{\delta _{n,x,y_n}^{\mathrm{pr}}\}_{n\in {\mathbb {P}}_1}$. We also note that it will be clear from our proof that Theorems 1.7 and 1.8 can be combined. In fact, our argument shows that there always exists a sequence $\{y_n\}_{n\in {\mathbb {N}}}$ decaying faster than any prescribed sequence such that for almost every $x\in {\mathbb {R}}/{\mathbb {Z}}$ the set of limiting measures of $\left\{ \delta _{n,x,y_n}\right\} _{n\in {\mathbb {N}}}$ contains the trivial measure and $\nu _{m,Y}$ for any finitely many pairs $(m,Y)\in {\mathbb {N}}\times {\mathbb {R}}_{>0}$ with $m^2Y>1$, see Remark 7.26. Moreover, in view of Theorem 1.6 if $y_n\ll n^{-\alpha }$ for some $\alpha >0$, then it also contains the hyperbolic area $\mu _{{\mathcal {M}}}$ almost surely.

For the rest of this introduction we describe the strategy of our proof to Theorem 1.7 (Theorem 1.8 follows from similar ideas). To detect cusp excursions, we study for each $n\in {\mathbb {N}}$ the occurrence of the events

1.15 $$\begin{aligned} \Gamma \left( x+\tfrac{j}{n}+iy_n\right) \in {\mathcal {C}}\quad \text {for all}\, 0\le j\le n-1, \end{aligned}$$

where ${\mathcal {C}}\subset {\mathcal {M}}$ is some fixed cusp neighborhood of ${\mathcal {M}}$. More precisely, we determine when the limsup set $I_{\infty }=\mathop {{\overline{\lim }}}_{n\rightarrow \infty }I_n$ is of full measure, where for each $n\in {\mathbb {N}}$,

$$\begin{aligned} I_n:=\{x\in {\mathbb {R}}/{\mathbb {Z}}:{\mathcal {R}}_n(x,y_n)\subset {\mathcal {C}}\} \end{aligned}$$

consists of translates $x\in {\mathbb {R}}/{\mathbb {Z}}$ for which the events in (1.15) occur. This requires to study the left regular $u_{1/n}$-action on ${\mathcal {C}}\subset {\mathcal {M}}$ and thus calls for the underlying lattice to be normalized by $u_{1/n}$. Therefore, we construct an explicit tower of coverings $\{\Gamma _n\backslash {\mathbb {H}}\}_{n\in {\mathbb {N}}}$ in which each $\Gamma _n$ is a congruence subgroup normalized by $u_{1/n}$. We note that the existence of such $\Gamma _n<\Gamma$ is the starting point of our proof and it relies on the assumption that $\Gamma ={\text {SL}}_2({\mathbb {Z}})$; this construction would fail for $\Gamma$ replaced by a non-arithmetic lattice.

The key ingredient of the proof will be a sufficient condition which states that if a point $\Gamma _n (x+iy_n)\in \Gamma _n\backslash {\mathbb {H}}$ visits a certain cusp neighborhood ${\mathcal {C}}_n$ on $\Gamma _n\backslash {\mathbb {H}}$, then the events in (1.15) will be realized for $x\in {\mathbb {R}}/{\mathbb {Z}}$, that is, $x\in I_n$, see Lemma 7.6. Using this sufficient condition, we can then relate the measure of $I_n$ to the proportion of certain closed horocycles on $\Gamma _n\backslash {\mathbb {H}}$ visiting the cusp neighborhood ${\mathcal {C}}_{n}\subset \Gamma _n\backslash {\mathbb {H}}$, which in turn, using the equidistribution of expanding closed horocycles on $\Gamma _n\backslash {\mathbb {H}}$, can be estimated for $y_n$ sufficiently small. Since the sets $I_n$ also need to satisfy certain quasi-independence conditions for $I_\infty$ to have full measure (Lemma 2.5), we need to apply the equidistribution of certain subsegments of the expanding closed horocycles on $\Gamma _n\backslash {\mathbb {H}}$. More precisely, at the *n*-th step these subsegments will be taken to be the sets $I_m$ for all $m<n$. These subsegment are finite disjoint unions of subintervals whose number and size depend sensitively on the height parameters $\{y_m\}_{m<n}$, see Remark 6.3. If there would exist an effective equidistribution result which would be insensitive to the geometry of these subsegments, that is, for which the error term depends only on the measure of these subsegments, then we would have an effective control on the sequence $\{y_n\}_{n\in {\mathbb {N}}}$ in Theorem 1.7 (and similarly also in Theorem 1.8). However, it is not clear to us whether one should expect such an effective equidistribution result.

Finally, we note that it was communicated to us by Strömbergsson that using a number theoretic interpretation of the aforementioned sufficient condition and some elementary estimates, one can alternatively prove Theorem 1.7 without going into these congruence covers, see Remark 7.17.

### Structure of the paper

In Sect. 2, we collect some preliminary results that will be needed in the rest of the paper. In Sect. 3, we prove a key spectral estimate (Proposition 3.3) and proceed to prove Theorems 1.1 and  1.2. In Sect. 4, we prove Theorems 4.3 and  4.5 by examining the connections between Diophantine approximations and cusp excursions on the modular surface. In Sect. 5, we prove Theorem 1.6 by proving a second moment bound using Hecke operators. In Sect. 6, we study the left regular action of a normalizing element on the set of cusp neighborhoods of a congruence cover of the modular surface. Building on the results, we prove Theorems 1.7 and  1.8 in Sect. 7.

### Notation

For two positive quantities *A* and *B*, we will use the notation $A\ll B$ or $A=O(B)$ to mean that there is a constant $c>0$ such that $A\le cB$, and we will use subscripts to indicate the dependence of the constant on parameters. We will write $A\asymp B$ for $A\ll B\ll A$. For any $z\in {\mathbb {H}}$ we denote by $e(z):=e^{2\pi iz}$. For any $n\in {\mathbb {N}}$, we denote by $\prod _{d\mid n}$ the product over all positive divisors of *n*, and by $$\prod _{\begin{array}{c} p\mid n\\ prime \end{array}}$$ the product over all prime divisors of *n*. For any $x\ge 0$ and $n\in {\mathbb {N}}$, $\sigma _x(n):=\sum _{d\mid n}d^x$ is the power-*x* divisor function which satisfies the estimate $\sigma _x(n)\ll _{\epsilon }n^{x+\epsilon }$ for any small $\epsilon >0$.

## Preliminaries

Let $G={\text {SL}}_2({\mathbb {R}})$. We consider the Iwasawa decomposition $G=NAK$ with

$$\begin{aligned} N=\left\{ u_x:x\in {\mathbb {R}}\right\} ,\quad A=\left\{ a_y:y>0\right\} ,\quad K=\left\{ k_{\theta }:0\le \theta <2\pi \right\} , \end{aligned}$$

where $$u_x=\left( {\begin{matrix} 1 &{} x\\ 0 &{} 1\end{matrix}}\right) $$, $$a_y=\left( {\begin{matrix} y^{1/2} &{} 0\\ 0 &{} y^{-1/2}\end{matrix}}\right) $$ and $$k_{\theta }=\left( {\begin{matrix} \cos \theta &{} \sin \theta \\ -\sin \theta &{} \cos \theta \end{matrix}}\right) $$ respectively. Under the coordinates $g=u_xa_yk_{\theta }$ on *G*, the Haar measure is given (up to scalars) by

$$\begin{aligned} d g=y^{-2}dxdyd\theta . \end{aligned}$$

The group *G* acts on the upper half plane ${\mathbb {H}}=\{z=x+iy\in {\mathbb {C}}:y>0\}$ via the Möbius transformation: $g z=\frac{az+b}{cz+d}$ for any $$g=\left( {\begin{matrix} a &{} b\\ c &{} d\end{matrix}}\right) \in G$$ and $z\in {\mathbb {H}}$. This action preserves the hyperbolic area $d\mu (z)=y^{-2}dxdy$ and induces an identification between *G*/*K* and ${\mathbb {H}}$.

Let $\Gamma < G$ be a *lattice*, that is, $\Gamma$ is a discrete subgroup of *G* such that the corresponding hyperbolic surface $\Gamma \backslash {\mathbb {H}}$ has finite area (with respect to $\mu$). We denote by $\mu _{\Gamma }:=\mu (\Gamma \backslash {\mathbb {H}})^{-1}\mu$ the normalized hyperbolic area on $\Gamma \backslash {\mathbb {H}}$ such that $\mu _{\Gamma }(\Gamma \backslash {\mathbb {H}})=1$. We note that when $\Gamma ={\text {SL}}_2({\mathbb {Z}})$ then $\mu _{\Gamma }=\mu _{{\mathcal {M}}}$ with $\mu _{{\mathcal {M}}}$ the normalized hyperbolic area on the modular surface ${\mathcal {M}}$ given as in the introduction. We note that in this case it is well known $\mu ({\mathcal {M}})=\pi /3$, and hence

2.1 $$\begin{aligned} d\mu _{{\mathcal {M}}}(z)=\frac{3}{\pi }\frac{dxdy}{y^2}. \end{aligned}$$

Using the above identification between ${\mathbb {H}}$ and *G*/*K* we can identify the hyperbolic surface $\Gamma \backslash {\mathbb {H}}$ with the locally symmetric space $\Gamma \backslash G/K$. We can thus view subsets of $\Gamma \backslash {\mathbb {H}}$ as right *K*-invariant subsets of $\Gamma \backslash G$. Similarly, we can view functions on $\Gamma \backslash {\mathbb {H}}$ as right *K*-invariant functions on $\Gamma \backslash G$. We note that using the above description of the Haar measure, the probability Haar measure on $\Gamma \backslash G$ (when restricted to the sub-family of right *K*-invariant subsets) coincides with the normalized hyperbolic area $\mu _{\Gamma }$ on $\Gamma \backslash {\mathbb {H}}$.

### Sobolev norms

In this subsection we record some useful properties about Sobolev norms. Let $\mathfrak {g}=\mathfrak {sl}_2({\mathbb {R}})$ be the Lie algebra of *G*. Fix a basis ${\mathscr {B}}=\{X_1, X_2, X_3\}$ for $\mathfrak {g}$, and given a smooth test function $\Psi \in C^{\infty }(\Gamma \backslash G)$ we define the “$L^p$, order-*d*” Sobolev norm ${\mathcal {S}}^{\Gamma }_{p,d}(\Psi )$ as

$$\begin{aligned} {\mathcal {S}}^{\Gamma }_{p,d}(\Psi ):=\sum _{\text {ord}({\mathscr {D}})\le d}\Vert {\mathscr {D}}\Psi \Vert _{L^p(\Gamma \backslash G)}, \end{aligned}$$

where ${\mathscr {D}}$ runs over all monomials in ${\mathscr {B}}$ of order at most *d*, and the $L^p$-norm is with respect to the normalized Haar measure on $\Gamma \backslash G$.

For any $\Psi \in C^{\infty }(\Gamma \backslash G)$ (which we think of a smooth left $\Gamma$-invariant function on *G*) and for any $h\in G$ we denote by $L_h\Psi (g):=\Psi (h^{-1}g)$ the left regular *h*-action on $\Psi$. It is easy to check that $L_h\Psi \in C^{\infty }(h\Gamma h^{-1}\backslash G)$, and since taking Lie derivatives commutes with the left regular action, we have

2.2 $$\begin{aligned} {\mathcal {S}}_{p,d}^{\Gamma }(\Psi )={\mathcal {S}}_{p,d}^{h\Gamma h^{-1}}(L_h\Psi ). \end{aligned}$$

Next we note that using the product rule for Lie derivatives (see e.g. [21, p. 90]), the triangle inequality and the Cauchy–Schwarz inequality, for any monomial ${\mathscr {D}}$ of order $k{{\le d}}$ we have for any smooth functions $\Psi _1,\Psi _2\in C^{\infty }(\Gamma \backslash G)$

$$\begin{aligned} \Vert {\mathscr {D}}\Psi _1\Psi _2\Vert _{L^p(\Gamma \backslash G)}\ll _{k}{\mathcal {S}}_{2p,{{k}}}^{\Gamma }(\Psi _1){\mathcal {S}}_{2p,{{k}}}^{\Gamma }(\Psi _2){{\le {\mathcal {S}}_{2p,d}^{\Gamma }(\Psi _1){\mathcal {S}}_{2p,d}^{\Gamma }(\Psi _2).}} \end{aligned}$$

In particular this implies that

2.3 $$\begin{aligned} {\mathcal {S}}^{\Gamma }_{p,d}(\Psi _1\Psi _2)\ll _{d}{\mathcal {S}}_{2p,d}^{\Gamma }(\Psi _1){\mathcal {S}}_{2p,d}^{\Gamma }(\Psi _2). \end{aligned}$$

Finally, we note that if $\Gamma '<\Gamma$ is a finite-index subgroup of $\Gamma$, then there is a natural embedding $C^{\infty }(\Gamma \backslash G)\hookrightarrow C^{\infty }(\Gamma '\backslash G)$ since each $\Psi \in C^{\infty }(\Gamma \backslash G)$ can be viewed as a smooth left $\Gamma '$-invariant function on *G*. Since the Sobolev norms are defined with respect to the normalized Haar measure on the corresponding homogeneous space, we have for $\Gamma '< \Gamma$ of finite index and $\Psi \in C^{\infty }(\Gamma \backslash G)$

2.4 $$\begin{aligned} {\mathcal {S}}_{p,d}^{\Gamma '}(\Psi )={\mathcal {S}}_{p,d}^{\Gamma }(\Psi ). \end{aligned}$$

### Spectral decomposition

Let $\Gamma < G$ be a *non-uniform* lattice, that is, $\Gamma$ is a lattice and $\Gamma \backslash {\mathbb {H}}$ is not compact. Let $\Delta =-y^2(\frac{\partial }{\partial x^2}+\frac{\partial }{\partial y^2})$ be the hyperbolic Laplace operator. It is a second order differential operator acting on $C^{\infty }(\Gamma \backslash {\mathbb {H}})$ and extends uniquely to a self-adjoint and positive semi-definite operator on $L^2(\Gamma \backslash {\mathbb {H}})$. Since $\Gamma$ is non-uniform, the spectrum of $\Delta$ is composed of a continuous part (spanned by Eisenstein series) and a discrete part (spanned by Maass forms) which further decomposes as the cuspidal spectrum and the residual spectrum. The residual spectrum always contains the constant functions (coming from the trivial pole of the Eisenstein series). If $\Gamma$ is a *congruence subgroup*, that is, $\Gamma$ contains a *principal congruence subgroup*

$$\begin{aligned} \Gamma (n):=\left\{ \gamma \in {\text {SL}}_2({\mathbb {Z}}):\gamma \equiv I_2 \ (\mathrm {mod}\ n)\right\} \end{aligned}$$

for some $n\in {\mathbb {N}}$, then the residual spectrum consists only of the constant functions, see e.g. [15, Theorem 11.3].

Let $\{\phi _k\}$ be an orthonormal basis of the space of cusp forms that are eigenfunctions of the Laplace operator $\Delta$. Explicitly, for each $\phi _k$ there exists $\lambda _k\ge 0$ such that

$$\begin{aligned} \Delta \phi _k=\lambda _k \phi _k = s_k(1-s_k)\phi _k=\left( \tfrac{1}{4}+r_k^2\right) \phi _k. \end{aligned}$$

Selberg’s eigenvalue conjecture states that for congruence subgroups, $\lambda _k\ge 1/4$, or equivalently, there is no $r_k\in i(0,1/2)$. Selberg’s conjecture is known to be true for the modular surface ${\mathcal {M}}$, and more generally, the best known bound towards this conjecture is currently $\lambda _k\ge \tfrac{1}{4}-\theta ^2$, with $\theta =7/64$, which follows from the bound of Kim and Sarnak towards the Ramanujan conjecture, see [19, p. 176].

Let now $\Gamma ={\text {SL}}_2({\mathbb {Z}})$. In the notation introduced at the beginning of this section, the Eisenstein series for the modular group $\Gamma$ at the cusp $\infty$ is defined for ${\mathfrak {Re}}(s)>1$ by

2.5 $$\begin{aligned} E(z,s)=\sum _{\gamma \in (\Gamma \cap \pm N)\backslash \Gamma }{\mathfrak {Im}}(\gamma z)^s \end{aligned}$$

with a meromorphic continuation to $s\in {\mathbb {C}}$. Moreover, for any $s\in {\mathbb {C}}$, $E(\cdot , s)$ is an eigenfunction of the Laplace operator with eigenvalue $s(1-s)$.

Let $\Psi \in L^2({\mathcal {M}})$ and we have the following spectral decomposition (see [15, Theorems 4.7 and 7.3])

2.6 $$\begin{aligned} \Psi (z)&=\mu _{{\mathcal {M}}}(\Psi )+\sum _{r_k\ge 0}\langle \Psi ,\phi _k\rangle \phi _k(z)\nonumber \\&\quad \quad +\frac{1}{4\pi }\int _{-\infty }^{\infty }\langle \Psi , E(\cdot , \tfrac{1}{2}+ir)\rangle E(z,\tfrac{1}{2}+ir)dr, \end{aligned}$$

where the convergence holds in the $L^2$-norm topology, and is pointwise if $\Psi \in C_c^{\infty }({\mathcal {M}})$. As a direct consequence we have for $\Psi \in L^2({\mathcal {M}})$,

2.7 $$\begin{aligned} \Vert \Psi \Vert _2^2=\left| \mu _{{\mathcal {M}}}(\Psi )\right| ^2+\sum _{r_k\ge 0}\left| \langle \Psi ,\phi _k\rangle \right| ^2+\frac{1}{4\pi }\int _{-\infty }^{\infty }\left| \langle \Psi ,E(\cdot ,\tfrac{1}{2}+ir)\rangle \right| ^2dr. \end{aligned}$$

### Hecke operators

The spectral theory of ${\mathcal {M}}$ has extra structure due to the existence of *Hecke operators*. The main goal of this subsection is to prove an operator norm bound for Hecke operators and the main reference is [15, Section 8.5]. For any $n\in {\mathbb {N}}$ define the set

2.8 $$\begin{aligned} {\mathcal {L}}_n:=\left\{ n^{-1/2}g :g\in M_2({\mathbb {Z}}),\; \det (g)=n\right\} \subset G, \end{aligned}$$

where $M_2({\mathbb {Z}})$ is the space of two by two integral matrices. The *n-th Hecke operator* $T_n$ is defined by that for any $\Psi \in L^2({\mathcal {M}})$

$$\begin{aligned} T_{n}(\Psi )(z)=\frac{1}{n^{1/2}}\sum _{\gamma \in \Gamma \backslash {\mathcal {L}}_n}\Psi (\gamma z). \end{aligned}$$

The Hecke operator $T_n$ is a self-adjoint operator on $L^2({\mathcal {M}})$ and since $T_n$ commutes with the Laplace operator $\Delta$ (since $\Delta$ is defined via right multiplication and $T_n$ is defined via left multiplication) the orthonormal basis of the space of cusp forms $\{\phi _k\}$ can be chosen consisting of joint eigenfunctions of all $T_n$, that is,

$$\begin{aligned} T_n \phi _k=\lambda _{\phi _k}(n)\phi _k. \end{aligned}$$

On the other hand, for any $r\in {\mathbb {R}}$ the Eisenstein series $E(z, 1/2+ir)$ is an eigenfunction of $T_n$ with eigenvalue $\lambda _r(n):=\sum _{d\mid n}\left( \frac{n}{d^2}\right) ^{ir}$, see [15, Equation (8.33)]. It is clear that $\left| \lambda _r(n)\right| \le \sigma _0(n)$ with $\sigma _0(n)$ the divisor function. For the eigenvalue of cusp forms it is conjectured (Ramanujan-Petersson) that for any above $\phi _k$ and for any $n\in {\mathbb {N}}$

$$\begin{aligned} \left| \lambda _{\phi _k}(n)\right| \le \sigma _0(n). \end{aligned}$$

The aforementioned bound of Sarnak and Kim [19] implies that

$$\begin{aligned} \left| \lambda _{\phi _k}(n)\right| \le \sigma _0(n)n^{7/64}. \end{aligned}$$

Using these bounds on eigenvalues and the above spectral decomposition (2.6) and (2.7) we have the following bound on the operator norm of the Hecke operator, see also [10, pp. 172-173].

#### Proposition 2.1

For any $\Psi \in L^2({\mathcal {M}})$ and for any $n\in {\mathbb {N}}$ we have

$$\begin{aligned} \langle \Psi _0, T_n(\Psi _0)\rangle _{L^2({\mathcal {M}})}\ll _{\epsilon }n^{\theta +\epsilon }\Vert \Psi \Vert _2^2, \end{aligned}$$

where $\Psi _0:=\Psi -\mu _{{\mathcal {M}}}(\Psi )$ and $\theta =7/64$ as before.

#### Hecke operators attached to a group element

Let $\Gamma ={\text {SL}}_2({\mathbb {Z}})$ and let ${\mathcal {M}}=\Gamma \backslash {\mathbb {H}}$ be the modular surface as above. There is another type of Hecke operators on $L^2({\mathcal {M}})$ defined via a group element in ${\text {SL}}_2({\mathbb {Q}})$. Namely, for each $h\in {\text {SL}}_2({\mathbb {Q}})$ the *Hecke operator attached to* *h*, denoted by ${\widetilde{T}}_h$, is defined by that for any $\Psi \in L^2({\mathcal {M}})$

2.9 $$\begin{aligned} {\widetilde{T}}_h(\Psi )(z)=\frac{1}{\# (\Gamma \backslash \Gamma h\Gamma )}\sum _{g\in \Gamma \backslash \Gamma h\Gamma }\Psi (g z), \end{aligned}$$

where $\Gamma h\Gamma =\left\{ \gamma _1 h\gamma _2:\gamma _1,\gamma _2\in \Gamma \right\}$ is the double coset attached to *h*. We note that ${\widetilde{T}}_h$ is well-defined since $\Psi$ is left $\Gamma$-invariant.

For our purpose, we will need another expression for ${\widetilde{T}}_h$. For any $h\in {\text {SL}}_2({\mathbb {Q}})$ we denote by $\Gamma ^h:=\Gamma \cap h^{-1}\Gamma h$. We note that the map from $\Gamma$ to $\Gamma \backslash \Gamma h\Gamma$ sending $\gamma \in \Gamma$ to $\Gamma h\gamma$ induces an identification between $\Gamma ^h\backslash \Gamma$ and $\Gamma \backslash \Gamma h\Gamma$. This identification induces the following alternative expression for ${\widetilde{T}}_h$:

2.10 $$\begin{aligned} {\widetilde{T}}_h(\Psi )(g)=\frac{1}{[\Gamma : \Gamma ^h]}\sum _{\gamma \in \Gamma ^h\backslash \Gamma }\Psi (h\gamma g). \end{aligned}$$

It is clear from the definition that ${\widetilde{T}}_h$ is defined only up to representatives for the double coset $\Gamma h\Gamma$, that is, ${\widetilde{T}}_h={\widetilde{T}}_{h'}$ whenever $\Gamma h\Gamma =\Gamma h'\Gamma$. For a fixed $h\in {\text {SL}}_2({\mathbb {Q}})$, we call $n\in {\mathbb {N}}$ *the degree of* *h* if *n* is the smallest positive integer such that $nh\in M_2({\mathbb {Z}})$. Using elementary column and row operations one can see that for $h\in {\text {SL}}_2({\mathbb {Q}})$ with degree *n*

2.11 $$\begin{aligned} \Gamma h\Gamma =\Gamma {\text {diag}}(1/n,n)\Gamma =\left\{ n^{-1}g : g\in M_2({\mathbb {Z}}),\; det(g)=n^2,\; \gcd (g)=1\right\} {{\subset G}},\nonumber \\ \end{aligned}$$

where $\gcd (g)$ is the greatest common divisor of the entries of *g*. Thus we can parameterize the Hecke operators by their degrees, that is, we will denote by ${\widetilde{T}}_n:={\widetilde{T}}_h$ for any $h\in {\text {SL}}_2({\mathbb {Q}})$ with degree *n*. We also note that by direct computation when $h={\text {diag}}(1/n,n)$ we have $\Gamma ^h=\Gamma _0(n^2)$, implying that for any $h\in {\text {SL}}_2({\mathbb {Q}})$ with degree *n* (see e.g. [6, Section 1.2])

2.12 $$\begin{aligned} \nu _n:=\# (\Gamma \backslash \Gamma h\Gamma )=[\Gamma : \Gamma ^h]=[\Gamma : \Gamma _0(n^2)]=n^2\prod _{\begin{array}{c} p\mid n\\ \text {prime} \end{array}}\left( 1+p^{-1}\right) . \end{aligned}$$

Now using the description (2.11) we have the double coset decomposition

$$\begin{aligned} {\mathcal {L}}_{n^2}=\bigsqcup _{d\mid n}\Gamma \begin{pmatrix} d^{-1} &{} 0\\ 0 &{} d\end{pmatrix}\Gamma . \end{aligned}$$

This decomposition together with the definitions (2.8), (2.9) and (2.12) implies the relation

$$\begin{aligned} nT_{n^2}=\sum _{d| n}\nu _d{\widetilde{T}}_{d}. \end{aligned}$$

Thus by the Möbius inversion formula we have

2.13 $$\begin{aligned} {\widetilde{T}}_{n}=\frac{n}{\nu _n}\sum _{d\mid n}\frac{\mu (d)}{d}T_{n^2/d^2}. \end{aligned}$$

Using this relation and Proposition 2.1 we can prove the following operator norm bounds for ${\widetilde{T}}_n$ which we will later use, see also [3, Theorem 1.1] for such bounds in a much greater generality.

##### Proposition 2.2

Keep the notation as in Proposition 2.1. For any $\Psi \in L^2({\mathcal {M}})$ and for any $n\in {\mathbb {N}}$ we have

$$\begin{aligned} \langle \Psi _0, {\widetilde{T}}_n(\Psi _0)\rangle _{L^2({\mathcal {M}})}\ll _{\epsilon }n^{-1+2\theta +\epsilon }\Vert \Psi \Vert _2^2. \end{aligned}$$

##### Proof

By Proposition 2.1 and using the relation (2.13), the trivial estimates $|\mu (d)|\le 1$ and $\nu _n\ge n^2$ and the triangle inequality we have

$$\begin{aligned} \langle \Psi _0, {\widetilde{T}}_n(\Psi _0)\rangle&\le n^{-2}\sum _{d| n}(n/d)\langle \Psi _0, T_{n^2/d^2}(\Psi _0)\rangle \ll _{\epsilon } n^{-2}\sum _{d\mid n}(n/d)^{1+2\theta +2\epsilon }\Vert \Psi \Vert _2^2\\&=n^{-1+2\theta +2\epsilon }\sigma _{-1+2\theta +2\epsilon }(n)\Vert \Psi \Vert _2^2\ll _{\epsilon }n^{-1+2\theta +\epsilon }\Vert \Psi \Vert _2^2. \end{aligned}$$

$$\square $$

### Equidistribution of subsegments of expanding closed horocycles

We record a special case of Sarnak’s result [28, Theorem 1] on effective equidistribution of expanding closed horocycles, namely:

#### Proposition 2.3

Let $\Gamma <{\text {SL}}_2({\mathbb {Z}})$ be a congruence subgroup and assume that $\Gamma$ has a cusp at $\infty$ with width one. Then for any $\Psi \in C^{\infty }(\Gamma \backslash {\mathbb {H}})\cap L^2(\Gamma \backslash {\mathbb {H}})$ satisfying $\Vert \Delta \Psi \Vert _2<\infty$ and for any $0<y<1$ we have

2.14 $$\begin{aligned} \left| \int _0^1\Psi (x+ iy)dx-\mu _{\Gamma }(\Psi )\right| \ll \Vert \Psi \Vert _2^{3/4}\Vert \Delta \Psi \Vert _2^{1/4}y^{1/2}, \end{aligned}$$

where the implied constant is absolute, independent of $\Gamma$, $\Psi$ and *y*, and the $L^2$-norm is with respect to the normalized hyperbolic area $\mu _{\Gamma }$.

#### Remark 2.15

We omit the proof here and refer the reader to [18, (3.5)]. We note that while [18] only deals with the case when $\Gamma =\Gamma _0(p)$ with *p* a prime number, the proof there works for general congruence subgroups, given that they have trivial residual spectrum; see [15, Theorem 11.3].

We will also need the following (non-effective) equidistribution result replacing the whole closed horocycle by a fixed subsegment:

#### Proposition 2.4

Let $\Gamma <{\text {SL}}_2({\mathbb {Z}})$ be as in Proposition 2.3. Let $I\subset (0,1)$ be an open interval, then for any $\Psi \in C_c(\Gamma \backslash {\mathbb {H}})$ we have

2.16 $$\begin{aligned} \lim \limits _{y\rightarrow 0^+}\frac{1}{|I|}\int _{I}\Psi (x+iy)dx=\mu _{\Gamma }(\Psi ). \end{aligned}$$

The proof of Proposition 2.4 uses Margulis’ thickening trick [25] and mixing property of the geodesic flow on the unit tangent bundle of $\Gamma \backslash {\mathbb {H}}$; this approach is also effective, see e.g. [17, Proposition 2.3]. A proof of (2.16) using spectral methods was also sketched in [12, Theorem 1$'$]. We also note that both equidistribution results in Propositions 2.3 and  2.4 can be lifted to the unit tangent bundle of $\Gamma \backslash {\mathbb {H}}$ (with necessary modifications to the error term in (2.14)); since we will be only working in the hyperbolic surface level, we state these two results in the current format for convenience of our discussion. We further refer the reader to [13, 30] for some much stronger effective equidistribution results regarding long enough (varying) subsegments on expanding closed horocycles.

#### Remark 2.17

Proposition 2.4 can be equivalently stated as following: For any fixed open interval $I\subset (0,1)$, the measures $\mu _{I,y}$ weakly converge to $\mu _{\Gamma }$ as $y\rightarrow 0^+$, where for any $y\in (0,1)$ and $\Psi \in C_c(\Gamma \backslash {\mathbb {H}})$, $\mu _{I,y}(\Psi ):=\frac{1}{|I|}\int _I\Psi (x+iy)dx$. Thus by the Portmanteau theorem, (2.16) extends to $\Psi =\chi _B$ with $B\subset \Gamma \backslash {\mathbb {H}}$ a Borel subset with boundary of measure zero. More generally, let $\rho : [0, 1)\rightarrow {\mathbb {R}}$ be a Riemann integrable function. Since $\rho$ can be weakly approximated from both above and below by step functions, we have

$$\begin{aligned} \lim \limits _{y\rightarrow 0^+}\int _{0}^1\rho (x)\chi _B(x+iy)dx=\mu _{\Gamma }(B)\int _0^1\rho (x)dx \end{aligned}$$

with $B\subset \Gamma \backslash {\mathbb {H}}$ a Borel set with boundary of measure zero.

### A quantitative Borel–Cantelli lemma

Finally we record here a quantitative Borel–Cantelli lemma which ensures for the limsup set of certain sequence of events to have full measure given certain quasi-independence conditions.

#### Lemma 2.5

[29, Chapter I, Lemma 10] Let $(X,{\mathcal {B}}, \nu )$ be a probability space with ${\mathcal {B}}$ a $\sigma$-algebra of subsets of *X* and $\nu : X\rightarrow [0,1]$ a probability measure on *X* with respect to ${\mathcal {B}}$. Let $\{A_i\}_{i\in {\mathbb {N}}}$ be a sequence of measurable subsets in ${\mathcal {B}}$. For any $n,m\in {\mathbb {N}}$ we denote by $R_{n,m}:=\nu (A_n\cap A_m)-\nu (A_n)\nu (A_m)$. Suppose that

2.18 $$\begin{aligned} \exists \ C>0 \,\text {such that for all}\, k_2>k_1\ge 1, \sum _{n,m=k_1}^{k_2}R_{n,m}\le C\sum _{n=k_1}^{k_2}\nu (A_n), \end{aligned}$$

then $\sum _{n\in {\mathbb {N}}}\nu (A_n)=\infty$ implies that $\nu \left( \mathop {{\overline{\lim }}}_{n\rightarrow \infty }A_n\right) =1$.

#### Remark 2.19

Keep the notation as in Lemma 2.5. It was shown in [20, Proposition 5.4] that if

$$\begin{aligned} \exists C'>0 \,\text {and}\, \eta >1 \,\text {such that for any}\, n\ne m, R_{n,m}\le C'\frac{\sqrt{\nu (A_n)\nu (A_m)}}{|n-m|^{\eta }}, \end{aligned}$$

then the sequence $\{A_i\}_{i\in {\mathbb {N}}}$ satisfies the condition (2.18).

We will use the following slightly modified version of quantitative Borel–Cantelli lemma which has the flexibility to consider sequence of measurable sets $\{A_n\}_{n\in {\mathbb {S}}}$ indexed by a general unbounded subset ${\mathbb {S}}\subset {\mathbb {N}}$.

#### Corollary 2.6

Let $(X,{\mathcal {B}},\nu )$ be as in Lemma 2.5. Let ${\mathbb {S}}\subset {\mathbb {N}}$ be an unbounded subset and let $\{A_n\}_{n\in {\mathbb {S}}}$ be a sequence of measurable subsets in ${\mathcal {B}}$. Suppose that

2.20 $$\begin{aligned} \exists C'>0 \,\text {and}\, \eta >1 \,\text {such that}\, \forall n,m\in {\mathbb {S}} \,\text {with}\, m< n, R_{n,m}\le C'\frac{\nu (A_n)\nu (A_m)}{n^{\eta }}, \end{aligned}$$

then $\sum _{n\in {\mathbb {S}}}\nu (A_n)=\infty$ implies that $$\nu \left( \mathop {{\overline{\lim }}}_{\begin{array}{c} n\in {\mathbb {S}}\\ n\rightarrow \infty \end{array}}A_n\right) =1$$.

#### Proof

For any $i\in {\mathbb {N}}$ let $a_i\in {\mathbb {S}}$ be the *i*-th integer in ${\mathbb {S}}$ and let $B_i:=A_{a_i}$. For any $i,j\in {\mathbb {N}}$ let $R'_{i,j}:=\nu (B_i\cap B_j)-\nu (B_i)\nu (B_j)$ so that $R_{i,j}'=R_{a_i,a_j}$. Then by for any $i<j$ we have

$$\begin{aligned} R'_{i,j}=R_{a_i,a_j}\le C'\frac{\nu (A_{a_i})\nu (A_{a_j})}{a_j^{\eta }}= C'\frac{\nu (B_i)\nu (B_j)}{a_j^{\eta }}<C'\frac{\sqrt{\nu (B_i)\nu (B_j)}}{|i-j|^{\eta }}, \end{aligned}$$

where for the first inequality we used the assumption (2.20) and for the second inequality we used the estimates $a_j\ge j> j-i$ and $\sqrt{\nu (B_i)\nu (B_j)}\le 1$. Thus in view of Remark 2.19 and Lemma 2.5 we have $\sum _{i\in {\mathbb {N}}}\nu (B_i)=\infty$ implies that $\nu \left( \mathop {{\overline{\lim }}}_{i\rightarrow \infty }B_i\right) =1$ which is equivalent to the conclusion of this corollary in view of the relation $B_i=A_{a_i}$. $\square$

## Equidistribution range

Let ${\mathcal {M}}={\text {SL}}_2({\mathbb {Z}})\backslash {\mathbb {H}}$. Since we fix $\Gamma ={\text {SL}}_2({\mathbb {Z}})$ throughout this section, we abbreviate the Sobolev norm ${\mathcal {S}}_{p,d}^{\Gamma }$ by ${\mathcal {S}}_{p,d}$. In this section, we prove Theorems 1.1 and  1.2. The main ingredient of our proof is an explicit bound of Fourier coefficients which follows from a slight modification of the estimates obtained in [18].

### Bounds on Fourier coefficients

Let $\Psi \in C_c^{\infty }({\mathcal {M}})$. Since $\Psi$ is left $\Gamma$-invariant, it is invariant under the transformation determined by $u_1:z\mapsto z+1$, and it thus has a Fourier expansion for $\Psi$ in the variable $x={\mathfrak {Re}}(z)$:

3.1 $$\begin{aligned} \Psi (x+iy)=\sum _{m\in {\mathbb {Z}}}a_{\Psi }(m,y)e(mx), \end{aligned}$$

where

$$\begin{aligned} a_{\Psi }(m,y)=\int _0^1\Psi (x+iy)e(-mx)dx. \end{aligned}$$

Similarly we denote by $a_{\phi _k}(m,y)$ and *a*(*s*; *m*, *y*) the *m*th Fourier coefficients of the Hecke-Maass form $\phi _k$ and the Eisenstein series $E(\cdot , s)$ respectively. Estimates on these Fourier coefficients yield, via the spectral expansion (2.6), estimates on the Fourier coefficients of $\Psi$. Namely,

$$\begin{aligned} a_{\Psi }(m,y)=\sum _{r_k\ge 0}\langle \Psi ,\phi _k\rangle a_{\phi _k}(m,y)+\frac{1}{4\pi }\int _{-\infty }^{\infty }\langle \Psi , E(\cdot , \tfrac{1}{2}+ir)\rangle a(\tfrac{1}{2}+ir;m,y)dr. \end{aligned}$$

We record the following bounds for $a_{\phi _k}(m,y)$ and *a*(*s*; *m*, *y*):

#### Lemma 3.1

[18, Lemmata 3.7 and 3.13] For any $m\ne 0$ and for any $\epsilon >0$ we have

3.2 $$\begin{aligned} |a_{\phi _k}(m,y)|\ll _{\epsilon }|m|^{\theta }y^{1/2-\epsilon }(r_k+1)^{-1/3+\epsilon }\min \{1,e^{\pi r_k/2-2\pi |m|y}\}, \end{aligned}$$

and

3.3 $$\begin{aligned} |a\left( \tfrac{1}{2}+ir;m,y\right) |\ll _{\epsilon }y^{1/2-\epsilon }(1+|r|)^{-1/3+\epsilon }\min \{1,e^{\pi |r|/2-2\pi |m| y}\}, \end{aligned}$$

where $\theta =7/64$ is the best known bound towards the Ramanujan conjecture as before.

#### Remark 3.4

Contrarily to [18] that uses the trivial bound $\min \{1,e^{\pi r/2-2\pi |m|y}\}\le 1$, we keep this term.

#### Proposition 3.2

[18, Proposition 3.4] For any $\Psi \in C_c^\infty ({\mathcal {M}})$, we have that

3.5 $$\begin{aligned} a_{\Psi }(0,y)\ =\ \mu _{{\mathcal {M}}}(\Psi ) + O\left( \Vert \Psi \Vert _2^{3/4}\Vert \Delta \Psi \Vert _2^{1/4}y^{1/2}\right) . \end{aligned}$$

Moreover, for any $m\ne 0$, and any $\epsilon >0$ and any $\alpha _0>5/3$, we have

3.6 $$\begin{aligned} a_{\Psi }(m,y)\ \ll _{\alpha _0,\epsilon ,p} {\mathcal {S}}_{\alpha _0}(\Psi )y^{1/2-\epsilon }|m|^\theta , \end{aligned}$$

where ${\mathcal {S}}_{\alpha _0}$ is a Sobolev norm of degree $\alpha _0$.

#### Remark 3.7

The Sobolev norm ${\mathcal {S}}_{\alpha _0}$ is explicit from the proof of [18, Proposition 3.4]: Writing $\alpha _0=5/3+\epsilon$ with $\epsilon >0$, then ${\mathcal {S}}_{\alpha _0}(\Psi )={\mathcal {S}}_{2,0}(\Psi )^{2/3-\epsilon /2}{\mathcal {S}}_{2,2}(\Psi )^{1/3+\epsilon /2}$ for any $\Psi \in C_c^{\infty }({\mathcal {M}})$. In particular, using the estimate ${\mathcal {S}}_{2,0}(\Psi )\le {\mathcal {S}}_{2,2}(\Psi )$ we have ${\mathcal {S}}_{\alpha _0}(\Psi )\le {\mathcal {S}}_{2,2}(\Psi )$.

The following refinement of this last estimate allows to estimate the Fourier coefficients when $|m|>y^{-1}$ is large. This refinement is crucial for our later results, and the price we pay is a Sobolev norm of higher degree.

#### Proposition 3.3

Let $\Psi \in C_c^{\infty }({\mathcal {M}})$. Whenever $|m|y>1$ and for any $\epsilon >0$, we have

$$\begin{aligned} |a_{\Psi }(m,y)|\ll _{\epsilon }{\mathcal {S}}_{2,2}(\Psi ) |m|^{-4/3+\theta +\epsilon }y^{-5/6}. \end{aligned}$$

#### Proof

For the contribution from the cusp forms we apply the bound (3.2) to the Fourier coefficients and the bound

3.8 $$\begin{aligned} \min \{1,e^{\pi r/2-2\pi |m|y}\}\le \left\{ \begin{array}{ll} e^{-\pi |m|y} &{} 0\le r\le 2|m|y\\ 1 &{} r> 2|m|y, \end{array}\right. \end{aligned}$$

and the relation $\langle \Delta \Psi , \phi _k\rangle =\langle \Psi , \Delta \phi _k\rangle =(1/4+r_k^2)\langle \Psi , \phi _k\rangle$ to get that

3.9 $$\begin{aligned}&\left| \sum _{r_k\ge 0}\langle \Psi ,\phi _k\rangle a_{\phi _k}(m,y)\right| \ll _{\epsilon } \sum _{0\le r_k\le 2|m|y}\left| \langle \Psi , \phi _k\rangle \right| |m|^{\theta }y^{1/2-\epsilon }(r_k+1)^{-1/3+\epsilon }e^{-\pi |m|y}\nonumber \\&\quad +\sum _{r_k>2|m|y}\left| \langle \Delta \Psi ,\phi _k\rangle \right| |m|^{\theta }y^{1/2-\epsilon }r_k^{-7/3+\epsilon }. \end{aligned}$$

Now using Cauchy–Schwarz followed by summation by parts (together with Weyl’s law stating that $\#\{r_k:r_k\le M\}\ll M^2$ (see e.g. [15, Corollary 11.2]) we can bound

$$\begin{aligned} \sum _{0\le r_k\le 2|m|y}\left| \langle \Psi , \phi _k\rangle \right| (r_k+1)^{-1/3+\epsilon }&\le \Vert \Psi \Vert _2\left( \sum _{0\le r_k\le 2|m|y}\frac{1}{(r_k+1)^{2/3-2\epsilon }}\right) ^{1/2}\\&\ll _{\epsilon } \Vert \Psi \Vert _2\left( |m|y\right) ^{2/3+\epsilon }. \end{aligned}$$

Similarly, for the second sum we can bound

$$\begin{aligned}&\sum _{r_k>2|m|y}\left| \langle \Delta \Psi ,\phi _k\rangle \right| r_k^{-7/3+\epsilon }\\&\quad \le \Vert \Delta \Psi \Vert _2\left( \sum _{r_k>2|m|y}r_k^{-14/3+2\epsilon }\right) ^{1/2}\ll _{\epsilon }\Vert \Delta \Psi \Vert _2\left( |m|y\right) ^{-4/3+\epsilon }. \end{aligned}$$

To summarize, the left-hand side of (3.9) is bounded by

3.10 $$\begin{aligned} \ll _\epsilon \ \Vert \Psi \Vert _2 |m|^{2/3+\theta +\epsilon } y^{7/6} e^{-\pi |m|y} + \Vert \Delta \Psi \Vert _2 |m|^{-4/3+\theta +\epsilon } y^{-5/6}. \end{aligned}$$

For the contribution from the continuous spectrum using the estimates (3.3), (3.8), the relation $\langle \Delta \Psi ,E(\cdot , \tfrac{1}{2}+ir)\rangle =(\tfrac{1}{4}+r^2)\langle \Psi , E(\cdot ,\tfrac{1}{2}+ir)\rangle$ and Cauchy–Schwarz we can similarly bound $\left| \int _{-\infty }^{\infty }\langle \Psi , E(\cdot , \tfrac{1}{2}+ir)\rangle a(\tfrac{1}{2}+ir;m,y)dr\right|$ by

$$\begin{aligned}&\ll _{\epsilon }e^{-\pi |m|y}y^{1/2-\epsilon }\int _{|r|\le 2|m|y}\left| \langle \Psi , E\left( \cdot , \tfrac{1}{2}+ir\right) \rangle \right| (|r|+1)^{-1/3+\epsilon }dr\\&\quad +y^{1/2-\epsilon }\int _{|r|>2|m|y}\left| \langle \Delta \Psi , E\left( \cdot , \tfrac{1}{2}+ir\right) \rangle \right| |r|^{-7/3+\epsilon }dr\\&\ll _{\epsilon }y^{1/2-\epsilon }\left( \Vert \Psi \Vert _2\left( |m|y\right) ^{1/6+\epsilon }e^{-\pi |m|y}+\Vert \Delta \Psi \Vert _2\left( |m|y\right) ^{-11/6+\epsilon }\right) , \end{aligned}$$

which is subsumed by the right-hand side of (3.10) (since $|m|y>1$). Finally, we conclude the proof by applying the bounds $\max \{\Vert \Psi \Vert _2, \Vert \Delta \Psi \Vert _2\}\le {\mathcal {S}}_{2,2}(\Psi )$ and $e^{-\pi |m|y}\ll (|m|y)^{-2}$ (again since $|m|y>1$) to the right hand side of (3.10). $\square$

The following corollary of Proposition 3.3 is the key estimate that we will use to prove Theorem 1.1.

#### Corollary 3.4

Let *q* be a positive integer. For any $\Psi \in C_c^{\infty }({\mathcal {M}})$, $y>0$, and any $\epsilon >0$, we have

$$\begin{aligned} \sum _{m\ne 0}\left| a_{\Psi }(qm,y)\right| \ll _{\epsilon }{\mathcal {S}}_{2,2}(\Psi )q^{-1}y^{-(1/2+\theta +\epsilon )}. \end{aligned}$$

#### Proof

If $qy\le 1$ we can separate the above sum into two parts to get

$$\begin{aligned} \sum _{m\ne 0}\left| a_{\Psi }(qm,y)\right|&=\sum _{1\le |m|\le (qy)^{-1}}\left| a_{\Psi }(qm,y)\right| +\sum _{|m|>(qy)^{-1}}\left| a_{\Psi }(qm,y)\right| . \end{aligned}$$

Applying (3.6) (and the estimate ${\mathcal {S}}_{\alpha _0}(\Psi )\le {\mathcal {S}}_{2,2}(\Psi )$ by Remark 3.7) to the first sum and Proposition 3.3 to the second, we have

$$\begin{aligned}&\sum _{m\ne 0}\left| a_{\Psi }(qm,y)\right| \\&\quad \ll _{\epsilon }{\mathcal {S}}_{2,2}(\Psi )\left( \sum _{1\le |m|\le (ny)^{-1}}|qm|^{\theta }y^{1/2-\epsilon }+\sum _{|m|>(qy)^{-1}}|qm|^{-4/3+\theta +\epsilon }y^{-5/6}\right) \\&\quad \asymp {\mathcal {S}}_{2,2}(\Psi )\left( q^{\theta }y^{1/2-\epsilon }(qy)^{-(1+\theta )}+q^{-4/3+\theta +\epsilon }y^{-5/6}(qy)^{1/3-\theta -\epsilon }\right) \\&\quad ={\mathcal {S}}_{2,2}(\Psi )q^{-1}y^{-(1/2+\theta +\epsilon )}, \end{aligned}$$

where for the second estimate we used that $4/3-\theta -\epsilon >1$. If $qy> 1$ then we have $|qm|y>1$ for all $m\ne 0$. We can apply Proposition 3.3 to $a_{\Psi }(qm,y)$ for all integers $m\ne 0$ to get

$$\begin{aligned} \sum _{m\ne 0}\left| a_{\Psi }(qm,y)\right|&\ll _{\epsilon } {\mathcal {S}}_{2,2}(\Psi )\sum _{|m|\ne 0}|qm|^{-4/3+\theta +\epsilon }y^{-5/6}\\&\ll {\mathcal {S}}_{2,2}(\Psi ) q^{-4/3+\theta +\epsilon } y^{-5/6}\ll {\mathcal {S}}_{2,2}(\Psi )q^{-1}y^{-(1/2+\theta +\epsilon )}, \end{aligned}$$

where for the last estimate we used that $\theta <1/3-\epsilon$. $\square$

#### Remark 3.11

The estimates in [18] hold more generally for any $\Gamma$ conjugate to some $\Gamma _0(p)$. In this generality, there might be (finitely many) exceptional cusp forms with $r_k\in i(0,\theta ]$. For such forms, it was shown in [18, Lemma 3.7] that for any $m\ne 0$

$$\begin{aligned} \left| a_{\phi _k}(m,y)\right| \ll _{\epsilon ,p}\Vert \Psi \Vert _2|m|^{\theta }y^{1/2-\epsilon }(|m|y)^{-|r_k|+\epsilon }e^{-2\pi |m|y}. \end{aligned}$$

Using the estimates $(|m|y)^{-|r_k|+\epsilon }e^{-2\pi |m|y}< (|m|y)^{-\theta }$ when $|m|y\le 1$ and $(|m|y)^{-|r_k|+\epsilon }e^{-2\pi |m|y}\ll (|m|y)^{-2}$ when $|m|y>1$ one can easily recover Corollary 3.4 for $\phi _k$, and hence for a general $\Psi \in C_c^{\infty }(\Gamma _0(p)\backslash {\mathbb {H}})$. Then one can easily deduce analogous estimates as in Theorem 1.1 for $\Psi$, see the arguments in the next subsection.

### Proof of Theorem 1.1

In this subsection we prove Theorem 1.1. In view of (3.5) it suffices to prove the following proposition.

#### Proposition 3.5

Let ${\mathcal {M}}$ be the modular surface. For any $\Psi \in C_c^\infty ({\mathcal {M}})$, for any $x\in {\mathbb {R}}/{\mathbb {Z}}$ and $y>0$, we have

3.12 $$\begin{aligned} \delta _{n,x,y}(\Psi )=a_{\Psi }(0,y)+O_{\epsilon }\left( {\mathcal {S}}_{2,2}(\Psi )n^{-1}y^{-(1/2+\theta +\epsilon )}\right) \end{aligned}$$

and

3.13 $$\begin{aligned} \delta ^{\mathrm{pr}}_{n,x,y}(\Psi )=a_{\Psi }(0,y)+O_{\epsilon }\left( {\mathcal {S}}_{2,2}(\Psi )n^{-1+\epsilon }y^{-(1/2+\theta +\epsilon )}\right) . \end{aligned}$$

#### Proof

Let $J\subset {\mathbb {R}}/{\mathbb {Z}}\cong [0,1)$ be a finite subset and for any $m\in {\mathbb {Z}}$ denote by $W_J(m):=\frac{1}{|J|}\sum _{t\in J}e(mt)$. We note that $\frac{1}{|J|}\sum _{t\in J}\Psi (t+iy)$ equals $\delta _{n,x,y}(\Psi )$ when $J=\{x+j/n :0\le j\le n-1\}$ and equals $\delta _{n,x,y}^{\mathrm{pr}}(\Psi )$ when $J=\left\{ x+j/n:0\le j\le n-1, \gcd (j,n)=1\right\}$. Applying the Fourier expansion (3.1) to $\Psi$ we get that

$$\begin{aligned} \frac{1}{|J|}\sum _{t\in J}\Psi (t+iy)&=\frac{1}{|J|}\sum _{t\in J}\sum _{m\in {\mathbb {Z}}}a_{\Psi }(m,y)e(mt)=\sum _{m\in {\mathbb {Z}}}a_{\Psi }(m,y)\frac{1}{|J|}\sum _{t\in J}e(mt)\\&=a_{\Psi }(0,y)+\sum _{m\ne 0}a_{\Psi }(m,y)W_J(m). \end{aligned}$$

Now for (3.12) we take $J=\{x+j/n:0\le j\le n-1\}$ and note that for such *J*, $|W_{J}(m)|$ equals 1 if $n\mid m$ and equals 0 otherwise. Hence

$$\begin{aligned} \left| \sum _{m\ne 0}a_{\Psi }(m,y)W_J(m)\right| \ \le \ \sum _{\begin{array}{c} m\ne 0\\ n | m \end{array}}|a_{\Psi }(m,y)|\ \ll _{\epsilon }\ n^{-1}y^{-(1/2+\theta +\epsilon )}, \end{aligned}$$

where for the last estimate we applied Corollary 3.4.

For (3.13) we take $J=\left\{ x+j/n:0\le j\le n-1, \gcd (j,n)=1\right\}$ and note the identity

$$\begin{aligned} \sum _{j\in ({\mathbb {Z}}/n{\mathbb {Z}})^{\times }}e\left( \tfrac{mj}{n}\right) =\frac{\mu (n_m)\varphi (n)}{\varphi (n_m)} \end{aligned}$$

for the Ramanujan’s sum, where $n_m:=n/\gcd (n,m)$ and $\mu : {\mathbb {N}}\rightarrow \{0, \pm 1\}$ is the Möbius function; see e.g. [14, Theorem 272]. Then

$$\begin{aligned} |W_J(m)|\ =\ \left| \frac{1}{\varphi (n)}\sum _{j\in ({\mathbb {Z}}/n{\mathbb {Z}})^{\times }}e\left( \tfrac{mj}{n}\right) \right| \ =\ \frac{|\mu (n_m)|}{\varphi \left( n_m\right) }\ \le \ \frac{1}{\varphi (n_m)}. \end{aligned}$$

Hence we have

$$\begin{aligned} \left| \sum _{m\ne 0}a_{\Psi }(m,y)W_J(m)\right| \,&\le \ \sum _{m\ne 0}\frac{\left| a_{\Psi }(m,y)\right| }{\varphi (n_m)}=\sum _{d| n}\frac{1}{\varphi (d)}\sum _{\begin{array}{c} m\ne 0\\ \gcd (m,n)=n/d \end{array}}\left| a_{\Psi }(m,y)\right| \\&\le \ \sum _{d| n}\frac{1}{\varphi (d)} \sum _{\begin{array}{c} m\ne 0\\ (n/d) | m \end{array}}\left| a_{\Psi }(m,y)\right| \\&\ll _{\epsilon }\sum _{d\mid n}\frac{1}{\varphi (d)}\left( \frac{n}{d}\right) ^{-1}y^{-(1/2+\theta +\epsilon )}\\&\ll _{\epsilon }\ n^{-1}\sigma _{\epsilon /2}(n)y^{-(1/2+\theta +\epsilon )}\ll _{\epsilon }n^{-1+\epsilon }y^{-(1/2+\theta +\epsilon )}, \end{aligned}$$

where for the second inequality we used the fact that $\gcd (m,n)=n/d$ implies that $(n/d)\mid m$, for the third inequality we applied Corollary 3.4 and for the second last inequality we applied the estimate $\varphi (d)\gg _{\epsilon } d^{1-\epsilon /2}$. $\square$

### Full range equidistribution for rational translates

In this subsection we prove Theorem 1.2. We fix $x=p/q$ a primitive rational number and let

$$\begin{aligned} {\mathbb {N}}_q=\left\{ n\in {\mathbb {N}}: \gcd (n^2, q)\mid n\right\} \end{aligned}$$

be as in Theorem 1.2. As mentioned in the introduction, the key ingredient is a symmetry lemma for rational translates which generalizes the symmetry (1.6). Before stating the lemma, let us briefly explain why we need to restrict to the subsequence ${\mathbb {N}}_q$. Let $n\in {\mathbb {N}}$ and let $y>0$. We need to study the distribution of the points $\Gamma (x+\tfrac{j}{n}+iy)=\Gamma (\tfrac{p}{q}+\tfrac{j}{n}+iy)$ for $0\le j\le n-1$. Let $\tfrac{p_j}{q_j}$ be the reduced form of $\tfrac{p}{q}+\tfrac{j}{n}$ and in view of the symmetry (1.6) we have

$$\begin{aligned} \Gamma \left( x+\tfrac{j}{n}+iy\right) =\Gamma \left( \tfrac{p_j}{q_j}+iy\right) =\Gamma \left( -\tfrac{\overline{p_j}}{q_j}+\tfrac{i}{q_j^2y}\right) , \end{aligned}$$

where $\overline{p_j}$ is the multiplicative inverse of $p_j$ modulo $q_j$. To further analyze the distribution of these points, we thus need to solve the congruence equation $xp_j\equiv 1\ (\mathrm {mod}\ q_j)$ in *x*. Write $k=\gcd (n, q)$ and $q'=q/k$ and $n'=n/k$. Then

$$\begin{aligned} \tfrac{p}{q}+\tfrac{j}{n}=\tfrac{p}{kq'}+\tfrac{j}{kn'}=\tfrac{pn'+jq'}{kq'n'}, \end{aligned}$$

implying that

$$\begin{aligned} q_j=\tfrac{kq'n'}{\gcd (pn'+jq', kq'n')}=\tfrac{kn'q'}{\gcd (pn'+jq', kn')}=q'\tfrac{n}{\gcd (pn'+jq',n)} \end{aligned}$$

can be written canonically as a product of two integers. Here for the second equality we used that $\gcd (pn'+jq',q')=\gcd (pn',q')=1$. In view of the Chinese remainder theorem, the above congruence equation modulo $q_j$ is relatively easy to solve when the two factors $q'$ and $n/\gcd (pn'+jq', n)$ are coprime (see the proof of Lemma 3.6 for more details). This condition can be guaranteed for any *j* if $\gcd (q', n)=\gcd (q/\gcd (q,n),n)=1$ which is equivalent to the condition $n\in {\mathbb {N}}_q$. Finally, we also note that by writing *n* and *q* in prime decomposition forms, it is not hard to check that $n\in {\mathbb {N}}_q$ is equivalent to $q=kl$ with $l=\gcd (n,q)\mid n$ and $\gcd (k,n)=1$. We now state the symmetry lemma.

#### Lemma 3.6

Let $\tfrac{m}{kl}$ be a primitive rational number and let $n\in {\mathbb {N}}$ such that $l\mid n$ and $\gcd (k,n)=1$. Then for any $0\le j\le n-1$ and for any $y>0$ we have

3.14 $$\begin{aligned} \Gamma \left( \tfrac{m}{kl}+\tfrac{j}{n}+iy\right) =\Gamma \left( -\tfrac{dl{\overline{mn}}a}{k}-\tfrac{\left( \left( m\tfrac{n}{l}+jk\right) /d\right) ^*b}{n/d}+i\tfrac{d^2}{k^2n^2y}\right) , \end{aligned}$$

where $d=d_{j}:=\gcd (m\tfrac{n}{l}+jk, n)$ and $a=a_d$, $b=b_d\in {\mathbb {Z}}$ are some fixed integers such that $a\tfrac{n}{d}+bk=1$. Here, for any integer *x*, ${\overline{x}}$ denotes the multiplicative inverse of *x* modulo *k*, $x^*$ denotes the multiplicative inverse of *x* modulo *n*/*d*. If we further assume $\gcd (j,n)=l=1$, then $d_j=\gcd (mn+jk, n)=1$ and

3.15 $$\begin{aligned} \Gamma \left( \tfrac{m}{k}+\tfrac{j}{n}+iy\right) =\Gamma \left( -\tfrac{{\overline{mn}}a}{k}-\tfrac{(jk)^*b}{n}+\tfrac{i}{k^2n^2y}\right) . \end{aligned}$$

#### Proof

Since $l\mid n$, by direct computation we have $\frac{m}{kl}+\frac{j}{n}=\frac{mn/l+jk}{kn}$. Note that since $\gcd (k, mn)=1$ we have $\gcd (m\tfrac{n}{l}+jk, k)=\gcd (m\tfrac{n}{l}, k)=1$. This implies that $\gcd (m\tfrac{n}{l}+jk, kn)=\gcd (m\tfrac{n}{l}+jk, n)=d$. Hence let $\tfrac{p}{q}$ be the reduced form of $\tfrac{m}{kl}+\tfrac{j}{n}$, then we have $(p,q)=((m\tfrac{n}{l}+jk)/d, kn/d)$. Now since $\gcd (p,q)=1$, there exist some integers $v, w\in {\mathbb {Z}}$ such that $$\gamma =\left( {\begin{matrix} w &{} v\\ -q &{} p\end{matrix}}\right) \in \Gamma $$. By direct computation we have

$$\begin{aligned} \gamma \left( \tfrac{m}{kl}+\tfrac{j}{n}+iy\right) =\gamma \left( \tfrac{p}{q}+iy\right) =-\tfrac{w}{q}+\tfrac{i}{q^2y}. \end{aligned}$$

implying that

3.16 $$\begin{aligned} \Gamma \left( \tfrac{m}{kl}+\tfrac{j}{n}+iy\right) =\Gamma \left( -\tfrac{w}{q}+\tfrac{i}{q^2y}\right) =\Gamma \left( -\tfrac{w}{kn/d}+i\tfrac{d^2}{k^2n^2y}\right) , \end{aligned}$$

where for the second equality we used the relation $q=kn/d$. Moreover, since $\gamma \in \Gamma$ we have $wp+vq=1$, implying that (again using the relation $(p,q)=((m\tfrac{n}{l}+jk)/d, kn/d)$)

$$\begin{aligned} w\left( (m\tfrac{n}{l}+jk)/d\right) \equiv 1\ (\mathrm {mod}\ k\tfrac{n}{d}). \end{aligned}$$

We claim that

3.17 $$\begin{aligned} w\equiv dl{\overline{mn}}\tfrac{n}{d}a+\left( \left( m\tfrac{n}{l}+jk\right) /d\right) ^*kb \ (\mathrm {mod}\ k\tfrac{n}{d}). \end{aligned}$$

In view of the Chinese Remainder Theorem, since $\gcd (k, n/d)=1$, it suffices to check

$$\begin{aligned} \left( dl{\overline{mn}}\tfrac{n}{d}a+\left( \left( m\tfrac{n}{l}+jk\right) /d\right) ^*kb\right) \left( (m\tfrac{n}{l}+jk)/d\right) \equiv 1\ (\mathrm {mod}\ k) \end{aligned}$$

and

$$\begin{aligned} \left( dl{\overline{mn}}\tfrac{n}{d}a+\left( \left( m\tfrac{n}{l}+jk\right) /d\right) ^*kb\right) \left( (m\tfrac{n}{l}+jk)/d\right) \equiv 1\ (\mathrm {mod}\ \tfrac{n}{d}). \end{aligned}$$

For the first equation we have

$$\begin{aligned}&\left( dl{\overline{mn}}\tfrac{n}{d}a+\left( \left( m\tfrac{n}{l}+jk\right) /d\right) ^*kb\right) \left( (m\tfrac{n}{l}+jk)/d\right) \equiv dl{\overline{mn}}\tfrac{n}{d}a mn{\overline{ld}}\equiv a\tfrac{n}{d}\\&\quad =1-bk\equiv 1\ (\mathrm {mod}\ k), \end{aligned}$$

where for the first equality we used the fact that $\gcd (dl, k)=1$ (since $d\mid n$, $l\mid n$ and $\gcd (k,n)=1$). The second equation follows similarly. Now plugging relation (3.17) into (3.16) we get (3.14).

For the second half we note that $d_j=\gcd (mn+jk, n)=\gcd (jk,n)=1$. The first equality is true since $l=1$, and the second equality is true since by assumption $\gcd (k,n)=\gcd (j,n)=1$. Thus in view of (3.14), to prove (3.15) it suffices to note that $(mn+jk)^*\equiv (jk)^*\ (\mathrm {mod}\ n)$, or equivalently, $mn+jk\equiv jk\ (\mathrm {mod}\ n)$. $\square$

#### Remark 3.18

When $k=1$ we can take $(a,b)=(0,1)$, then (3.15) recovers the symmetry (1.6). We also note that for the point $\Gamma (x+j/n+iy)$ with *x* irrational, the above symmetry clearly breaks.

#### Proposition 3.7

Let *p*/*q* be a primitive rational number and let $n\in {\mathbb {N}}_q$. Then for any $y>0$ we have

3.19 $$\begin{aligned} {\mathcal {R}}_n\left( \tfrac{p}{q},y\right) =\bigcup _{d| n}{\mathcal {R}}_{n/d}^{\mathrm{pr}}\left( x_{d}, \tfrac{d^2}{k^2n^2y}\right) , \end{aligned}$$

where $x_{d}\in {\mathbb {R}}/{\mathbb {Z}}$ is some number depending on *d* (and also on *p*, *q*, *n*) and $k:=q/\gcd (n, q)$. If we further assume $\gcd (n,q)=1$, then

3.20 $$\begin{aligned} {\mathcal {R}}_n^{\mathrm{pr}}\left( \tfrac{p}{q}, y\right) ={\mathcal {R}}_n^{\mathrm{pr}}\left( -\tfrac{{\overline{pn}}a}{q}, \tfrac{1}{q^2n^2y}\right) , \end{aligned}$$

where ${\overline{x}}$ denotes the multiplicative inverse of *x* modulo *q* and $a\in {\mathbb {Z}}$ is as in Lemma 3.6.

#### Proof

Relation (3.20) follows immediately from (3.15) by taking $(m,k)=(p,q)$ and noting that

$$\begin{aligned} \{(-[(qj)^*b]\in ({\mathbb {Z}}/n{\mathbb {Z}})^{\times }: j\in ({\mathbb {Z}}/n{\mathbb {Z}})^{\times }\}=({\mathbb {Z}}/n{\mathbb {Z}})^{\times }, \end{aligned}$$

which follows from the fact that $\gcd (bq,n)=1$ (since $\gcd (bq, n)=\gcd (1-an, n)=1$). Here $(qj)^*$ denotes the multiplicative inverse of *qj* modulo *n* and $b\in {\mathbb {Z}}$ is as in Lemma 3.6.

For (3.19), we set $m=p$, $l=\gcd (n, q)$ (so that $k=q/l$). As mentioned above, the condition $\gcd (n^2, q)\mid n$ implies that $\gcd (k,n)=1$. Thus the pair $(\tfrac{m}{kl}, n)$ satisfies the assumptions in Lemma 3.6 and we can apply (3.14) for the points

$$\begin{aligned} \Gamma \left( \tfrac{p}{q}+\tfrac{j}{n}+iy\right) =\Gamma \left( \tfrac{m}{kl}+\tfrac{j}{n}+iy\right) , 0\le j\le n-1. \end{aligned}$$

Now for any $d\mid n$ define

$$\begin{aligned} D_d:=\left\{ 0\le j\le n-1: d_{j}=\gcd (m\tfrac{n}{l}+jk, n)=d\right\} \end{aligned}$$

so that

3.21 $$\begin{aligned} {\mathcal {R}}_n\left( \tfrac{p}{q},y\right) =\bigcup _{d\mid n}\left\{ \Gamma \left( \tfrac{p}{q}+\tfrac{j}{n}+iy\right) \in {\mathcal {M}}: j\in D_d\right\} . \end{aligned}$$

Moreover, we note that since $\gcd (k,n)=1$, we have

$$\big \{[m\tfrac{n}{l}+jk]\in {\mathbb {Z}}/n{\mathbb {Z}}: 0\le j\le n-1\big \}={\mathbb {Z}}/n{\mathbb {Z}}$$

and hence

3.22 $$\begin{aligned} \left\{ [m\tfrac{n}{l}+jk]\in {\mathbb {Z}}/n{\mathbb {Z}}: j\in D_d\right\} =\left\{ [j]\in {\mathbb {Z}}/n{\mathbb {Z}}: \gcd (j, n)=d\right\} . \end{aligned}$$

On the other hand, by (3.14) we have

$$\begin{aligned}&\left\{ \Gamma \left( \tfrac{p}{q}+\tfrac{j}{n}+iy\right) \in {\mathcal {M}}: j\in D_d\right\} \\&\quad =\left\{ \Gamma \left( -\tfrac{dl{\overline{mn}}a_d}{k}-\tfrac{\left( \left( m\tfrac{n}{l}+jk\right) /d\right) ^*b_d}{n/d}+i\tfrac{d^2}{k^2n^2y}\right) \in {\mathcal {M}}: j\in D_d \right\} , \end{aligned}$$

where for any integer *x*, ${\overline{x}}$ denotes the multiplicative inverse of *x* modulo *k*, $x^*$ denotes the multiplicative inverse of *x* modulo *n*/*d*, and $a_d, b_d\in {\mathbb {Z}}$ are some fixed integers such that $a_d\tfrac{n}{d}+b_dk=1$. Now for each $d\mid n$ we let $x_d\in [0,1)$, $x_d\equiv -\tfrac{dl{\overline{mn}}a_d}{k} \ (\mathrm {mod}\ 1)$ so that it remains to show

$$\begin{aligned} \left\{ -[\left( (m\tfrac{n}{l}+jk)/d\right) ^*b_d]\in ({\mathbb {Z}}/(n/d){\mathbb {Z}})^{\times }: j\in D_d\right\} =({\mathbb {Z}}/(n/d){\mathbb {Z}})^{\times }. \end{aligned}$$

We can thus conclude the proof by noting that the above relation follows immediately from (3.22) together with the fact $\gcd (b_d,\tfrac{n}{d})=1$ (since $\gcd (b_d,\tfrac{n}{d})=\gcd (b_dk, \tfrac{n}{d})=\gcd (1-a_d\tfrac{n}{d}, \tfrac{n}{d})=1)$. $\square$

Using these two relations and the estimate (3.13) one gets the following effective estimates.

#### Proposition 3.8

Let $x=p/q$ be a primitive rational number and let $n\in {\mathbb {N}}_q$. Then for any $\Psi \in C_c^{\infty }({\mathcal {M}})$ and $y>0$ we have

$$\begin{aligned} \delta _{n,x,y}(\Psi )=\frac{1}{n}\sum _{d\mid n}\varphi \left( \tfrac{n}{d}\right) a_{\Psi }\left( 0, \tfrac{d^2}{k^2n^2y}\right) +O_{\epsilon ,q}\left( {\mathcal {S}}_{2,2}(\Psi )n^{2\theta +4\epsilon }y^{1/2+\theta +\epsilon }\right) , \end{aligned}$$

where $k:=q/\gcd (n, q)$. If we further assume that $\gcd (n,q)=1$, then

$$\begin{aligned} \delta _{n,x,y}^{\mathrm{pr}}(\Psi )=a_{\Psi }\left( 0, \tfrac{1}{q^2n^2y}\right) +O_{\epsilon ,q}\left( {\mathcal {S}}_{2,2}(\Psi )n^{2\theta +3\epsilon }y^{1/2+\theta +\epsilon }\right) . \end{aligned}$$

#### Proof

For any positive divisor $d\mid n$, let $y_d=d^2/(k^2n^2y)$ with $k:=q/\gcd (n, q)$ as above and let $x_d\in {\mathbb {R}}/{\mathbb {Z}}$ be as in (3.19). Then by (3.19) for $x=p/q$ we have

$$\begin{aligned} \delta _{n,x,y}(\Psi )&=\frac{1}{n}\sum _{d| n}\varphi \left( \tfrac{n}{d}\right) \delta ^{\mathrm{pr}}_{n/d, x_d, y_d}(\Psi )\\&=\frac{1}{n}\sum _{d\mid n}\varphi \left( \tfrac{n}{d}\right) \left( a_{\Psi }\left( 0, y_d\right) +O_{\epsilon }\left( {\mathcal {S}}_{2,2}(\Psi )\left( \tfrac{n}{d}\right) ^{-1+\epsilon }y_d^{-(1/2+\theta +\epsilon )}\right) \right) \\&=\frac{1}{n}\sum _{d\mid n}\varphi \left( \tfrac{n}{d}\right) a_{\Psi }\left( 0, y_d\right) +O_{\epsilon }\left( {\mathcal {S}}_{2,2}(\Psi )n^{-1}\sum _{d\mid n}\left( \tfrac{n}{d}\right) ^{\epsilon }y_d^{-(1/2+\theta +\epsilon )}\right) , \end{aligned}$$

where for the second estimate we applied (3.13) and for the third estimate we used the trivial estimate $\varphi (n/d)<n/d$. Now plugging $y_d=d^2/(k^2n^2y)$ into the above equation we get

$$\begin{aligned} \delta _{n,x,y}(\Psi )&=\frac{1}{n}\sum _{d\mid n}\varphi \left( \tfrac{n}{d}\right) a_{\Psi }\left( 0, \tfrac{d^2}{k^2n^2y}\right) +O_{\epsilon ,q}\left( {\mathcal {S}}_{2,2}(\Psi )n^{-1}\sigma _{1+2\theta +3\epsilon }(n)y^{1/2+\theta +\epsilon }\right) \\&=\frac{1}{n}\sum _{d\mid n}\varphi \left( \tfrac{n}{d}\right) a_{\Psi }\left( 0, \tfrac{d^2}{k^2n^2y}\right) +O_{\epsilon ,q}\left( {\mathcal {S}}_{2,2}(\Psi )n^{2\theta +4\epsilon }y^{1/2+\theta +\epsilon }\right) , \end{aligned}$$

where the dependence on *k* in the first estimate is absorbed into the dependence on *q* (since ${{k:=q/\gcd (n, q)}}\le q$). The second estimate follows from similar (but easier) analysis with the relation (3.20) in place of (3.19). $\square$

We are now in the position to prove Theorem 1.2. We will prove the following proposition from which Theorem 1.2 follows, see also Remark 3.23.

#### Theorem 3.9

Let $x=p/q$ be a primitive rational number and let $n\in {\mathbb {N}}_q$. Let $y_n=c/n^{\alpha }$ for some $1<\alpha <2$ and $c>0$. Then for any $\Psi \in C_c^{\infty }({\mathcal {M}})$ we have

$$\begin{aligned} \left| \delta _{n,x,y_n}(\Psi )-\mu _{{\mathcal {M}}}(\Psi )\right| \ll _{\epsilon ,q, c,\Psi }n^{\alpha /2-1+\epsilon }+n^{2\theta +4\epsilon -\alpha (1/2+\theta +\epsilon )}. \end{aligned}$$

If we further assume $\gcd (n,q)=1$, then we have

$$\begin{aligned} \left| \delta ^{\mathrm{pr}}_{n,x,y_n}(\Psi )-\mu _{{\mathcal {M}}}(\Psi )\right| \ll _{\epsilon , q, c}{\mathcal {S}}_{2,2}(\Psi )\left( n^{\alpha /2-1}+n^{2\theta +3\epsilon -\alpha (1/2+\theta +\epsilon )}\right) . \end{aligned}$$

#### Remark 3.23

The dependence on $\Psi$ in the first estimate can also be made explicit. In fact, we can remove this dependence by adding a factor of ${\mathcal {S}}_{2,2}(\Psi )+\Vert \Psi \Vert _{\infty }$ to the right hand side of this estimate. We also note that since we may take $\theta =7/64$, the right hand side of these two estimates decays to zero as $n\rightarrow \infty$ for any $1<\alpha <2$.

#### Proof

In view of Proposition 3.8 and the assumption $y_n=c/n^{\alpha }$, it suffices to show that

$$\begin{aligned} \frac{1}{n}\sum _{d\mid n}\varphi \left( \tfrac{n}{d}\right) a_{\Psi }\left( 0, \tfrac{d^2}{k^2n^2y_n}\right) =\mu _{{\mathcal {M}}}(\Psi )+O_{\epsilon , c,\Psi }\left( n^{\alpha /2-1+\epsilon }\right) \end{aligned}$$

with $k:=q/\gcd (n, q)$, and that (under the extra assumption $\gcd (n,q)=1$)

$$\begin{aligned} a_{\Psi }\left( 0, \tfrac{1}{q^2n^2y_n}\right) =\mu _{{\mathcal {M}}}(\Psi )+O_{c}\left( {\mathcal {S}}_{2,2}(\Psi )n^{\alpha /2-1}\right) . \end{aligned}$$

The second estimate follows immediately from (3.5) and the trivial estimate $|q|\ge 1$. For the first estimate we separate the sum into two parts to get

$$\begin{aligned} \frac{1}{n}\sum _{d\mid n}\varphi \left( \tfrac{n}{d}\right) a_{\Psi }\left( 0, \tfrac{d^2}{k^2n^2y_n}\right)&=\frac{1}{n}\left( \sum _{\begin{array}{c} d\mid n\\ d< n^{1-\alpha /2} \end{array}}+\sum _{\begin{array}{c} d| n\\ d\ge n^{1-\alpha /2} \end{array}}\right) \varphi \left( \tfrac{n}{d}\right) a_{\Psi }\left( 0, \tfrac{d^2}{k^2n^2y_n}\right) . \end{aligned}$$

Applying (3.5) (and the trivial estimate $|k|\ge 1$) for the first sum and applying the estimate

$$\begin{aligned} \left| a_{\Psi }\left( 0, \tfrac{d^2}{k^2n^2y_n}\right) \right| =\left| \int _0^1\Psi \left( t+i\tfrac{d^2}{k^2n^2y_n}\right) dt\right| \le \Vert \Psi \Vert _{\infty } \end{aligned}$$

for the second sum we get $\frac{1}{n}\sum _{d\mid n}\varphi \left( \tfrac{n}{d}\right) a_{\Psi }\left( 0, \tfrac{d^2}{k^2n^2y_n}\right)$ equals

$$\begin{aligned}&\frac{1}{n}\left( \sum _{\begin{array}{c} d\mid n\\ d< n^{1-\alpha /2} \end{array}}\varphi \left( \tfrac{n}{d}\right) \left( \mu _{{\mathcal {M}}}(\Psi )+O_{c,\Psi }\left( \left( \tfrac{n}{d}\right) ^{-1}n^{\alpha /2}\right) \right) +O_{\Psi }\left( \sum _{\begin{array}{c} d| n\\ d\ge n^{1-\alpha /2} \end{array}}\varphi \left( \tfrac{n}{d}\right) \right) \right) \\&\quad =\mu _{{\mathcal {M}}}(\Psi )+\frac{1}{n}O_{c, \Psi }\left( n^{\alpha /2}\sum _{\begin{array}{c} d\mid n\\ d< n^{1-\alpha /2} \end{array}}1+\sum _{\begin{array}{c} d| n\\ d\ge n^{1-\alpha /2} \end{array}}\tfrac{n}{d}\right) \\&\quad =\mu _{{\mathcal {M}}}(\Psi )+O_{c, \Psi }\left( n^{\alpha /2-1}\sigma _0(n)\right) =\mu _{{\mathcal {M}}}(\Psi )+O_{\epsilon , c, \Psi }\left( n^{\alpha /2-1+\epsilon }\right) , \end{aligned}$$

finishing the proof, where for the first estimate we used the identity that $\sum _{d\mid n}\varphi (n/d)=n$ and the estimate that $\varphi \left( n/d\right) <n/d$, and for the second estimate we used the estimates $$\sum _{\begin{array}{c} d\mid n\\ d< n^{1-\alpha /2} \end{array}}1\le \sigma _{0}(n)$$ and

$$\begin{aligned} \sum _{\begin{array}{c} d| n\\ d\ge n^{1-\alpha /2} \end{array}}\frac{n}{d}=\sum _{\begin{array}{c} d\mid n\\ d\le n^{\alpha /2} \end{array}}d\le n^{\alpha /2}\sum _{\begin{array}{c} d\mid n\\ d\le n^{\alpha /2} \end{array}}1\le n^{\alpha /2}\sigma _0(n). \end{aligned}$$

$$\square $$

### Quantitative non-equidistribution for rational translates

As a direct consequence of the analysis in the previous subsection we also have the following quantitative non-equidistribution result for rational translates when $\{y_n\}_{n\in {\mathbb {N}}}$ is beyond the above range, generalizing the situation for $\{{\mathcal {R}}_n^{\mathrm{pr}}(0,y_n)\}_{n\in {\mathbb {N}}}$. As before, for any $Y>0$ we denote by $\mu _Y$ the probability uniform distribution measure supported on ${\mathcal {H}}_Y$.

#### Theorem 3.10

Let $x=p/q$ be a primitive rational number and let $y_n=c/n^2$ for some constant $c>0$. Let $\Psi \in C_c^{\infty }(\Psi )$. Then for any $n\in {\mathbb {N}}_q$ we have

$$\begin{aligned} \delta _{n,x,y_n}(\Psi )=\frac{1}{n}\sum _{d\mid n}\varphi \left( \tfrac{n}{d}\right) \mu _{\tfrac{d^2}{ck_n^2}}(\Psi )+O_{\epsilon ,q, c}\left( {\mathcal {S}}_{2,2}(\Psi )n^{-1+2\epsilon }\right) \end{aligned}$$

with $k_n=q/\gcd (n^2, q)$. If we further assume that $\gcd (n,q)=1$, then

$$\begin{aligned} \delta _{n,x,y_n}^{\mathrm{pr}}(\Psi )=\mu _{\tfrac{1}{cq^2}}(\Psi )+O_{\epsilon ,q,c}\left( {\mathcal {S}}_{2,2}(\Psi )n^{-1+\epsilon }\right) . \end{aligned}$$

#### Proof

These two effective estimates follow immediately from Proposition 3.8 by plugging in $y_n=c/n^2$ and noting that $a_{\Psi }(0, Y)=\int _0^1\Psi (x+iY)dx=\mu _Y(\Psi )$. $\square$

We can now give the

#### Proof

For part (1), in view of Theorem 3.10 only the second equation needs a proof. Since we are taking $n\in {\mathbb {P}}_m$ going to infinity, it is sufficient to consider $n=m\ell \in {\mathbb {P}}_m$ with the prime number $\ell >q$ (so that $\ell \not \mid q$). For such *n*, we have $\gcd (n^2, q)=\gcd (m^2\ell ^2, q)=\gcd (m^2, q)$. Since by assumption $\gcd (m^2, q)\mid m$ and $m\mid n$, we can apply the first effective estimate in Theorem 3.10 for such $n=m\ell \in {\mathbb {P}}_m$. Moreover, for any such *n* we have

$$\begin{aligned} k_n=\frac{q}{\gcd (n^2, q)}=\frac{q}{\gcd (m^2, q)}=\frac{q}{\gcd (m,q)} \end{aligned}$$

is a fixed number only depending on *m* and *q*. Here for the last equality we used the assumption that $\gcd (m^2, q)\mid m$. Now let $n=m\ell \in {\mathbb {P}}_{m}$ with $\ell \gg q$ sufficiently large such that $\mu _{Y}(\Psi )=0$ whenever $Y>\ell ^2/(ck_n)^2$ (this can be guaranteed since $k_n$ is a fixed number and $\Psi$ is compactly supported). In particular, for any $d\mid n$, $\mu _{d^2/(ck_n^2)}(\Psi )=0$ whenever $\ell \mid d$. This, together with the first estimate in Theorem 3.10 implies that for all such sufficiently large $n=m\ell \in {\mathbb {P}}_{m}$

$$\begin{aligned} \delta _{n,x,y_n}(\Psi )&=\frac{1}{m\ell }\sum _{d\mid m}\varphi \left( \tfrac{m\ell }{d}\right) \mu _{\tfrac{d^2}{ck_n^2}}(\Psi )+O_{\epsilon ,q,c,\Psi ,m}\left( \ell ^{-1+2\epsilon }\right) \\&=\frac{\ell -1}{\ell }\nu _{m,\tfrac{1}{ck_n^2}}(\Psi )+O_{\epsilon ,q,c,\Psi ,m}\left( \ell ^{-1+2\epsilon }\right) , \end{aligned}$$

where for the second estimate we used that $\gcd (m,\ell )=1$ and $\ell$ is a prime number. We can now finish the proof by taking $n=m\ell \rightarrow \infty$ along the subsequence ${\mathbb {P}}_{m}$ (equivalently, taking $\ell \rightarrow \infty$) and plugging in the relation $k_n=q/\gcd (m,q)$.

For part (2), since ${\mathcal {R}}_n^{\mathrm{pr}}(x,y_n)\subset {\mathcal {R}}_n(x,y_n)$, we only need to prove the full escape to the cusp for the sequence $\{{\mathcal {R}}_n(x,y_n)\}_{n\in {\mathbb {N}}}$. Identify (up to a null set) ${\mathcal {M}}$ with the standard fundamental domain ${\mathcal {F}}_{\Gamma }:=\left\{ z\in {\mathbb {H}}: {\mathfrak {Re}}(z)<\frac{1}{2}, |z|>1\right\}$. For any $n\in {\mathbb {N}}$ and $0\le j\le n-1$ let $\tfrac{p_j}{q_j}$ be the reduced form of $x+\tfrac{j}{n}=\tfrac{p}{q}+\tfrac{j}{n}=\tfrac{pn+qj}{qn}$ so that by (1.6)

$$\begin{aligned} \Gamma \left( x+\tfrac{j}{n}+iy_n\right) =\Gamma \left( -\tfrac{\overline{p_j}}{q_j}+\tfrac{i}{q_j^2y_n}\right) . \end{aligned}$$

Thus using the trivial inequality $|q_j|\le |q|n$ for all $0\le j\le n-1$ and the assumption $\lim \limits _{n\rightarrow \infty }n^2y_n=0$, we have

$$\begin{aligned} {\mathcal {R}}(x,y_n)\subset \left\{ z\in {\mathcal {F}}_{\Gamma }: {\mathfrak {Im}}(z)\ge \tfrac{1}{q^2n^2y_n}\right\} \xrightarrow {n\rightarrow \infty } \text {cusp of}\, {\mathcal {M}}. \end{aligned}$$

$$\square $$

## Negative results: in connection with Diophantine approximations

Let $\Gamma ={\text {SL}}_2({\mathbb {Z}})$ and ${\mathcal {M}}=\Gamma \backslash {\mathbb {H}}$ be the modular surface. Let $\mu _{{\mathcal {M}}}$ be the normalized hyperbolic area on ${\mathcal {M}}$ as before. In this section we prove a general result which captures the cusp excursion rate for the sample points ${\mathcal {R}}_n(x,y_n)$ in terms of the Diophantine property of the translate $x\in {\mathbb {R}}/{\mathbb {Z}}\cong [0,1)$, see Theorem 4.3. Theorem 1.4 will then be an easy consequence of this result.

### Notation and a preliminary result on cusp excursions

In this subsection we prove a preliminary lemma relating cusp excursions on the modular surface to Diophantine approximations. Let us first fix some notation. For any $Y>0$, we denote by ${\mathcal {C}}_Y\subset {\mathcal {M}}$ the image of the region

$$\begin{aligned} \{z\in {\mathbb {H}}:{\mathfrak {Im}}(z)>Y\} \end{aligned}$$

under the natural projection from ${\mathbb {H}}$ to ${\mathcal {M}}=\Gamma \backslash {\mathbb {H}}$. As *Y* goes to infinity, the sets ${\mathcal {C}}_Y$ diverge to the cusp of ${\mathcal {M}}$, and we call ${\mathcal {C}}_Y$ a *cusp neighborhood of* ${\mathcal {M}}$. Similarly, for any $Y'>Y>0$, we denote by ${\mathcal {C}}_{Y,Y'}$ the projection onto ${\mathcal {M}}$ of the open set

$$\begin{aligned} \left\{ z\in {\mathbb {H}}:Y<{\mathfrak {Im}}(z)< Y'\right\} . \end{aligned}$$

For any primitive rational number *m*/*n*, and for any $r>0$ we denote by

$$\begin{aligned} H_{m/n,r}:=\left\{ z=x+iy\in {\mathbb {H}}:(x-m/n)^2+(y-r)^2= r^2\right\} \end{aligned}$$

the horocycle tangent to $\partial {\mathbb {H}}$ at *m*/*n* with Euclidean radius *r*. We denote by

$$\begin{aligned} H^{\circ }_{m/n,r}:=\left\{ z=x+iy\in {\mathbb {H}}:(x-m/n)^2+(y-r)^2< r^2\right\} \end{aligned}$$

the open horodisc enclosed by $H_{m/n,r}$. We have the following geometric description of Lemma 3.6: Let $$\gamma =\left( {\begin{matrix} m &{} * \\ n &{} *\end{matrix}}\right) $$ be an element in $\Gamma$. Then $\gamma$ sends the horizontal horocycle $\{z\in {\mathbb {H}}:{\mathfrak {Im}}(z)=Y\}$ to the horocycle $H_{m/n,r}$ with $r=1/(2Yn^2)$, while the open region $\left\{ z\in {\mathbb {H}}:{\mathfrak {Im}}(z)>Y\right\}$ is mapped to the horodisc $H^{\circ }_{m/n,r}$. On the other hand, for any primitive rational number *m*/*n*, there is $\gamma \in \Gamma$ of the form $$\gamma =\left( {\begin{matrix} m&{}*\\ n&{}*\end{matrix}}\right) $$. Thus for any $Y>0$ and for any $z\in {\mathbb {H}}$, $\Gamma z\in {\mathcal {C}}_Y$ if and only if $z\in H^{\circ }_{m/n,r}$ for some primitive rational number *m*/*n* with $r=1/(2Yn^2)$.

Finally, we record a distance formula that we will later use. Let $d_{{\mathcal {M}}}(\cdot ,\cdot )$ be the distance function on ${\mathcal {M}}$ induced from the hyperbolic distance function $d_{{\mathbb {H}}}$ on ${\mathbb {H}}$, i.e.,

$$\begin{aligned} d_{{\mathcal {M}}}(\Gamma z_1, \Gamma z_2)=\inf _{\gamma \in \Gamma }d_{{\mathbb {H}}}(\gamma z_1, z_2). \end{aligned}$$

#### Lemma 4.1

Let $\Gamma z_0\in {\mathcal {M}}$ be a fixed base point. Then there exists a constant $c>0$ (which may depend on $\Gamma z_0)$ such that for any $Y>1$ and for any $\Gamma z\in {\mathcal {C}}_Y$

4.1 $$\begin{aligned} d_{{\mathcal {M}}}(\Gamma z_0,\Gamma z)\ge \log Y - c. \end{aligned}$$

The estimate (4.1) holds for a general non-compact finite-volume hyperbolic manifold using reduction theory after Garland and Raghunathan [11, Theorem 0.6] combined with a distance estimate by Borel [2, Theorem C]. We give here a self-contained elementary proof for the special case of the modular surface.

#### Proof

In view of the triangle inequality, we may assume $\Gamma z_0=\Gamma i$. Note that $d_{{\mathbb {H}}}(i, z)\ge \log Y$ for any $z\in {\mathbb {H}}$ with ${\mathfrak {Im}}(z)\in (0, 1/Y)\cup (Y,\infty )$. Thus it suffices to show that if $\Gamma z\in {\mathcal {C}}_Y$, then ${\mathfrak {Im}}(\gamma z)\in (0,1/Y)\cup (Y,\infty )$ for any $\gamma \in \Gamma$. By the definition of ${\mathcal {C}}_Y$, we may assume $z=x+iy\in {\mathbb {H}}$ with $y>Y$. Now let $$\gamma =\left( {\begin{matrix} * &{} *\\ a &{} b\end{matrix}}\right) \in \Gamma $$. If $a=0$, then ${\mathfrak {Im}}(\gamma z)={\mathfrak {Im}}(z)>Y$. If $a\ne 0$, then

$$\begin{aligned} {\mathfrak {Im}}(\gamma z)\ =\ \frac{{\mathfrak {Im}}(z)}{|az+b|^2}\ =\ \frac{y}{(ax+b)^2+a^2y^2}\ \le \ \frac{1}{y}\ <\ \frac{1}{Y}. \end{aligned}$$

$$\square $$

The following simple lemma is the key observation relating cusp excursions with Diophantine approximation.

#### Lemma 4.2

Let $x\in [0,1)$ be a real number. Suppose there exist a primitive rational number *m*/*n* and $n>0$, and a real number $Y>0$ satisfying

$$\begin{aligned} \left| x-\frac{m}{n}\right| <\frac{1}{2Yn^2}. \end{aligned}$$

Then for any $0\le j\le n-1$ we have

4.2 $$\begin{aligned} \Gamma \left( x+\tfrac{j}{n}+\tfrac{i}{2Yn^2}\right) \in {\mathcal {C}}_{Y_j,2Y_j},\quad \text { where }\ Y_{j}=\gcd (n,m+j)^2Y. \end{aligned}$$

In particular, we have

4.3 $$\begin{aligned} \left\{ \Gamma \left( x+\tfrac{j}{n}+\tfrac{i}{2Yn^2}\right) :0\le j\le n-1\right\} \subset {\mathcal {C}}_{Y}. \end{aligned}$$

#### Proof

The in particular part follows immediately from the inclusion ${\mathcal {C}}_{Y_j,2Y_j}\subset {\mathcal {C}}_{Y},$ which in turn follows from the trivial bound $Y_j\ge Y$. Hence it suffices to prove the first half of the lemma. For simplicity of notation, we set $r=1/(2Yn^2)$. Then by assumption $|x-\tfrac{m}{n}|<r$. Fix $0\le j\le n-1$, and let $\tfrac{p}{q}$ be the reduced form of $\tfrac{m+j}{n}$ (so that $q=\tfrac{n}{\gcd (n,m+j)}$). Then $x+\tfrac{j}{n}+ir \in H^{\circ }_{p/q,r}$ and $x+\tfrac{j}{n}+ir'\in H_{p/q,r}$ for some $r<r'<2r$. Take $\gamma \in \Gamma$ sending $H^{\circ }_{p/q,r}$ to the region $\left\{ z\in {\mathbb {H}}:{\mathfrak {Im}}(z)>1/(2rq^2)=Y_j\right\}$. Then we have ${\mathfrak {Im}}\left( \gamma (x+\tfrac{j}{n}+ir)\right) >Y_j$ and ${\mathfrak {Im}}\left( \gamma (x+\tfrac{j}{n}+ir')\right) =Y_j$. Since $r<r'<2r$ we can bound the hyperbolic distance

$$\begin{aligned} d_{{\mathbb {H}}}\left( \gamma (x+\tfrac{j}{n}+ir), \gamma (x+\tfrac{j}{n}+ir')\right) =\log \left( \tfrac{r'}{r}\right) <\log 2, \end{aligned}$$

implying that

$$\begin{aligned} \gamma \left( x+\tfrac{j}{n}+ir\right) \in \left\{ z\in {\mathbb {H}}:Y_j<{\mathfrak {Im}}(z)<2Y_j\right\} , \end{aligned}$$

which implies (4.2). $$\square $$

### Full escape to the cusp along subsequences for almost every translate

In this subsection we prove Theorem 4.3. Before stating this theorem, we first recall a definition from Diophantine approximation. Let $\psi : {\mathbb {N}}\rightarrow (0,1/2)$ be a non-increasing function. We say that $x\in {\mathbb {R}}$ is *primitive* $\psi$*-approximable* if there exist infinitely many $n\in {\mathbb {N}}$ such that the inequality

4.4 $$\begin{aligned} \left| x-\frac{m}{n}\right| < \frac{\psi (n)}{n} \end{aligned}$$

is satisfied by some $m\in {\mathbb {Z}}$ coprime to *n*. Since we assume $\psi ({\mathbb {N}})\subset (0,1/2)$, the existence of such an *m* implies its uniqueness. We prove the following:

#### Theorem 4.3

Let $\psi : {\mathbb {N}}\rightarrow (0,1/2)$ be a non-increasing function such that $\lim \nolimits _{n\rightarrow \infty }n\psi (n)=0$. Let $\{y_n\}_{n\in {\mathbb {N}}}$ be a sequence of positive numbers satisfying

4.5 $$\begin{aligned} r_n:=\frac{1}{2}\min \left\{ \psi (n)^{-2}y_n, n^{-2}y_n^{-1}\right\} \xrightarrow {n\rightarrow \infty }\infty . \end{aligned}$$

If $x\in [0,1)$ is primitive $\psi$-approximable, then ${\mathcal {R}}_n(x,y_n)\subset {\mathcal {C}}_{r_n}$ infinitely often.

#### Remark 4.6

Since ${\mathcal {R}}_n^{\mathrm{pr}}(x,y)\subset {\mathcal {R}}_n(x,y)$ for any $n\in {\mathbb {N}}$, $x\in {\mathbb {R}}$ and $y>0$, Theorem 4.3 also holds for translates of the primitive rational points.

#### Proof of Theorem 4.3

Let $x\in [0,1)$ be primitive $\psi$-approximable. Then for $Y_n=1/\left( 2n\psi (n)\right)$, we have by (4.3) that

4.7 $$\begin{aligned} \left\{ \Gamma \left( x+\tfrac{j}{n}+i\tfrac{\psi (n)}{n}\right) \in {\mathcal {M}}:0\le j\le n-1\right\} \subset {\mathcal {C}}_{Y_n} \end{aligned}$$

for infinitely many *n*’s.

For every $n\in {\mathbb {N}}$, set $d_n:= Y_n/r_n = \max \left\{ \psi (n)/(ny_n), ny_n/\psi (n)\right\}$. Then

4.8 $$\begin{aligned} d_{{\mathbb {H}}}(t+i\psi (n)/n, t+iy_n)=\log (d_n) \end{aligned}$$

for any $t\in {\mathbb {R}}$. As in the proof of Lemma 4.2, by (4.7) and (4.8) we have ${\mathcal {R}}_n(x,y_n)\subset {\mathcal {C}}_{Y_n/d_n}$ for any *n* in (4.7). $\square$

We now give a short

#### Proof of Theorem 1.4

Let $\alpha =\min \{\beta , 2-\beta \}$. For each $n\ge 2$, let $\psi (n)=1/(n\log n)$ and let $\{y_n\}_{n\in {\mathbb {N}}}$ be a sequence of positive numbers satisfying $y_n\asymp 1/(n^2\log ^{\beta } n)$. Then $r_n$ as in (4.5) is given by $r_n=\tfrac{1}{2}\min \{\psi (n)^{-2}y_n, n^{-2}y_n^{-1}\}\asymp \log ^{\alpha } n$. By Theorem 4.3, for any $x\in [0,1)$ primitive $\psi$-approximable, we have that ${\mathcal {R}}_n(x,y_n)\subset {\mathcal {C}}_{r_n}$ infinitely often. Hence by (4.1), for each such $x\in {\mathbb {R}}/{\mathbb {Z}}$, we have

$$\begin{aligned} \inf _{\Gamma z\in {\mathcal {R}}_n(x,y_n)}d_{{\mathcal {M}}}(\Gamma z_0, \Gamma z)\ge \log (r_n)+O(1)=\alpha \log \log n+O(1) \end{aligned}$$

infinitely often, implying the inequality (1.10). Finally, since $\sum _{n\in {\mathbb {N}}}\psi (n)=\infty$ and $\psi$ is decreasing, the set of primitive $\psi$-approximable numbers in [0, 1) is of full measure by Khintchine’s approximation theorem. $\square$

For every irrational $x\in {\mathbb {R}}$, the *Diophantine exponent* $\kappa _x>0$ is the supremum of $\kappa '>0$ for which *x* is primitive $n^{-\kappa '}$-approximable. Dirichlet’s approximation theorem implies that $\kappa _x\ge 1$ for any irrational *x* and by Khintchine’s theorem, $\kappa _x=1$ for almost every $x\in {\mathbb {R}}$. When $\kappa _x>1$, we have the following result that yields much faster cusp excursion rates for our sample points while handling sequences $\{y_n\}_{n\in {\mathbb {N}}}$ decaying polynomially faster than $1/n^2$.

#### Theorem 4.4

Let $\Gamma z_0\in {\mathcal {M}}$ be a fixed base point. Let $x\in [0,1)$ with Diophantine exponent $\kappa _x>1$ and let $\{y_n\}_{n\in {\mathbb {N}}}$ be a sequence of positive numbers satisfying $y_n\asymp n^{-\beta }$ for some fixed $2< \beta <2\kappa _x$. Then

$$\begin{aligned} \mathop {{\overline{\lim }}}_{n\rightarrow \infty }\frac{\inf _{\Gamma z\in {\mathcal {R}}_n(x,y_n)}d_{{\mathcal {M}}}\left( \Gamma z_0, \Gamma z\right) }{\log n}\ge \min \{2\kappa _x-\beta , \beta -2\}. \end{aligned}$$

#### Proof

Take $\kappa \in (1,\kappa _x)$ and set $\alpha =\min \{2\kappa -\beta ,\beta -2\}$. Let $\psi (n)=1/n^{\kappa }$. Then *x* is primitive $\psi$-approximable since $\kappa <\kappa _x$. By Theorem 4.3, we have ${\mathcal {R}}_n(x,y_n)\subset {\mathcal {C}}_{r_n}$ infinitely often with $r_n=\tfrac{1}{2}\min \{\psi (n)^{-2}y_n, n^{-2}y_n^{-1}\}\asymp n^{\alpha }$. This implies that

$$\begin{aligned} \mathop {{\overline{\lim }}}_{n\rightarrow \infty }\frac{\inf _{\Gamma z\in {\mathcal {R}}_n(x,y_n)}d_{{\mathcal {M}}}\left( \Gamma z_0, \Gamma z\right) }{\log n}\ge \alpha =\min \{2\kappa -\beta , \beta -2\}. \end{aligned}$$

Taking $\kappa \rightarrow \kappa _x$ finishes the proof. $\square$

### A non-equidistribution result for all translates

In this subsection we prove the following result which, together with part (1) of Theorem 1.3 implies non-equidistribution for all translates:

#### Theorem 4.5

Let $1/\sqrt{5}\le c< 3/2$ and let $y_n=c/n^2$. Then there exists a closed measurable subset ${\mathcal {E}}_c\subset {\mathcal {M}}$, depending only on *c*, with $\mu _{{\mathcal {M}}}({\mathcal {E}}_c)< 1$, and such that for each irrational $x\in [0,1)$, ${\mathcal {R}}_n(x,y_n)\subset {\mathcal {E}}_c$ infinitely often.

The set ${\mathcal {E}}_c$ in Theorem 4.5 is explicit: For any $c>0$, ${\mathcal {E}}_c\subset {\mathcal {M}}$ is defined to be the image of the closed set

$$\begin{aligned} \left\{ z\in {\mathbb {H}}:{\mathfrak {Im}}(z)\in [1/(2c),1/c]\cup [2/c,4/c]\cup [9/(2c),\infty )\right\} \end{aligned}$$

under the natural projection from ${\mathbb {H}}$ to ${\mathcal {M}}$. It is clear from the definition that ${\mathcal {E}}_c\subset {\mathcal {M}}$ is closed. Theorem 4.5 is a direct consequence of the following two lemmas.

#### Lemma 4.6

For any $c>0$ let $y_n=c/n^2$ and let $\psi _c(n)=c/n$. Then if $x\in [0,1)$ is primitive $\psi _c$-approximable, we have ${\mathcal {R}}_n(x, y_n)\subset {\mathcal {E}}_c$ infinitely often.

#### Proof

Let $x\in [0,1)$ be primitive $\psi _c$-approximable, that is, there exist infinitely many $n\in {\mathbb {N}}$ satisfying $\left| x-m/n\right| <c/n^2=y_n$ with some uniquely determined $m\in {\mathbb {Z}}$ satisfying $\gcd (m,n)=1$. For each such *n*, and for any $0\le j\le n-1$, let $k=\gcd (n,m+j)^2$. Then by (4.2), $\Gamma (x+j/n+iy_n)\in {\mathcal {C}}_{k^2/(2c),k^2/c}$. Moreover, since $(k^2/(2c), k^2/c)\subset [1/(2c), 1/c]\cup [2/c, 4/c]\cup [9/(2c),\infty )$ for any $k\in {\mathbb {N}}$, we have ${\mathcal {C}}_{k^2/(2c),k^2/c}\subset {\mathcal {E}}_c$ for any $k\in {\mathbb {N}}$, implying that ${\mathcal {R}}_n(x,y_n)\subset {\mathcal {E}}_c$ for these infinitely many $n\in {\mathbb {N}}$. $\square$

#### Lemma 4.7

For any $0<c< 3/2$, we have $\mu _{{\mathcal {M}}}({\mathcal {E}}_c)\le 1-\frac{3}{\pi }\left( \frac{1}{\max \{2c,4/c\}}-\frac{2c}{9}\right) <1$.

#### Proof

Let ${\mathcal {U}}\subset {\mathcal {M}}$ be the projection of the open set

$$\begin{aligned} \left\{ z\in {\mathbb {H}}: \max \left\{ 2c,4/c\right\}<{\mathfrak {Im}}(z)<9/(2c)\right\} . \end{aligned}$$

Since $0<c<3/2$ we have $\max \{2c, 4/c\}<9/(2c)$ implying that ${\mathcal {U}}$ is nonempty. We will show that ${\mathcal {E}}_c$ is disjoint from ${\mathcal {U}}$. Let $I_1= [1/(2c),1/c]$, $I_2= [2/c,4/c]$ and $I_3=[9/(2c),\infty )$, and for $1\le j\le 3$, define ${\mathcal {E}}_c^j$ to be the projection onto ${\mathcal {M}}$ of $\{z\in {\mathbb {H}}:{\mathfrak {Im}}(z)\in I_j\}$ such that ${\mathcal {E}}_c=\bigcup _{j=1}^3{\mathcal {E}}_c^j$. It thus suffices to show that ${\mathcal {E}}_c^j\cap {\mathcal {U}}=\emptyset$ for each $1\le j\le 3$. For this, we identify (up to a null set) ${\mathcal {M}}$ with the standard fundamental domain ${\mathcal {F}}_{\Gamma }:=\left\{ z\in {\mathbb {H}}:{\mathfrak {Re}}(z)<\frac{1}{2}, |z|>1\right\}$. Since $0<c<3/2$, we have $\max \left\{ 2c,4/c\right\}>2/c>2/(3/2)>1$. Thus we have

$$\begin{aligned} {\mathcal {U}}=\left\{ z\in {\mathcal {F}}_{\Gamma }: \max \left\{ 2c,4/c\right\}<{\mathfrak {Im}}(z)<9/(2c)\right\} ,\qquad {\mathcal {E}}_c^j=\left\{ z\in {\mathcal {F}}_{\Gamma }:{\mathfrak {Im}}(z)\in I_j\right\} \end{aligned}$$

for $j= 2, 3$. Moreover, since the interval $( \max \left\{ 2c,4/c\right\} , 9/2c)$ intersects $I_{2}$ and $I_3$ trivially, we have ${\mathcal {E}}_c^j\cap {\mathcal {U}}=\emptyset$ for $j=2,3$. It thus remains to show that ${\mathcal {E}}_c^1\cap {\mathcal {U}}=\emptyset$. For this we note that $z\in {\mathcal {F}}_{\Gamma }$ satisfies the property that

$$\begin{aligned} {\mathfrak {Im}}(z)=\max _{\gamma \in \Gamma }{\mathfrak {Im}}(\gamma z). \end{aligned}$$

Hence to show ${\mathcal {E}}_c^1\cap {\mathcal {U}}=\emptyset$, it suffices to show that $\max _{\gamma \in \Gamma }{\mathfrak {Im}}(\gamma z)\le \max \left\{ 2c,4/c\right\}$ for any $z=s+it\in {\mathbb {H}}$ with ${\mathfrak {Im}}(z)=t\in I_1=[1/(2c), 1/c]$. For this, using the same discussion as in the proof of Lemma 4.1 we have for any $z=s+it\in {\mathbb {H}}$ with $t\in [1/(2c), 1/c]$

$$\begin{aligned} \max _{\gamma \in \Gamma }{\mathfrak {Im}}(\gamma z)\le \max \left\{ t,t^{-1}\right\} \le \max \left\{ 1/c,2c\right\} \le \max \left\{ 2c,4/c\right\} . \end{aligned}$$

Finally, using the above description of ${\mathcal {U}}$ and (2.1) we have by direct computation

$$\begin{aligned} \mu _{{\mathcal {M}}}({\mathcal {U}})=\frac{3}{\pi }\left( \frac{1}{\max \{2c,4/c\}}-\frac{2c}{9}\right) \end{aligned}$$

implying that $\mu _{{\mathcal {M}}}({\mathcal {E}}_c)\le 1-\frac{3}{\pi }\left( \frac{1}{\max \{2c,4/c\}}-\frac{2c}{9}\right) <1$ (again since $0<c<3/2$).

$$\square $$

#### Proof of Theorem 4.5

Let $\psi _c(n)=c/n$. Since $c\ge 1/\sqrt{5}$, any irrational number is primitive $\psi _c$-approximable by the Hurwitz’s approximation theorem; see, e.g., [14, Theorem 193]. Hence by Lemma 4.6, for each irrational $x\in [0,1)$, we have ${\mathcal {R}}_n(x,y_n)\subset {\mathcal {E}}_c$ infinitely often. Moreover, since $c<3/2$ by Lemma 4.7 we have $\mu _{{\mathcal {M}}}({\mathcal {E}}_c)<1$, finishing the proof. $\square$

#### Remark 4.9

The condition on the sequence $\{y_n\}_{n\in {\mathbb {N}}}$ in Theorem 4.5 is quite restrictive and the proof of Theorem 4.5 is much more involved than that of Theorem 4.3. We note that this is because we need to take care of the *badly approximable numbers*, that is, the set of irrational numbers that are not primitive $\psi _c$-approximable for some $c>0$. If $x\in [0,1)$ is not badly approximable, then a similar argument as in the proof of Theorem 4.3 using only the crude estimate (4.3) would already be sufficient to prove non-equidistribution of the sample points ${\mathcal {R}}_n(x,y_n)$ for any sequence $\{y_n\}_{n\in {\mathbb {N}}}$ satisfying $y_n\asymp 1/n^2$.

## Second moments of the discrepancy

Let $\Gamma ={\text {SL}}_2({\mathbb {Z}})$ and let ${\mathcal {M}}=\Gamma \backslash {\mathbb {H}}$ be the modular surface as before. In this section we prove Theorem 1.6. Our proof relies on a second moment computation of the discrepancies $|\delta _{n,x,y}-\mu _{{\mathcal {M}}}|$ and $|\delta _{n,x,y}^{\mathrm{pr}}-\mu _{{\mathcal {M}}}|$ along the closed horocycle ${\mathcal {H}}_y$. Throughout this section, we abbreviate the second moments $\int _0^1\left| \delta _{n,x,y}(\Psi )-\mu _{{\mathcal {M}}}(\Psi )\right| ^2dx$ and $\int _0^1\left| \delta ^{\mathrm{pr}}_{n,x,y}(\Psi )-\mu _{{\mathcal {M}}}(\Psi )\right| ^2dx$ by $D_{n,y}(\Psi )$ and $D_{n,y}^{\mathrm{pr}}(\Psi )$ respectively. Since we assume $\Gamma ={\text {SL}}_2({\mathbb {Z}})$ we will also use the notation $\mu _{\Gamma }$ for $\mu _{{\mathcal {M}}}$.

### Relation to Hecke operators

In this subsection we prove two preliminary estimates relating these second moments to the Hecke operators defined in Sect. 2.3.

#### Proposition 5.1

For any $n\in {\mathbb {N}}$, $y>0$ and $\Psi \in C_c^{\infty }({\mathcal {M}})$, we have

5.1 $$\begin{aligned} D_{n,y}(\Psi )=\frac{1}{n}\sum _{j=0}^{n-1}\left\langle \Psi _0, {\widetilde{T}}_{u_{j/n}}(\Psi _0)\right\rangle +O\left( {\mathcal {S}}(\Psi )y^{1/2}\right) , \end{aligned}$$

and

5.2 $$\begin{aligned} D_{n,y}^{\mathrm{pr}}(\Psi )\le \frac{1}{\varphi (n)}\sum _{j=0}^{n-1}\left| \left\langle \Psi _0, {\widetilde{T}}_{u_{j/n}}(\Psi _0)\right\rangle \right| +O\left( {\mathcal {S}}(\Psi )y^{1/2}\right) . \end{aligned}$$

where $\Psi _0=\Psi -\mu _{\Gamma }(\Psi )$, ${\widetilde{T}}_{u_{j/n}}$ is the Hecke operator associated to $u_{j/n}\in {\text {SL}}_2({\mathbb {Q}})$ defined as in (2.9), the Sobolev norm ${\mathcal {S}}(\Psi )$ is defined by

5.3 $$\begin{aligned} {\mathcal {S}}(\Psi ):={\mathcal {S}}_{4,2}^{\Gamma }(\Psi )^2+{\mathcal {S}}_{2,2}^{\Gamma }(\Psi ){\mathcal {S}}_{1,0}^{\Gamma }(\Psi ), \end{aligned}$$

and the implied constants are absolute.

#### Proof

Without loss of generality we may assume that $\Psi$ is real-valued. Expanding the square in the left hand side of (5.1), doing a change of variables, and using the left $u_1$-invariance of $\Psi$, we have that $D_{n,y}(\Psi )$ equals

$$\begin{aligned}&\frac{1}{n^2}\sum _{j_1,j_2=0}^{n-1}\int _0^1\Psi (x+\tfrac{j_1}{n}+iy)\Psi (x+\tfrac{j_2}{n}+iy)dx\\ {}&\quad -2\mu _{\Gamma }(\Psi ) \frac{1}{n}\sum _{j=0}^{n-1}\int _0^1\Psi (x+\tfrac{j}{n}+iy)dx+\mu _{\Gamma }(\Psi )^2\\&\quad =\frac{1}{n}\sum _{j=0}^{n-1}\int _0^1\Psi (x+iy)\Psi (x+\tfrac{j}{n}+iy)dx-2\mu _{\Gamma }(\Psi )\int _0^1\Psi (x+iy)dx+\mu _{\Gamma }(\Psi )^2. \end{aligned}$$

Applying (2.14) to the term $\int _0^1\Psi (x+iy)dx$ and using the trivial estimate

5.4 $$\begin{aligned} \Vert \Psi \Vert _2^{3/4}\Vert \Delta \Psi \Vert ^{1/4}\left| \mu _{\Gamma }(\Psi )\right| \le {\mathcal {S}}^{\Gamma }_{2,2}(\Psi ){\mathcal {S}}_{1,0}^{\Gamma }(\Psi )\le {\mathcal {S}}(\Psi ), \end{aligned}$$

we get

5.5 $$\begin{aligned} D_{n,y}(\Psi )=\frac{1}{n}\sum _{j=0}^{n-1}\int _0^1\Psi (x+iy)\Psi (x+\tfrac{j}{n}+iy)dx-\mu _{\Gamma }(\Psi )^2+O({\mathcal {S}}(\Psi )y^{1/2}).\nonumber \\ \end{aligned}$$

For each $0\le j\le n-1$, let $\Gamma ^j:=\Gamma ^{u_{j/n}}=\Gamma \cap u_{j/n}^{-1}\Gamma u_{j/n}$ and define $F_j(\Psi ):=\Psi L_{u_{j/n}^{-1}}\Psi \in C^{\infty }({\mathbb {H}})$. Since $\Psi$ is left $\Gamma$-invariant, and $L_{u_{j/n}^{-1}}\Psi$ is left $u_{j/n}^{-1}\Gamma u_{j/n}$-invariant, we have $F_j(\Psi )\in C^{\infty }(\Gamma ^j\backslash {\mathbb {H}})$. Moreover,

$$\begin{aligned} F_j(\Psi )(x+iy)=\Psi (x+iy)\Psi (x+\tfrac{j}{n}+iy). \end{aligned}$$

For each $0\le j\le n-1$, it is easy to check that $u_1\in \Gamma ^j$ and $\Gamma ^j$ contains the principal congruence subgroup $\Gamma (n^2)$, hence $\Gamma ^j$ satisfies the assumptions in Proposition 2.3. Then by (2.14),

$$\begin{aligned}&\int _0^1 F_j(\Psi )(x+iy)dx \\&\quad = \int _{\Gamma ^j\backslash {\mathbb {H}}} F_j(\Psi )(z) d\mu _{\Gamma ^j}(z) + O\left( \Vert F_j(\Psi )\Vert _2^{3/4}\Vert \Delta F_j(\Psi )\Vert _2^{1/4} y^{1/2}\right) . \end{aligned}$$

Next we note that by (2.3),

$$\begin{aligned}&\Vert F_j(\Psi )\Vert _2^{3/4}\Vert \Delta F_j(\Psi )\Vert _2^{1/4}\le {\mathcal {S}}^{\Gamma ^j}_{2,2}\left( F_j(\Psi )\right) \\&\quad ={\mathcal {S}}^{\Gamma ^j}_{2,2}\left( \Psi L_{u_{j/n}^{-1}}\Psi \right) \le {\mathcal {S}}_{4,2}^{\Gamma ^j}\left( \Psi \right) {\mathcal {S}}_{4,2}^{\Gamma ^j}\left( L_{u_{j/n}^{-1}}\Psi \right) . \end{aligned}$$

Using the fact that $\Psi$ is left $\Gamma$-invariant and $\Gamma ^j$ is a finite-index subgroup of $\Gamma$, by (2.4), ${\mathcal {S}}_{4,2}^{\Gamma ^j}(\Psi )={\mathcal {S}}_{4,2}^{\Gamma }(\Psi )$. Similarly, we have

$$\begin{aligned} {\mathcal {S}}_{4,2}^{\Gamma ^j}\left( L_{u_{j/n}^{-1}}\Psi \right) ={\mathcal {S}}_{4,2}^{u_{j/n}^{-1}\Gamma u_{j/n}}\left( L_{u_{j/n}^{-1}}\Psi \right) ={\mathcal {S}}_{4,2}^{\Gamma }\left( \Psi \right) , \end{aligned}$$

where for the second equality we used (2.2). Hence we have

5.6 $$\begin{aligned} \Vert F_j(\Psi )\Vert _2^{3/4}\Vert \Delta F_j(\Psi )\Vert _2^{1/4}\le {\mathcal {S}}^{\Gamma ^j}_{2,2}\left( F_j(\Psi )\right) \le {\mathcal {S}}_{4,2}^{\Gamma }(\Psi )^2\le {\mathcal {S}}(\Psi )<\infty . \end{aligned}$$

Thus applying (2.14) to $F_j\in C^{\infty }(\Gamma ^j\backslash {\mathbb {H}})$ and using (5.6) we get

5.7 $$\begin{aligned} \int _0^1\Psi (x+iy)\Psi \left( x+\tfrac{j}{n}+iy\right) dx=\left\langle \Psi , L_{u_{j/n}^{-1}}\Psi \right\rangle _{L^2(\Gamma ^j\backslash {\mathbb {H}})}+O\left( {\mathcal {S}}(\Psi )y^{1/2}\right) . \end{aligned}$$

Plugging (5.7) into (5.5) and using the identities $\mu _{\Gamma }(\Psi )=\mu _{\Gamma ^j}(\Psi )=\mu _{\Gamma ^j}(L_{u_{j/n}^{-1}}\Psi )$ (the second equality follows from the left *G*-invariance of the hyperbolic area $\mu _{\Gamma ^j}$) we get that

$$\begin{aligned} D_{n,y}(\Psi )=\frac{1}{n}\sum _{j=0}^{n-1}\left\langle \Psi _0, L_{u_{j/n}^{-1}}\Psi _0\right\rangle _{L^2(\Gamma ^j\backslash {\mathbb {H}})}+O({\mathcal {S}}(\Psi )y^{1/2}). \end{aligned}$$

Let ${\mathcal {F}}_{\Gamma }\subset {\mathbb {H}}$ be a fundamental domain for $\Gamma \backslash {\mathbb {H}}$. The disjoint union $\bigsqcup _{\gamma \in \Gamma ^j\backslash \Gamma }\gamma {\mathcal {F}}_{\Gamma }$ forms a fundamental domain for $\Gamma ^{j}\backslash {\mathbb {H}}$. Thus we can conclude the proof of (5.1) by noting that

$$\begin{aligned}&\int _{\bigsqcup _{\gamma \in \Gamma ^j\backslash \Gamma }\gamma {\mathcal {F}}_{\Gamma }}\Psi _0(z)\Psi _0(u_{j/n}z)d\mu _{\Gamma ^j}(z)=\sum _{\gamma \in \Gamma ^j\backslash \Gamma }\int _{\gamma {\mathcal {F}}_{\Gamma }}\Psi _0(z)\Psi _0(u_{j/n}z)d\mu _{\Gamma ^j}(z)\\&\quad =\int _{{\mathcal {F}}_{\Gamma }}\Psi _0(z)\left( \frac{1}{[\Gamma : \Gamma ^j]}\sum _{\gamma \in \Gamma ^j\backslash \Gamma }\Psi _0(u_{j/n}\gamma z)\right) d\mu _{\Gamma }(z)\\&=\int _{{\mathcal {F}}_{\Gamma }}\Psi _0(z){\widetilde{T}}_{u_{j/n}}(\Psi _0)(z)d\mu _{\Gamma }(z), \end{aligned}$$

where for the second equation we did a change of variable $z\mapsto \gamma z$, used the left $\Gamma$-invariance of $\Psi$ and the relation $[\Gamma :\Gamma ^j]\mu _{\Gamma ^j}=\mu _{\Gamma }$, and for the last equality we used the expression (2.10). Similarly, applying the estimates (2.14) and (5.4) and making change of variables we see that $D_{n,y}^{\mathrm{pr}}(\Psi )$ equals

$$\begin{aligned} \frac{1}{\varphi (n)^2}\sum _{j=0}^{n-1}c(j)\int _0^1\Psi (x+iy)\Psi (x+\tfrac{j}{n}+iy)dx-\mu _{\Gamma }(\Psi )^2+O\left( {\mathcal {S}}(\Psi )y^{1/2}\right) , \end{aligned}$$

where

$$\begin{aligned} c(j):=\#\left\{ ([j_1], [j_2])\in ({\mathbb {Z}}/n{\mathbb {Z}})^{\times }\times ({\mathbb {Z}}/n{\mathbb {Z}})^{\times }: [j_2]-[j_1]=[j]\right\} . \end{aligned}$$

Now similar as before we can apply the estimate (5.7), the identities $\mu _{\Gamma }(\Psi )=\mu _{\Gamma ^j}(\Psi )=\mu _{\Gamma ^j}(L_{u_{j/n}^{-1}}\Psi )$ and $\sum _{j=0}^{n-1}c(j)=\varphi (n)^2$ to get

$$\begin{aligned} D_{n,y}^{\mathrm{pr}}(\Psi )&=\frac{1}{\varphi (n)^2}\sum _{j=0}^{n-1}c(j)\left\langle \Psi _0, L_{u_{j/n}^{-1}}\Psi _0\right\rangle _{L^2(\Gamma ^j\backslash {\mathbb {H}})}+O({\mathcal {S}}(\Psi )y^{1/2})\\&=\frac{1}{\varphi (n)^2}\sum _{j=0}^{n-1}c(j)\left\langle \Psi _0, {\widetilde{T}}_{u_{j/n}}(\Psi _0)\right\rangle _{L^2(\Gamma \backslash {\mathbb {H}})}+O({\mathcal {S}}(\Psi )y^{1/2}). \end{aligned}$$

Finally we can finish the proof by noting that for each $0\le j\le n-1$, $c(j)\le \varphi (n)$ (since for each $[j_1]\in ({\mathbb {Z}}/n{\mathbb {Z}})^{\times }$, there is at most one $[j_2]\in ({\mathbb {Z}}/n{\mathbb {Z}})^{\times }$ such that $[j_2]-[j_1]=[j]$). $\square$

### Second moment estimates

Combining Proposition 5.1 and the operator norm bound in Proposition 2.2 we have the following second moment estimates:

#### Theorem 5.2

For any $n\in {\mathbb {N}}$, $y>0$ and $\Psi \in C_c^{\infty }({\mathcal {M}})$ we have

5.8 $$\begin{aligned} \max \left\{ D_{n,y}(\Psi ), D^{\mathrm{pr}}_{n,y}(\Psi )\right\} \ll _{\epsilon }n^{-1+2\theta +\epsilon }\Vert \Psi \Vert _2^2+{\mathcal {S}}(\Psi )y^{1/2}, \end{aligned}$$

where $\theta =7/64$ is the best bound towards the Ramanujan conjecture as before and the Sobolev norm ${\mathcal {S}}(\Psi )$ is as defined in (5.3).

#### Remark 5.9

It is also possible to approach the second moment computation using the spectral bounds on the Fourier coefficients of $\Psi$ from Sect. 3.1 rather than Hecke operators. The spectral approach however yields a weaker estimate when $y>0$ is small. For comparison, following the spectral approach, one obtains

$$\begin{aligned} \int _0^1 |\delta _{n,x,y}(\Psi )-\mu _\Gamma (\Psi )|^2 dx\ \ll _\epsilon \left( n^{-1}y^{-2(\theta +\epsilon )}+y^{1/2}\right) {\mathcal {S}}_{2,2}(\Psi ). \end{aligned}$$

#### Proof of Theorem 5.2

First we prove (5.8). For each $0\le j\le n-1$, it is clear that $u_{j/n}$ is of degree $n_j:=n/\gcd (n,j)$, and thus ${\widetilde{T}}_{u_{j/n}}={\widetilde{T}}_{n_j}$. Applying (5.1), (5.2), the estimate $\varphi (n)\gg _{\epsilon } n^{-1+\epsilon /2}$ and the operator norm bound in Proposition 2.2 to the terms $\left\langle \Psi _0, {\widetilde{T}}_{n_j}\Psi _0\right\rangle$, we get

$$\begin{aligned} \max \left\{ D_{n,y}(\Psi ), D^{\mathrm{pr}}_{n,y}(\Psi )\right\}&\ll _{\epsilon }\ n^{-1+\epsilon /2}\sum _{j=0}^{n-1}n_j^{-1+2\theta +\epsilon /4}\Vert \Psi _0\Vert _2^2+{\mathcal {S}}(\Psi )y^{1/2}. \end{aligned}$$

For any $d\mid n$, $\#\{0\le j\le n-1:n_j=d\}=\varphi (d)$, thus

$$\begin{aligned} \sum _{j=1}^nn_j^{-1+2\theta +\epsilon /4}=\sum _{d| n}\varphi (d)d^{-1+2\theta +\epsilon /4}< \sum _{d\mid n}d^{2\theta +\epsilon /4}=\sigma _{2\theta +\epsilon /4}(n)\ll _{\epsilon }n^{2\theta +\epsilon /2}, \end{aligned}$$

where for the first inequality we used the trivial bound $\varphi (d)<d$. Finally, we observe that $\Vert \Psi _0\Vert _2\le \Vert \Psi \Vert _2$. $\square$

We now give a quick

#### Proof of Theorem 1.6

Let $\alpha >0$ be the fixed number as in this theorem. Let $\beta :=\min \{\frac{\alpha }{2}, 1-2\theta \}$. Fix $0<c< \beta$ and let ${\mathcal {N}}\subset {\mathbb {N}}$ be an unbounded subsequence such that $\sum _{n\in {\mathcal {N}}}n^{-c}<\infty$. We want to show that for any $\{y_n\}_{n\in {\mathbb {N}}}$ satisfying $y_n\ll n^{-\alpha }$ there exists a full measure subset $I\subset {\mathbb {R}}/{\mathbb {Z}}$ such that for any $x\in I$, $\delta _{n,x,y_n}(\Psi )\rightarrow \mu _{{\mathcal {M}}}(\Psi )$ and $\delta ^{\mathrm{pr}}_{n,x,y_n}(\Psi )\rightarrow \mu _{{\mathcal {M}}}(\Psi )$ for any $\Psi \in C_c^{\infty }({\mathcal {M}})$ as $n\in {\mathcal {N}}$ goes to infinity. Since the function space $C_c^{\infty }({\mathcal {M}})$ has a dense countable subset, it suffices to prove the above assertion for a fixed $\Psi$. Now we fix $\Psi \in C_c^{\infty }({\mathcal {M}})$ and take $\epsilon >0$ sufficiently small such that $\beta -2\epsilon >c$. For any $n\in {\mathbb {N}}$ define $I_{n}=I_n^1\cup I_n^2\subset {\mathbb {R}}/{\mathbb {Z}}$ such that

$$\begin{aligned} I_n^1:=\left\{ x\in {\mathbb {R}}/{\mathbb {Z}}:\left| \delta _{n,x,y_n}(\Psi )-\mu _{{\mathcal {M}}}(\Psi )\right| >n^{-\epsilon /2}\right\} , \end{aligned}$$

and

$$\begin{aligned} I_n^2:=\left\{ x\in {\mathbb {R}}/{\mathbb {Z}}:\left| \delta ^{\mathrm{pr}}_{n,x,y_n}(\Psi )-\mu _{{\mathcal {M}}}(\Psi )\right| >n^{-\epsilon /2}\right\} . \end{aligned}$$

Thus by the second moment estimate (5.8), the assumption that $y_n\ll n^{-\alpha }$ and Chebyshev’s inequality we get

$$\begin{aligned} \left| I_n\right| \le \left| I_n^1\right| +\left| I_n^2\right| \le 2n^{\epsilon }\max \left\{ D_{n,y}(\Psi ), D^{\mathrm{pr}}_{n,y}(\Psi )\right\} \ll _{\epsilon ,\Psi }{{n^{-\beta +2\epsilon }<n^{-c}}}, \end{aligned}$$

implying that $\sum _{n\in {\mathcal {N}}}|I_n|<\infty$. Hence taking $I\subset {\mathbb {R}}/{\mathbb {Z}}$ to be the complement of this limsup set $$\mathop {{\overline{\lim }}}_{\begin{array}{c} n\in {\mathcal {N}}\\ n\rightarrow \infty \end{array}}I_n\subset {\mathbb {R}}/{\mathbb {Z}}$$ and by the Borel–Cantelli lemma we have *I* is of full measure. Moreover, for any $x\in I$, $x\in I_n^c$ for all $n\in {\mathcal {N}}$ sufficiently large, that is,

$$\begin{aligned}&\max \left\{ \left| \delta _{n,x,y_n}(\Psi )-\mu _{{\mathcal {M}}}(\Psi )\right| , \left| \delta ^{\mathrm{pr}}_{n,x,y_n}(\Psi )-\mu _{{\mathcal {M}}}(\Psi )\right| \right\} \\&\quad \le n^{-\epsilon /2},\quad \forall \ n\in {\mathcal {N}}\ \text {sufficiently large}. \end{aligned}$$

In particular for such *x*, $\delta _{n,x,y_n}(\Psi )\rightarrow \mu _{{\mathcal {M}}}(\Psi )$ and $\delta ^{\mathrm{pr}}_{n,x,y_n}(\Psi )\rightarrow \mu _{{\mathcal {M}}}(\Psi )$ as $n\in {\mathcal {N}}$ goes to infinity. $\square$

#### Remark 5.10

The second moment $D_{n,y}(\Psi )$ is closely related to the sample points (1.2) considered in [12]: Using the extra invariance $\delta _{n,x+1/n,y}(\Psi )=\delta _{n,x,y}(\Psi )$ and applying a change of variable, one can easily check that

$$\begin{aligned} D_{n,y}(\Psi )=\int _0^1\left| \frac{1}{n}\sum _{j=0}^{n-1}\Psi \left( \tfrac{x+j}{n}+iy\right) -\mu _{\Gamma }(\Psi )\right| ^2dx. \end{aligned}$$

Thus let ${\mathcal {N}}\subset {\mathbb {N}}$ be the fixed sequence as in the above proof, by Theorem 5.2 and the same Borel–Cantelli type argument we have that for almost every $x\in {\mathbb {R}}/{\mathbb {Z}}$ the sequence of sample points $\{\Gamma (\tfrac{x+j}{n}+iy_n: 0\le j\le n-1\}$ equidistributes on ${\mathcal {M}}$ with respect to $\mu _{{\mathcal {M}}}$ as $n\in {\mathcal {N}}$ goes to infinity, as long as $\{y_n\}_{n\in {\mathbb {N}}}$ decays at least polynomially.

## Left regular action of normalizing elements

In this section, $\Gamma$ denotes a congruence subgroup, and we set by $\Gamma _1={\text {SL}}_2({\mathbb {Z}})$. We moreover assume that there exists some $h\in {\text {SL}}_2({\mathbb {Q}})$ normalizing $\Gamma$, that is, $h^{-1}\Gamma h=\Gamma$. It induces the left regular *h*-action on $\Gamma \backslash {\mathbb {H}}$ given by $\Gamma z\in \Gamma \backslash {\mathbb {H}}\mapsto \Gamma hz\in \Gamma \backslash {\mathbb {H}}$. Since *h* normalizes $\Gamma$, this map is well defined: Suppose $\Gamma z=\Gamma z'$, that is there exists some $\gamma \in \Gamma$ such that $z'=\gamma z$. Then $\Gamma hz'=\Gamma h\gamma z=\Gamma h\gamma h^{-1}hz=\Gamma hz$. The goal of this section is to describe this action on cylindrical cuspidal neighborhoods of $\Gamma \backslash {\mathbb {H}}$.

### Cusp neighborhoods of congruence surfaces

Since $\Gamma$ is a congruence subgroup, the set of cusps of $\Gamma$ can be parameterized by the coset $\Gamma \backslash \left( {\mathbb {Q}}\cup \{\infty \}\right)$ (see e.g. [21, p. 222]), where the action of $\Gamma$ on ${\mathbb {Q}}\cup \{\infty \}$ is defined via the Möbius transformation. We denote by $\Omega _{\Gamma }$ a complete list of coset representatives for $\Gamma \backslash \left( {\mathbb {Q}}\cup \{\infty \}\right)$. For each cusp representative $\mathfrak {c}\in \Omega _{\Gamma }$, its stabilizer subgroup is given by

$$\begin{aligned} \Gamma _{\mathfrak {c}}:=\tau _{\mathfrak {c}}N\tau _{\mathfrak {c}}^{-1}\cap \Gamma , \end{aligned}$$

where $\tau _{\mathfrak {c}}\in \Gamma _1$ is such that $\tau _{\mathfrak {c}}\infty =\mathfrak {c}$. (More precisely, $\Gamma _{\mathfrak {c}}$ is an index two subgroup of the stabilizer subgroup of $\mathfrak {c}$ if $-I_2\in \Gamma$.) The existence of such $\tau _{\mathfrak {c}}$ is guaranteed by the transitivity of the action of $\Gamma _1$ on ${\mathbb {Q}}\cup \{\infty \}$. On the other hand, $\tau _{\mathfrak {c}}$ is only unique up to right multiplication by any element of $\pm N$. We note that $\Gamma _{\mathfrak {c}}$ is independent of the choice of $\tau _{\mathfrak {c}}$, and since $\mathfrak {c}\in \Omega _{\Gamma }$ is a cusp, $\Gamma _{\mathfrak {c}}$ is nontrivial. Moreover, $\tau _{\mathfrak {c}}^{-1}\Gamma _{\mathfrak {c}}\tau _{\mathfrak {c}}$ is a subgroup of $N\cap \Gamma _1=\langle u_1\rangle$. Hence $\tau _{\mathfrak {c}}^{-1}\Gamma _{\mathfrak {c}}\tau _{\mathfrak {c}}$ is a cyclic group generated by a unipotent matrix $u_{\omega _{\mathfrak {c}}}$ for some positive integer $\omega _{\mathfrak {c}}$, which is called the *width* of the cusp $\mathfrak {c}$.

We can now define cusp neighborhoods on the hyperbolic surface $\Gamma \backslash {\mathbb {H}}$ around a cusp $\mathfrak {c}\in \Omega _\Gamma$. For any $Y>0$, ${\mathcal {C}}_Y^{\Gamma ,\mathfrak {c}}\subset \Gamma \backslash {\mathbb {H}}$ denote the projection of the horodisc $\{\tau _\mathfrak {c}z\in {\mathbb {H}}: {\mathfrak {Im}}(z)>Y\}$ onto $\Gamma \backslash {\mathbb {H}}$. Similarly, for any $Y'>Y>0$, let ${\mathcal {C}}_{Y,Y'}^{\Gamma ,\mathfrak {c}}$ denote the projection of the cylindrical region $\{\tau _\mathfrak {c}z\in {\mathbb {H}}:Y<{\mathfrak {Im}}(z)<Y'\}$ onto $\Gamma \backslash {\mathbb {H}}$. We record the following two lemmas for the later purpose of computing the measure of certain unions of cusp neighborhoods.

#### Lemma 6.1

If $Y'>Y>1$, the set ${\mathcal {C}}_{Y,Y'}^{\Gamma ,\mathfrak {c}}$ is in one-to-one correspondence with the set

6.1 $$\begin{aligned} \{\tau _c z\in {\mathbb {H}}: {\mathfrak {Re}}(z)\in {\mathbb {R}}/\omega _\mathfrak {c}{\mathbb {Z}},\ {\mathfrak {Im}}(z)\in (Y,Y')\}. \end{aligned}$$

In particular, if $-I_2\in \Gamma$ then for any $Y'>Y>1$

6.2 $$\begin{aligned} \mu _\Gamma \left( {\mathcal {C}}_{Y,Y'}^{\Gamma ,\mathfrak {c}}\right) = \frac{3\omega _\mathfrak {c}}{\pi [\Gamma _1:\Gamma ]}\left( \frac{1}{Y}-\frac{1}{Y'}\right) . \end{aligned}$$

#### Proof

The one-to-one correspondence is given by the projection of the above rectangular set onto $\Gamma \backslash {\mathbb {H}}$. Indeed, since $\Gamma _\mathfrak {c}\subset \Gamma$, this map projects the rectangular set in (6.1) onto ${\mathcal {C}}_{Y,Y'}^{\Gamma ,\mathfrak {c}}$. To show that it is also injective, suppose $\Gamma \tau _\mathfrak {c}z=\Gamma \tau _\mathfrak {c}z'$ for some $z, z'$ from this rectangular set. Then there exists some $\gamma \in \Gamma$ such that $\tau _\mathfrak {c}^{-1}\gamma \tau _\mathfrak {c}z=z'$. If $\gamma \in \pm \Gamma _\mathfrak {c}$ then $\tau _\mathfrak {c}^{-1}\gamma \tau _\mathfrak {c}\in \pm \langle u_{\omega _\mathfrak {c}}\rangle$, and this implies that $z=z'$. Otherwise, let $$\tau _\mathfrak {c}^{-1}\gamma \tau _\mathfrak {c}=\left( {\begin{matrix} a&{} b\\ c&{}d \end{matrix}}\right) \in \Gamma _1$$. Since $\gamma \not \in \pm \Gamma _\mathfrak {c}$, $c\ne 0$. We easily see this cannot happen since it would imply

$$\begin{aligned} {\mathfrak {Im}}(z') = \frac{{\mathfrak {Im}}(z)}{|cz+d|^2}=\frac{{\mathfrak {Im}}(z)}{(cx+d)^2 +c^2y^2}\le \frac{1}{y}\le 1, \end{aligned}$$

contradicting that ${\mathfrak {Im}}(z')>Y>1$. For the area computation, we use the definition (2.1) of $\mu _{\Gamma _1}$ together with $\mu _{\Gamma _1}=[\Gamma _1:\Gamma ]\mu _\Gamma$ (since $-I_2\in \Gamma$). $\square$

#### Lemma 6.2

Given two distinct cusps $\mathfrak {c}_1$, $\mathfrak {c}_2\in \Omega _\Gamma$, and any $Y_1$, $Y_2\ge 1$, ${\mathcal {C}}_{Y_1}^{\Gamma ,\mathfrak {c}_1}\cap {\mathcal {C}}_{Y_2}^{\Gamma ,\mathfrak {c}_2}=\emptyset .$

#### Proof

Since $Y_1, Y_2\ge 1$, the sets $\{\tau _{\mathfrak {c}_1} z\in {\mathbb {H}}: {\mathfrak {Im}}(z)>Y_1\}$ and $\{\tau _{\mathfrak {c}_2} z\in {\mathbb {H}}:{\mathfrak {Im}}(z)>Y_2\}$ are subsets of the interior of the Ford circles based at $\mathfrak {c}_1$ and $\mathfrak {c}_2$ respectively. Two Ford circles are either disjoint or identical. Suppose $\Gamma z\in {\mathcal {C}}_{Y_1}^{\Gamma ,\mathfrak {c}_1}\cap {\mathcal {C}}_{Y_2}^{\Gamma ,\mathfrak {c}_2}$. Then there exists an isometry $\gamma \in \Gamma$ that maps the Ford circle at $\mathfrak {c}_1$ to the Ford circle at $\mathfrak {c}_2$. Consequently, we must have $\gamma \mathfrak {c}_1=\mathfrak {c}_2$, which is a contradiction. $\square$

#### Remark 6.3

We will later consider sets $I_{y,Y,\mathfrak {c}}:=\left\{ x\in (0,1):\Gamma (x+iy)\in {\mathcal {C}}_Y^{\Gamma ,\mathfrak {c}}\right\}$ for some $y>0, Y>1$ and $\mathfrak {c}\in \Omega _{\Gamma }$. This set is the intersection of the line segment $\{x+iy\in {\mathbb {H}}:0<x<1\}$ with the preimage of ${\mathcal {C}}_Y^{\Gamma ,\mathfrak {c}}$ in ${\mathbb {H}}$ (under the natural projection from ${\mathbb {H}}$ to $\Gamma \backslash {\mathbb {H}}$). By definition the preimage of ${\mathcal {C}}_Y^{\Gamma ,\mathfrak {c}}$ is the disjoint (since $Y>1$) union of the infinitely many horodiscs $\left\{ \tau _{\mathfrak {c}'}z\in {\mathbb {H}}:{\mathfrak {Im}}(z)>Y\right\} =H^{\circ }_{p/q,1/(2q^2Y)}$ for all cusps $\mathfrak {c}'=p/q\in \Gamma \mathfrak {c}$. Moreover, note that a necessary condition for such a horodisc intersecting the line segment $\{x+iy\in {\mathbb {H}}:0<x<1\}$ is that $p/q\in \Gamma \mathfrak {c}\cap (-\tfrac{1}{2Y}, 1+\tfrac{1}{2Y})$ and $1/(q^2Y)>y$, i.e. $q^2<1/(yY)$. Thus there are only finitely many such horodiscs intersecting $\{x+iy\in {\mathbb {H}}:0<x<1\}$. Moreover, each such intersection is an open interval and the set $I_{y,Y,\mathfrak {c}}\subset (0,1)$ is thus the disjoint union of these finitely many open intervals. Similarly, for any $Y'>Y>1$ the set $\left\{ x\in (0,1):\Gamma (x+iy)\in {\mathcal {C}}_{Y,Y'}^{\Gamma ,\mathfrak {c}}\right\} =I_{y,Y,\mathfrak {c}}{\setminus } {\overline{I}}_{y,Y',\mathfrak {c}}$ is also a disjoint union of finitely many open intervals.

### Left regular action of normalizing elements

Let $h\in {\text {SL}}_2({\mathbb {Q}})$ be a group element normalizing $\Gamma$. The action of *h* on ${\mathbb {Q}}\cup \{\infty \}$ (by Möbius transformation) induces a well-defined action on $\Gamma \backslash \left( {\mathbb {Q}}\cup \{\infty \}\right)$, the set of cusps of $\Gamma$.

#### Lemma 6.3

For each $\mathfrak {c}\in \Omega _{\Gamma }$, we have

6.4 $$\begin{aligned} h\Gamma _{\mathfrak {c}} h^{-1}=\Gamma _{h \mathfrak {c}} \end{aligned}$$

and

6.5 $$\begin{aligned} \tau _{h \mathfrak {c}}^{-1}h\tau _{\mathfrak {c}}=\begin{pmatrix} \sqrt{\omega _{h\mathfrak {c}}/\omega _{\mathfrak {c}}} &{} *\\ 0 &{} \sqrt{\omega _{\mathfrak {c}}/\omega _{h\mathfrak {c}}}\end{pmatrix}\in {\text {SL}}_2({\mathbb {Q}}). \end{aligned}$$

#### Proof

Since *h* normalizes $\Gamma$ we have $h\Gamma _{\mathfrak {c}} h^{-1}=h\tau _{\mathfrak {c}}N\tau _{\mathfrak {c}}^{-1}h^{-1}\cap \Gamma$. Thus to prove (6.4) it suffices to show $h\tau _{\mathfrak {c}}N\tau _{\mathfrak {c}}^{-1}h^{-1}=\tau _{h \mathfrak {c}}N\tau _{h \mathfrak {c}}^{-1}$. We show that $\tau _{h \mathfrak {c}}^{-1}h\tau _{\mathfrak {c}}$ is an upper triangular matrix. Indeed, $\tau _{h \mathfrak {c}}^{-1}h\tau _{\mathfrak {c}}\infty =\tau _{h\mathfrak {c}}^{-1}\left( h\mathfrak {c}\right) =\infty$. This proves (6.4). We moreover conclude that

6.6 $$\begin{aligned} \tau _{h \mathfrak {c}}^{-1}h\tau _{\mathfrak {c}}=\begin{pmatrix} \lambda &{} *\\ 0 &{} \lambda ^{-1}\end{pmatrix} \end{aligned}$$

for some $\lambda \ne 0$, and it remains to show that $\lambda ^2=\omega _{h\mathfrak {c}}/\omega _{\mathfrak {c}}$. For this we conjugate the subgroup $\tau _{h\mathfrak {c}}^{-1}\Gamma _{h\mathfrak {c}}\tau _{h\cdot \mathfrak {c}}$ by the matrix $\tau _{h \mathfrak {c}}^{-1}h\tau _{\mathfrak {c}}$. We obtain with (6.4) that

$$\begin{aligned} \tau _{\mathfrak {c}}^{-1}h^{-1}\tau _{h\mathfrak {c}}\left( \tau _{h\mathfrak {c}}^{-1}\Gamma _{h\mathfrak {c}}\tau _{h\mathfrak {c}}\right) \tau _{h\mathfrak {c}}^{-1}h\tau _{\mathfrak {c}}=\tau _{\mathfrak {c}}^{-1}\Gamma _{\mathfrak {c}}\tau _{\mathfrak {c}}=\left\langle u_{\omega _{\mathfrak {c}}}\right\rangle . \end{aligned}$$

On the other hand, using (6.6) and $\tau _{h\mathfrak {c}}^{-1}\Gamma _{h \mathfrak {c}}\tau _{h\mathfrak {c}}=\left\langle u_{\omega _{h\mathfrak {c}}} \right\rangle$, we have

$$\begin{aligned}\tau _{\mathfrak {c}}^{-1}h^{-1}\tau _{h \mathfrak {c}}\left( \tau _{h\mathfrak {c}}^{-1}\Gamma _{h \mathfrak {c}}\tau _{h\mathfrak {c}}\right) \tau _{h\mathfrak {c}}^{-1}h\tau _{\mathfrak {c}}= \left( {\begin{matrix} \lambda ^{-1} &{}*\\ 0&{}\lambda \end{matrix}}\right) \left\langle \left( {\begin{matrix} 1 &{} \omega _{h\mathfrak {c}}\\ 0 &{} 1\end{matrix}}\right) \right\rangle \left( {\begin{matrix} \lambda &{}*\\ 0&{}\lambda ^{-1}\end{matrix}}\right) =\left\langle \left( {\begin{matrix} 1 &{} \omega _{h\mathfrak {c}}/\lambda ^2\\ 0 &{} 1\end{matrix}}\right) \right\rangle . \end{aligned}$$

Comparing both equations we conclude that $\lambda ^2=\omega _{h\mathfrak {c}}/\omega _{\mathfrak {c}}$. Finally replacing $\tau _{h\mathfrak {c}}$ with $-\tau _{h\mathfrak {c}}$ if necessary, we can ensure $\lambda$ is positive. $\square$

#### Proposition 6.4

Let $Y'>Y>0$ and $\mathfrak {c}\in \Omega _{\Gamma }$. If $\Gamma z\in {\mathcal {C}}^{\Gamma ,\mathfrak {c}}_{\omega _{\mathfrak {c}}Y, \omega _{\mathfrak {c}}Y'}$, then $\Gamma hz\in {\mathcal {C}}^{\Gamma ,h\mathfrak {c}}_{\omega _{h\mathfrak {c}}Y, \omega _{h\mathfrak {c}}Y'}$. Similarly, if $\Gamma z\in {\mathcal {C}}^{\Gamma ,\mathfrak {c}}_{\omega _{\mathfrak {c}}Y}$, then $\Gamma hz\in {\mathcal {C}}^{\Gamma ,h\mathfrak {c}}_{\omega _{h\mathfrak {c}}Y}$.

#### Proof

The second statement follows from the first one by taking $Y'\rightarrow \infty$. Since $\Gamma z\in {\mathcal {C}}^{\Gamma ,\mathfrak {c}}_{\omega _{\mathfrak {c}}Y, \omega _{\mathfrak {c}}Y'}$, by definition there exists $z'=x'+iy'\in {\mathbb {H}}$ with $0\le x'<\omega _\mathfrak {c}$ and $\omega _{\mathfrak {c}}Y<y'< \omega _{\mathfrak {c}}Y'$ and $\Gamma z=\Gamma \tau _{\mathfrak {c}}z'$. Consider $h\tau _\mathfrak {c}z'=\tau _{h\mathfrak {c}}z''$ with $z''=\tau _{h\mathfrak {c}}^{-1}h\tau _{\mathfrak {c}} z'$. By (6.5), we have ${\mathfrak {Im}}(z'') = (\omega _{h\mathfrak {c}}/\omega _\mathfrak {c}){\mathfrak {Im}}(z')\in (\omega _{h\mathfrak {c}}Y, \omega _{h\mathfrak {c}}Y')$, implying that $\Gamma hz=\Gamma h\tau _\mathfrak {c}z' \in {\mathcal {C}}_{\omega _{h\mathfrak {c}}Y,\omega _{h\mathfrak {c}}Y'}^{\Gamma ,h\mathfrak {c}}$. $\square$

## Negative results: horocycles expanding arbitrarily fast

In this section using the results from the previous section, we prove Theorems 1.7 and  1.8 which provide new limiting measures for the sequences $\left\{ \delta _{n,x,y_n}\right\} _{n\in {\mathbb {N}}}$ and $\left\{ \delta ^{\mathrm{pr}}_{n,x,y_n}\right\} _{n\in {\mathbb {N}}}$, allowing $\{y_n\}_{n\in {\mathbb {N}}}$ to decay arbitrarily fast. For any $n\in {\mathbb {N}}$ we consider the congruence subgroup $\Gamma _n< {\text {SL}}_2({\mathbb {Z}})$ given by

7.1 $$\begin{aligned} \Gamma _n:=\left\{ \begin{pmatrix} a &{} b\\ c &{} d\end{pmatrix}\in {\text {SL}}_2({\mathbb {Z}}):n^2\mid c,\ a \equiv d\equiv \pm 1 \ (\mathrm {mod}\ n)\right\} . \end{aligned}$$

It is clear that $\Gamma _1={\text {SL}}_2({\mathbb {Z}})$ and that $\Gamma _n$ contains the congruence subgroup

$$\begin{aligned} \Gamma _1(n^2):=\left\{ \gamma \in {\text {SL}}_2({\mathbb {Z}}):\gamma \equiv \begin{pmatrix} 1 &{} * \\ 0 &{} 1\end{pmatrix} \ (\mathrm {mod}\ n^2)\right\} . \end{aligned}$$

### Basic properties of the congruence subgroups $\Gamma _n$

First we show that $\Gamma _n$ is normalized by $u_{j/n}$ for any $j\in {\mathbb {Z}}$. As mentioned in the introduction this simple fact is the starting point of our proofs to Theorems 1.7 and  1.8.

#### Lemma 7.1

For any $n\in {\mathbb {N}}$ and for any $j\in {\mathbb {Z}}$, the unipotent matrix $u_{j/n}$ normalizes $\Gamma _n$.

#### Proof

By direct computation, for any $$\gamma =\left( {\begin{matrix} a &{} b\\ c&{} d\end{matrix}}\right) \in \Gamma _1$$ and for any $j\in {\mathbb {Z}}$ we have

$$\begin{aligned} u^{-1}_{j/n}\gamma u_{j/n}=\begin{pmatrix} a-\frac{jc}{n} &{} b+\frac{(a-d)j}{n}-\frac{j^2c}{n^2}\\ c &{} d+\frac{jc}{n}\end{pmatrix}. \end{aligned}$$

Hence if $\gamma \in \Gamma _n$, that is, $n^2\mid c$ and $a\equiv d\equiv \pm 1 \ (\mathrm {mod}\ n)$, all the entries are integers with the bottom left entry divisible by $n^2$, and

$$\begin{aligned} a-\frac{jc}{n}\equiv a\equiv d\equiv d+\frac{jc}{n}\equiv \pm 1\ \ (\mathrm {mod}\ n). \end{aligned}$$

This implies that $u^{-1}_{j/n}\Gamma _n u_{j/n}\subset \Gamma _n$. $\square$

Next we prove the following index formula for $\Gamma _n$.

#### Lemma 7.2

For any integer $n\ge 3$, we have

7.2 $$\begin{aligned}{}[\Gamma _1: \Gamma _n]=\frac{n^3}{2}\prod _{\begin{array}{c} p | n\\ \text {prime} \end{array}}\left( 1-p^{-2}\right) . \end{aligned}$$

#### Proof

Let $J_n< \left( {\mathbb {Z}}/n^2{\mathbb {Z}}\right) ^{\times }$ be the subgroup

7.3 $$\begin{aligned} J_n:= \left\{ [a]\in \left( {\mathbb {Z}}/n^2{\mathbb {Z}}\right) ^{\times }:a\equiv \pm 1 \ (\mathrm {mod}\ n)\right\} . \end{aligned}$$

It is easy to check that $\#(J_n)=2n$. Consider the map $h: \Gamma _n \rightarrow J_n$ sending $$\gamma =\left( {\begin{matrix} a &{} b\\ c &{} d\end{matrix}}\right) \in \Gamma _n$$ to $[a]\in \left( {\mathbb {Z}}/n^2{\mathbb {Z}}\right) ^{\times }$. Using the definition of $\Gamma _n$, one can check that *h* is a group homomorphism with the kernel $\ker (h)=\Gamma _1(n^2)$. For each $0\le k\le n-1$, set $$\gamma _k^{\pm }=\pm \left( {\begin{matrix} 1+kn &{} 1\\ -k^2n^2 &{} 1-kn\end{matrix}}\right) \in \Gamma _n$$. Then *h* surjects the set $\left\{ \gamma _k^{\pm }\in \Gamma _n:0\le k\le n-1\right\}$ onto $J_n$. Finally we use the index formula for $\Gamma _1(n^2)$ (see e.g. [6, Section 1.2]) to get

$$\begin{aligned} {[}\Gamma _1 : \Gamma _n]=\frac{[\Gamma _1 : \Gamma _1(n^2)]}{[\Gamma _n : \Gamma _1(n^2)]}=\frac{[\Gamma _1 : \Gamma _1(n^2)]}{\# J_n}=\frac{n^3}{2}\prod _{\begin{array}{c} p | n\\ \text {prime} \end{array}}\left( 1-p^{-2}\right) . \end{aligned}$$

$$\square $$

Next, we study the properties of $\Gamma _n$ relative to its cusps. As in Sect. 6 we denote by $\Omega _{\Gamma _n}$ the set of cusps of $\Gamma _n$. The following lemma computes the width of each cusp of $\Gamma _n$.

#### Lemma 7.3

Let $n\in {\mathbb {N}}$ and let $\mathfrak {c}=m/l\in \Omega _{\Gamma _n}$ with $\gcd (m,l)=1$ (if $\mathfrak {c}=\infty$, *m*/*l* is understood as 1/0). Then we have

$$\begin{aligned} \omega _{\mathfrak {c}}=\frac{n^2}{\gcd (n,l)^2}. \end{aligned}$$

#### Proof

Let $\tau _{\mathfrak {c}}\in \Gamma _1$ be as before such that $\tau _{\mathfrak {c}}\infty =\mathfrak {c}$. Thus the left column of $\tau _{\mathfrak {c}}$ is $$\left( {\begin{matrix} m \\ l\end{matrix}}\right) $$. By direct computation we have

$$\begin{aligned} \tau _{\mathfrak {c}}N\tau _{\mathfrak {c}}^{-1}=\left\{ \begin{pmatrix} 1-ml t&{} m^2t\\ -l^2t &{} 1+mlt\end{pmatrix}\in G:t\in {\mathbb {R}}\right\} . \end{aligned}$$

Thus by (7.1) an element in $(\Gamma _n)_{\mathfrak {c}}=\tau _{\mathfrak {c}}N\tau _{\mathfrak {c}}^{-1}\cap \Gamma _n$ is of the form $$\gamma =\left( {\begin{matrix} 1-ml t&{} m^2t\\ -l^2t &{} 1+mlt\end{matrix}}\right) \in \Gamma _1$$ satisfying that $n^2\mid l^2 t$ and $1- mlt\equiv 1+mlt\equiv \pm 1\ (\mathrm {mod}\ n)$. Looking at the top right and bottom left entries of $\gamma$, we have that $m^2 t, l^2 t\in {\mathbb {Z}}$. Since $\gcd (m,l)=1$, we have $t\in {\mathbb {Z}}$. Then the condition $n^2\mid l^2t$ is equivalent to $\frac{n^2}{\gcd (n,l)^2}\mid t$, and the condition $n\mid mlt$ is equivalent to that $\frac{n}{\gcd (n,ml)}\mid t$. Moreover, since $\frac{n}{\gcd (n,ml)}\mid \frac{n^2}{\gcd (n,l)^2}$, the condition $\frac{n}{\gcd (n,ml)}\mid t$ is implied by the condition $\frac{n^2}{\gcd (n,l)^2}\mid t$. We conclude that $n^2\mid l^2 t$ implies $1-mlt\equiv 1+mlt\equiv \pm 1\ (\mathrm {mod}\ n)$. Thus

$$\begin{aligned} (\Gamma _n)_{\mathfrak {c}}= \left\{ \begin{pmatrix} 1-ml t&{} m^2t\\ -l^2t &{} 1+ mlt\end{pmatrix}\in \Gamma _1: n^2\mid l^2t\right\} . \end{aligned}$$

Conjugating $(\Gamma _n)_{\mathfrak {c}}$ back via $\tau _{\mathfrak {c}}$ and using the equivalence of the two conditions $n^2 \mid l^2 t$ and $\frac{n^2}{\gcd (n,l)^2} \mid t$ we get

$$\begin{aligned} \tau _{\mathfrak {c}}^{-1}(\Gamma _n)_{\mathfrak {c}}\tau _{\mathfrak {c}}=\left\{ u_t=\begin{pmatrix} 1 &{} t\\ 0 &{} 1\end{pmatrix}\in \Gamma _1:\frac{n^2}{\gcd (n,l)^2}\mid t\right\} , \end{aligned}$$

implying that $$\omega _{\mathfrak {c}}=n^2/\gcd (n,l)^2$$. $$\square $$

Next we compute the number of cusps of $\Gamma _n$.

#### Proposition 7.4

For any integer $n\ge 3$ we have

$$\begin{aligned} \#\Omega _{\Gamma _n}=\frac{n^2}{2}\prod _{\begin{array}{c} p | n\\ \text {prime} \end{array}}\left( 1-p^{-2}\right) . \end{aligned}$$

#### Remark 7.4

It is easy to check that $\Gamma _2=\Gamma _0(4)$. Thus $[\Gamma _1: \Gamma _2]=6$ and $\Gamma _2$ has three cusps which can be represented by $\infty$, 1/2 and 1 respectively.

To prove Proposition 7.4 we first prove a preliminary formula for $\#\Omega _{\Gamma _n}$.

#### Lemma 7.5

For any integer $n\ge 3$ we have

$$\begin{aligned} \#\Omega _{\Gamma _n}=\sum _{d | n^2}\frac{\varphi (n^2/d)\varphi (d)\gcd (n^2/d,d)}{2n}. \end{aligned}$$

#### Proof

Since $-I_2\in \Gamma _n$ and $\Gamma _1(n^2) < \Gamma _n$, we have $\Omega _{\Gamma _n}=\Gamma _n\backslash \Omega _{\Gamma _1(n^2)}$. On the other hand, by the analysis in [6, p. 102], the set $\Omega _{\Gamma _1(n^2)}$ is in bijection with the union of cosets $\bigsqcup _{d| n^2}\langle \pm I_2\rangle \backslash Z_d$, where for each $d\mid n^2$,

$$\begin{aligned} Z_d:=\left\{ ([m],[l])^t:[m]\in \left( {\mathbb {Z}}/d{\mathbb {Z}}\right) ^{\times }, [l]\in {\mathbb {Z}}/n^2{\mathbb {Z}}, \gcd (n^2, l)=d\right\} \end{aligned}$$

with $([m],[l])^t$ is the transpose of the row vector ([*m*], [*l*]) and the bijection is induced by the map sending $m/l\in {\mathbb {Q}}\cup \{\infty \}$ with $\gcd (m,l)=1$ to $([m],[l]))^t\in Z_{d}$ with $d=\gcd (n^2,l)$. Note that $\# Z_d=\varphi (n^2/d)\varphi (d)$.

For each $d\mid n^2$, using the definition of $\Gamma _n$, it is easy to check that the linear action of $\Gamma _n$ on ${\mathbb {Z}}^2$ (by matrix multiplication) induces a well-defined action of $\Gamma _n$ on $Z_d$ and that the corresponding action of the subgroup $\Gamma _1(n^2)$ is trivial. From the proof of Lemma 7.2, we have $\Gamma _n/\Gamma _1(n^2)\cong J_n$, where

7.5 $$\begin{aligned} J_n=\left\{ \pm [1+kn]\in ({\mathbb {Z}}/n^2{\mathbb {Z}})^{\times }:0\le k\le n-1\right\} , \end{aligned}$$

which is of size 2*n*. Hence the action of $\Gamma _n$ on $Z_d$ induces the action of $J_n$ on $Z_d$ given by

$$\begin{aligned}{}[a]\cdot ([m], [l])^t=([am], [{\overline{a}}l])^t, \end{aligned}$$

with $([m],[l])^t\in Z_d$ and ${\overline{a}}$ the multiplicative inverse of *a* modulo $n^2$. We note that $[am]\in \left( {\mathbb {Z}}/d{\mathbb {Z}}\right) ^{\times }$ is well-defined since $d\mid n^2$.

We conclude that $\Omega _{\Gamma _n}=\Gamma _n\backslash \Omega _{\Gamma _1(n^2)}$ is in bijection with the union of cosets

$$\begin{aligned} \bigsqcup _{d| n^2}\Gamma _n\backslash Z_d=\bigsqcup _{d| n^2}J_n\backslash Z_d, \end{aligned}$$

implying that

$$\begin{aligned} \#\Omega _{\Gamma _n}=\sum _{d| n^2}\# J_n\backslash Z_d. \end{aligned}$$

Hence we want to compute the size of the coset $J_n\backslash Z_d$ for each $d\mid n^2$. For this we claim that for any for any $([m] ,[l])^t\in Z_d$, the orbit $J_n\cdot ([m],[l])^t$ is of size $2n/\gcd (n^2/d,d)$, implying that

$$\begin{aligned} \#J_n\backslash Z_d=\frac{\#Z_d}{2n/\gcd (n^2/d,d)}=\frac{\varphi (n^2/d)\varphi (d)\gcd (n^2/d,d)}{2n}. \end{aligned}$$

We note that Lemma 7.5 then follows immediately from this claim. To prove this claim, it suffices to compute the size of the stabilizer

$$\begin{aligned} (J_n)_{([m],[l])}:=\left\{ [a]\in J_n:[a]\cdot ([m],[l])^t= ([m], [l])^t\in Z_d\right\} . \end{aligned}$$

Since by definition $[a]\cdot ([m],[l])^t=([am], [{\overline{a}}l])^t$, $[a]\in (J_n)_{([m],[l])}$ if and only if $am\equiv m \ (\mathrm {mod}\ d)$ and ${\overline{a}}l\equiv l\ (\mathrm {mod}\ n^2)$. Since $d=\gcd (n^2,l)$ and $[m]\in \left( {\mathbb {Z}}/d{\mathbb {Z}}\right) ^{\times }$, these two conditions are equivalent to $a\equiv 1 \ (\mathrm {mod}\ d)$ and ${\overline{a}}\equiv 1\ (\mathrm {mod}\ n^2/d)$, which are equivalent to $a\equiv 1\ (\mathrm {mod}\ {\text {lcm}}(n^2/d, d))$. Hence using the description (7.5) of $J_n$ and the facts that $n\mid {\text {lcm}}(n^2/d, d)$ and ${\text {lcm}}(n^2/d, d)\gcd (n^2/d, d)=n^2$ we have

$$\begin{aligned} (J_n)_{([m],[l])}=\left\{ [1+{\text {lcm}}(n^2/d, d) j]\in J_n:0\le j\le \gcd (n^2/d, d)-1\right\} \end{aligned}$$

is of size $\gcd (n^2/d, d)$. This implies that

$$\begin{aligned} \#\left( J_n\cdot ([m],[l])^t\right) =\frac{\#J_n}{\#(J_n)_{([m],[l])}}=\frac{2n}{\gcd (n^2/d,d)}, \end{aligned}$$

proving the claim, and hence also this lemma. $\square$

We can now give the proof of Proposition 7.4 by simplifying the formula in Lemma 7.5.

#### Proof of Proposition 7.4

Write $n=\prod _{i=1}^k p_i^{\alpha _i}$ in the prime decomposition form and apply Lemma 7.5 to get

$$\begin{aligned} \#\left( \Omega _{\Gamma _n}\right)&=\frac{1}{2n}\sum _{\varvec{\beta }\in {\mathbb {Z}}^k: 0\le \beta _i\le 2\alpha _i}\varphi \left( \prod _{i=1}^kp_i^{\beta _i}\right) \varphi \left( \prod _{i=1}^kp_i^{2\alpha _i-\beta _i}\right) \prod _{i=1}^kp_i^{\min \{\beta _i, 2\alpha _i-\beta _i\}}, \end{aligned}$$

where the summation is over all vectors $\varvec{\beta }=(\beta _1,\ldots , \beta _k)\in {\mathbb {Z}}^k$ satisfying $0\le \beta _i\le 2\alpha _i$ for all $1\le i\le k$, and we used that $\gcd (n^2/d, d)=\prod _{i=1}^kp_i^{{\min \{\beta _i, 2\alpha _i-\beta _i\}}}$ for $d=\prod _{i=1}^k p_i^{\beta _i}$. Using the fact that $\varphi$ is multiplicative and interchanging the summation and product signs we get

$$\begin{aligned} \#\left( \Omega _{\Gamma _n}\right)&=\frac{1}{2n}\prod _{i=1}^k\left( \sum _{0\le \beta _i\le 2\alpha _i}\varphi \left( p_i^{\beta _i}\right) \varphi \left( p_i^{2\alpha _i-\beta _i}\right) p_i^{\min \{\beta _i, 2\alpha _i-\beta _i\}}\right) \\&=\frac{1}{2n}\prod _{i=1}^k\left( \sum _{1\le \beta _i\le 2\alpha _i-1}p_i^{2\alpha _i}(1-p_i^{-1})^2p_i^{\min \{\beta _i, 2\alpha _i-\beta _i\}}+2p_i^{2\alpha _i}(1-p_i^{-1})\right) \\&=\frac{1}{2n}\prod _{i=1}^kp_i^{2\alpha _i}(1-p_i^{-1})\left( (1-p_i^{-1})\sum _{1\le \beta _i\le 2\alpha _i-1}p_i^{\min \{\beta _i, 2\alpha _i-\beta _i\}}+2\right) , \end{aligned}$$

where for the second equality we used that for $1\le \beta _i\le 2\alpha _i-1$, $\varphi \left( p_i^{\beta _i}\right) \varphi \left( p_i^{2\alpha _i-\beta _i}\right) =p^{2\alpha _i}(1-p_i^{-1})^2$, and for $\beta _i=0$ or $\beta _i=2\alpha _i$, $\varphi \left( p_i^{\beta _i}\right) \varphi \left( p_i^{2\alpha _i-\beta _i}\right) =p^{2\alpha _i}(1-p_i^{-1})$ and $\min \{\beta _i, 2\alpha _i-\beta _i\}=0$. We note that the term $\sum _{1\le \beta _i\le 2\alpha _i-1}p_i^{\min \{\beta _i, 2\alpha _i-\beta _i\}}$ equals

$$\begin{aligned}&\sum _{1\le \beta _i\le \alpha _i}p_i^{\beta _i}+\sum _{\alpha _i< \beta _i\le 2\alpha _i-1}p_i^{2\alpha _i-\beta _i}=\sum _{1\le \beta _i\le \alpha _i}p_i^{\beta _i}+\sum _{1\le \beta _i< \alpha _i}p_i^{\beta _i}\\&\quad =2\sum _{1\le \beta _i\le \alpha _i}p_i^{\beta _i}-p_i^{\alpha _i}=\frac{2p_i(p_i^{\alpha _i}-1)}{p_i-1}-p_i^{\alpha _i}. \end{aligned}$$

Hence we have

$$\begin{aligned} \#\left( \Omega _{\Gamma _n}\right)&=\frac{1}{2n}\prod _{i=1}^kp_i^{2\alpha _i}(1-p_i^{-1})\left( (1-p_i^{-1})\left( \frac{2p_i(p_i^{\alpha _i}-1)}{p_i-1}-p_i^{\alpha _i}\right) +2\right) \\&=\frac{1}{2n}\prod _{i=1}^kp_i^{2\alpha _i}(1-p_i^{-1})p_i^{\alpha _i}(1+p_i^{-1})=\frac{n^2}{2}\prod _{i=1}^k(1-p_i^{-2}), \end{aligned}$$

finishing the proof. $\square$

### Proof of Theorem 1.7

For simplicity of notation, we abbreviate the cusp neighborhoods ${\mathcal {C}}_Y^{\Gamma _n,\mathfrak {c}}$ and ${\mathcal {C}}_{Y,Y'}^{\Gamma _n,\mathfrak {c}}$ by ${\mathcal {C}}_Y^{n,\mathfrak {c}}$ and ${\mathcal {C}}_{Y,Y'}^{n,\mathfrak {c}}$ respectively and the set of cusps $\Omega _{\Gamma _n}$ by $\Omega _n$. We first prove the following key lemma which says that if $\Gamma _n z$ visits a cusp neighborhood on $\Gamma _n\backslash {\mathbb {H}}$, then all companion points $\Gamma _1 u_{j/n} z, 0\le j\le n-1$ make excursions to some cusp neighborhood on ${\mathcal {M}}=\Gamma _1\backslash {\mathbb {H}}$, the modular surface. We recall that ${\mathcal {C}}_Y$ is the projection onto ${\mathcal {M}}$ of the region $\{z\in {\mathbb {H}}:{\mathfrak {Im}}(z)>Y\}$.

#### Lemma 7.6

Let $Y>0$ and $n\in {\mathbb {N}}$. If $\Gamma _n z\in {\mathcal {C}}_{\omega _{\mathfrak {c}}Y}^{n,\mathfrak {c}}$ for some $\mathfrak {c}\in \Omega _{n}$ then $\Gamma _1 u_{j/n}z\in {\mathcal {C}}_Y$ for all $0\le j\le n-1$.

#### Proof

Fix $0\le j\le n-1$. By Lemma 7.1, $u_{j/n}$ normalizes $\Gamma _n$. Assuming that $\Gamma _n z\in {\mathcal {C}}_{\omega _{\mathfrak {c}}Y}^{n,\mathfrak {c}}$ and applying Proposition 6.4 to $h=u_{j/n}$, we get $\Gamma _n u_{j/n}z\in {\mathcal {C}}_{\omega _{h\mathfrak {c}}Y}^{n,h\mathfrak {c}}$. By definition, there exists $z'\in {\mathbb {H}}$ with ${\mathfrak {Im}}(z')>\omega _{h\mathfrak {c}}Y\ge Y$ such that $\Gamma _n \tau _{h\mathfrak {c}} z' = \Gamma _n u_{j/n} z$. Since $\tau _{h\mathfrak {c}}\in \Gamma _1$, this implies $\Gamma _1 u_{j/n}z = \Gamma _1 z'\in {\mathcal {C}}_Y$. $\square$

We can now give the

#### Proof of Theorem 1.7

For any $n\in {\mathbb {N}}$ let $Y_n=\max \{\log n, 1\}$, and let $\Psi _n$ be the indicator function of the union

$$\begin{aligned} \bigcup _{\mathfrak {c}\in \Omega _n}{\mathcal {C}}_{\omega _{\mathfrak {c}}Y_n,2\omega _{\mathfrak {c}}Y_n}^{n,\mathfrak {c}}\subset \Gamma _n\backslash {\mathbb {H}}. \end{aligned}$$

Since for any cusp $\mathfrak {c}\in \Omega _{n}$, $\omega _{\mathfrak {c}}Y_n\ge Y_n\ge 1$, by Lemma 6.1, each ${\mathcal {C}}_{\omega _{\mathfrak {c}}Y_n,2\omega _{\mathfrak {c}}Y_n}^{n,\mathfrak {c}}$ is a Borel set with boundary of measure zero; and by Lemma 6.2 the above union is disjoint. Thus $\Psi _n$ is the indicator function of a Borel set with boundary of measure zero. Moreover, applying the volume formula (6.2), the index formula in Lemma 7.2 and the cusp number formula in Proposition 7.4 (see also Remark 7.4 for the case when $n=2$) we have for any $n\in {\mathbb {N}}$,

7.6 $$\begin{aligned} \mu _{\Gamma _n}\left( \Psi _n\right)&=\sum _{\mathfrak {c}\in \Omega _n}\mu _{\Gamma _n}\left( {\mathcal {C}}_{\omega _{\mathfrak {c}}Y_n,2\omega _{\mathfrak {c}}Y_n}^{n,\mathfrak {c}}\right) \nonumber \\&=\sum _{\mathfrak {c}\in \Omega _n}\frac{3\omega _{\mathfrak {c}}}{\pi [\Gamma _1 : \Gamma _n]}\times \frac{1}{2\omega _{\mathfrak {c}}Y_n}=\frac{3}{2\pi Y_n}\frac{\# \Omega _n}{[\Gamma _1 : \Gamma _n]}\asymp \frac{1}{nY_n}. \end{aligned}$$

For any $n\in {\mathbb {N}}$ and $0<y<1$ we define

$$\begin{aligned} I_n(y):=\left\{ x\in {\mathbb {R}}/{\mathbb {Z}}:\Psi _n(x+iy)=1\right\} . \end{aligned}$$

By definition, $x\in I_n(y)$ if and only if $\Gamma _n(x+iy)\in {\mathcal {C}}_{\omega _{\mathfrak {c}}Y_n,2\omega _{\mathfrak {c}}Y_n}^{n,\mathfrak {c}}\subset {\mathcal {C}}_{\omega _{\mathfrak {c}}Y_n}^{n,\mathfrak {c}}$ for some $\mathfrak {c}\in \Omega _n$. Thus Lemma 7.6 implies that

$$\begin{aligned} I_n(y) \subset \{x\in {\mathbb {R}}/{\mathbb {Z}}: {\mathcal {R}}_n(x,y)\subset {\mathcal {C}}_{Y_n}\}. \end{aligned}$$

This, together with our choice that $Y_n=\max \{\log n, 1\}$ and the distance formula (4.1), implies that for any $n\ge 3$ and for any $x\in I_n(y)$

$$\begin{aligned} \inf _{\Gamma _1 z\in {\mathcal {R}}_n(x, y)}d_{{\mathcal {M}}}(\Gamma _1 z_0,\Gamma _1 z)\ge \log (Y_n)+O(1)=\log \log n+ O(1). \end{aligned}$$

It thus suffices to show that there exists a sequence $\{y_n\}_{n\in {\mathbb {N}}}$ satisfying that $0<y_n<c_n$ for all $n\in {\mathbb {N}}$ and that the limsup set $\mathop {{\overline{\lim }}}_{n\rightarrow \infty }I_n(y_n)$ is of full Lebesgue measure in ${\mathbb {R}}/{\mathbb {Z}}$.

For this, we will construct a sequence $\{y_n\}_{n\in {\mathbb {N}}}$ decaying sufficiently fast and then apply the quantitative Borel–Cantelli lemma Corollary 2.6 to the sequence $\{I_n(y_n)\}_{n\in {\mathbb {N}}}\subset {\mathbb {R}}/{\mathbb {Z}}$. To ensure the quasi-independence condition (2.20) in Corollary 2.6, we need, for every pair $1\le m<n\in {\mathbb {N}}$, the two quantities $\left| I_m(y_m)\cap I_n(y_n)\right|$ and $\left| I_m(y_m)\right| \left| I_n(y_n)\right|$ to be sufficiently close to each other. The key observations for this are the following two relations that

7.7 $$\begin{aligned} \left| I_n(y_n)\right| =\int _0^1\Psi _n(x+iy_n)dx \end{aligned}$$

and

7.8 $$\begin{aligned} \left| I_{m}(y_m)\cap I_n(y_n)\right| =\int _0^1\Psi _{m}(x+iy_m)\Psi _n(x+iy_n) dx=\int _{I_{m}(y_m)}\Psi _n(x+iy_n) dx.\nonumber \\ \end{aligned}$$

Assuming the limit equation (2.16) holds for the pairs $((0,1), \Psi _n)$ and $(I_m(y_m), \Psi _n)$ (we will verify this later), then by relation (7.8) the quantity $\left| I_{m}(y_m)\cap I_n(y_n)\right|$ is close to the quantity $|I_m(y_m)|\mu _{\Gamma _n}(\Psi _n)$ which in turn is close to $|I_m(y_m)||I_n(y_n)|$ by relation (7.7), provided that $y_n>0$ is sufficiently small.

We now implement the above ideas rigorously. We first claim that there exists a sequence $\{y_n\}_{n\in {\mathbb {N}}}$ satisfying, for all $n\in {\mathbb {N}}$, $0<y_n<c_n$ and

7.9 $$\begin{aligned} \left| \frac{1}{\left| I\right| }\int _{I}\Psi _n(x+iy_n)dx-\mu _{\Gamma _n}(\Psi _n)\right| \le \frac{\mu _{\Gamma _n}(\Psi _n) }{2n^2}, \end{aligned}$$

for any subset $I\subset {\mathbb {R}}/{\mathbb {Z}}$ taken from the finite set

7.10 $$\begin{aligned} \left\{ (0,1)\right\} \bigcup \left\{ I_{m}(y_{m}): 1\le m<n\right\} . \end{aligned}$$

For this, first note that by Remark 2.17 for any $I\subset {\mathbb {R}}/{\mathbb {Z}}\cong [0,1)$ a disjoint union of finitely many open intervals, we have

7.11 $$\begin{aligned} \lim \limits _{y\rightarrow 0^+}\frac{1}{|I|}\int _{I}\Psi _n(x+iy)dx= \mu _{\Gamma _n}(\Psi _n). \end{aligned}$$

We now construct such a sequence successively. For the base case $n=1$ since (7.11) holds for the pair $((0,1),\Psi _1)$ on ${\mathcal {M}}=\Gamma _1\backslash {\mathbb {H}}$, there exists $0<y_1<c_1$ sufficiently small such that

$$\begin{aligned} \left| \int _0^1\Psi _1(x+iy_1)dx- \mu _{\Gamma _1}(\Psi _1)\right| < \frac{1}{2}\mu _{\Gamma _1}(\Psi _1). \end{aligned}$$

For a general integer $n\ge 2$, suppose that we already have chosen $0<y_m<c_m$ satisfying (7.9) for all the positive integers $m< n$. By Remark 6.3 the set $I_m(y_m)\subset {\mathbb {R}}/{\mathbb {Z}}$ is a disjoint union of finitely many open intervals for any $m<n$. Thus (7.11) is satisfied for all the pairs

$$\begin{aligned} \left( (0,1), \Psi _n\right) , (I_m(y_m), \Psi _n), 1\le m< n \end{aligned}$$

on $\Gamma _n\backslash {\mathbb {H}}$. Since there are only finitely many such pairs, we can take $0<y_n<c_n$ sufficiently small such that (7.9) is satisfied for all $I\in \left\{ (0,1)\right\} \bigcup \left\{ I_{m}(y_{m}): 1\le m<n\right\}$, which is the set in (7.10). This finishes the proof of the claim.

Now let $\{y_n\}_{n\in {\mathbb {N}}}$ be as in the claim. For any $n\in {\mathbb {N}}$ apply (7.9) to the pair $((0,1),\Psi _n)$ we get

7.12 $$\begin{aligned} \left| \left| I_n(y_n)\right| -\mu _{\Gamma _n}(\Psi _n)\right| \le \frac{\mu _{\Gamma _n}(\Psi _n)}{2n^2}. \end{aligned}$$

By the triangle inequality, this implies

7.13 $$\begin{aligned} \mu _{\Gamma _n}(\Psi _n)\le \ 2|I_n(y_n)|. \end{aligned}$$

More generally, for each $1\le m< n$ apply (7.9) to the pair $(I_{m}(y_{m}),\Psi _n)$ we get

7.14 $$\begin{aligned} \left| \left| I_{m}(y_{m})\cap I_n(y_n)\right| -\left| I_{m}(y_{m})\right| \mu _{\Gamma _n}(\Psi _n)\right| \le \frac{\left| I_{m}(y_{m})\right| \mu _{\Gamma _n}(\Psi _n)}{2n^2}. \end{aligned}$$

Using the inequalities (7.12), (7.13), (7.14) together with the triangle inequality we get

7.15 $$\begin{aligned}&\left| \left| I_{m}(y_{m})\cap I_n(y_n)\right| -\left| I_{m}(y_{m})\right| \left| I_n(y_n)\right| \right| \le \frac{\left| I_{m}(y_{m})\right| \mu _{\Gamma _n}(\Psi _n)}{n^2}\nonumber \\&\quad \le \frac{2\left| I_{m}(y_{m})\right| \left| I_n(y_n)\right| }{n^2}. \end{aligned}$$

Hence the sequence $\left\{ I_n(y_n)\right\} _{n\in {\mathbb {N}}}\subset {\mathbb {R}}/{\mathbb {Z}}$ satisfies the quasi-independence condition (2.20) (with the subset ${\mathbb {S}}={\mathbb {N}}$ and the exponent $\eta =2$). Moreover, using the inequality (7.12), the volume computation (7.6) and the estimate that $Y_n\asymp \log n$ we have that

$$\begin{aligned} \sum _{n\in {\mathbb {N}}}\left| I_n(y_n)\right| \ge \sum _{n\in {\mathbb {N}}}\frac{1}{2}\mu _{\Gamma _n}(\Psi _n)\asymp \sum _{n\in {\mathbb {N}}}\frac{1}{n\log n}=\infty . \end{aligned}$$

Thus by Corollary 2.6, $\mathop {{\overline{\lim }}}_{n\rightarrow \infty }I_n(y_n)\subset {\mathbb {R}}/{\mathbb {Z}}$ is of full Lebesgue measure, finishing the proof. $\square$

#### Remark 7.16

It is not clear to us whether the rate $\log \log n$ is the fastest excursion rate for generic translates. We note that in principle it can be proved (or disproved) if one can compute the volume of the set

$$\begin{aligned} {\mathcal {E}}_Y^n:=\left\{ \Gamma _n z\in \Gamma _n\backslash {\mathbb {H}}:\Gamma _1 u_{j/n}z\in {\mathcal {C}}_Y\ \text {for all}\, 0 \le j\le n-1\right\} . \end{aligned}$$

For instance, if one can show $\mu _{\Gamma _n}({\mathcal {E}}_Y^n)\asymp 1/(nY)$ for all $n\in {\mathbb {N}}$ and for all $Y\ge 1$, then Theorem 1.7 together with a standard application of the Borel–Cantelli lemma would imply that the inequality in (1.13) is indeed an equality for almost every $x\in {\mathbb {R}}/{\mathbb {Z}}$. We also note that our analysis (Lemmas 6.2,  7.6) shows that for any $n\in {\mathbb {N}}$ and for any $Y\ge 1$

$$\begin{aligned} \bigsqcup _{\mathfrak {c}\in \Omega _n}{\mathcal {C}}_{\omega _{\mathfrak {c}}Y}^{n,\mathfrak {c}}\subset {\mathcal {E}}_Y^n\subset \bigsqcup _{\mathfrak {c}\in \Omega _n}{\mathcal {C}}_{Y}^{n,\mathfrak {c}}, \end{aligned}$$

implying that $1/(nY)\ll \mu _{\Gamma _n}\left( {\mathcal {E}}_Y^n\right) \ll 1/Y$. On the other hand using some elementary arguments (which relies on the width computation Lemma 7.3) one can show that any $\langle u_{1/n}\rangle$-orbit contains at least one cusp of width one. This fact together with the fact that $1\le \omega _{\mathfrak {c}}\le n^2$ implies that ${\mathcal {E}}_Y^n= \bigsqcup _{\mathfrak {c}\in \Omega _n}{\mathcal {C}}_{Y}^{n,\mathfrak {c}}$ when $Y\ge n^2$ . However, both estimates are not sufficient for the purpose of obtaining an upper bound.

#### Remark 7.17

Here we give a very brief sketch of the argument communicated to us by Strömbergsson: For each $n\in {\mathbb {N}}$ and $y>0$, it is not difficult to see that $\Gamma _n(x+iy)\in {\mathcal {C}}_{\omega _{\mathfrak {c}}Y_n}^{n,\mathfrak {c}}$ for some $\mathfrak {c}=\tfrac{p}{q}\in \Omega _n$ with $\gcd (p,q)=1$ if and only if

7.18 $$\begin{aligned} \left| x-\frac{p}{q}\right| ^2<\frac{y}{\omega _{\mathfrak {c}}Y_nq^2}-y^2=\frac{y\gcd (n,q)^2}{n^2Y_n q^2}-y^2. \end{aligned}$$

Here $Y_n=\max \{\log n, 1\}$ is as in the above proof. Define

$$\begin{aligned} {\tilde{I}}_n(y):=\left\{ x\in {\mathbb {R}}/{\mathbb {Z}}:\ \exists \,\text {primitive}\, \frac{p}{q} \,\text {s.t.}\,\, n\mid q, q<\frac{1}{2\sqrt{yY_n}}, \left| x-\frac{p}{q}\right| <\frac{\sqrt{y}}{2\sqrt{Y_n }q}\right\} . \end{aligned}$$

One can easily check that elements in ${\tilde{I}}_n(y)$ satisfy the inequality (7.18). Hence by Lemma 7.6 we have

7.19 $$\begin{aligned} {\tilde{I}}_n(y)\subset \{x\in {\mathbb {R}}/{\mathbb {Z}}: {\mathcal {R}}_n(x,y)\subset {\mathcal {C}}_{Y_n}\}. \end{aligned}$$

Moreover, using some standard techniques from analytic number theory one can show that for any subinterval $I\subset {\mathbb {R}}/{\mathbb {Z}}$ (or more generally, any finite disjoint union of subintervals),

$$\begin{aligned} \lim \limits _{y\rightarrow 0^+}|I|^{-1}\left| {\tilde{I}}_n(y)\cap I\right| =\frac{c_n}{Y_n} \end{aligned}$$

with $c_n=\frac{3}{\pi ^2}\frac{\varphi (n)}{n^2}\prod _{p\not \mid n}(1-p^{-2})^{-1}\gg \frac{\varphi (n)}{n^2}$. This limit equation is the analog of (7.11). Another input is the divergence of the series $\sum _{n\in {\mathbb {N}}}\frac{c_n}{Y_n}\gg \sum _{n\in {\mathbb {N}}}\frac{\varphi (n)}{n^2\log n}$, which follows from the estimate $\varphi (n)\gg n/\log \log n$. With these two inputs one can then mimic the arguments in the above proof to construct a sequence $\{y_n\}_{n\in {\mathbb {N}}}$ decaying sufficiently fast and then apply Corollary 2.6 to get a full measure limsup set $\mathop {{\overline{\lim }}}_{n\rightarrow \infty }{\tilde{I}}_n(y_n)\subset {\mathbb {R}}/{\mathbb {Z}}$. Finally, we note that the relation (7.19) can be checked directly using the definition of the set ${\tilde{I}}_n(y)$. Hence this argument can be carried over without going into the congruence covers $\Gamma _n\backslash {\mathbb {H}}$.

### Proof of Theorem 1.8

We prove Theorem 1.8 in this subsection. The strategy is similar to that of Theorem 1.7 with the sequence of cuspidal sets approaching the cusps replaced by a sequence of compact cylinders approaching certain closed horocycles. Let $n\in {\mathbb {N}}$ be an integer and let $\Gamma _n z\in \Gamma _n\backslash {\mathbb {H}}$ be a point close to a cusp $\mathfrak {c}\in \Omega _n$. For any $0\le j\le n-1$, the analysis in Sect. 6 gives exact information about the height of the companion point $\Gamma _n u_{j/n}z$ with respect to the cusp $u_{j/n}\mathfrak {c}$. While this is sufficient for Theorem 1.7 (cusp excursions), to realize the limiting measure $\nu _{m,Y}$ in Theorem 1.8 one needs more refined information about the spacing of these companion points along the closed horocycles they lie on. For this, we further analyze the left regular $u_{1/n}$-action on points near certain type cusps which we now define.

We say $\mathfrak {c}\in \Omega _n$ is of *simple type* if $\mathfrak {c}$ can be represented by a primitive rational number *m*/*q* satisfying that $\gcd (n^2,q)\mid n$, and we denote by $\Omega _n^{\mathrm{sim}}\subset \Omega _n$ the set of simple type cusps. (This notion of simple type cusps is closely related to the condition $n\in {\mathbb {N}}_q$ in Theorem 1.2. In fact, let *p*/*q* be a primitive rational number then the condition $n\in {\mathbb {N}}_q$ is equivalent to that the cusp $\mathfrak {c}\in \Omega _n$ represented by *p*/*q* is of simple type.) If $m'/q'$ is another representative for $\mathfrak {c}$, that is, $m'/q'$ is primitive and $m'/q'=\gamma (m/q)$ for some $\gamma \in \Gamma _n$, then using the definition of $\Gamma _n$, it is easy to check that $\gcd (n^2, q)=\gcd (n^2, q')$. Hence the simple type cusps are well-defined.

As mentioned in Sect. 3.3 the condition $\gcd (n^2,q)\mid q$ implies the further decomposition $q=kl$ with $l=\gcd (n,q)\mid n$ and $k=q/l$ satisfying $\gcd (k,n)=1$. We can thus reparameterize a simple type $\mathfrak {c}$ by *m*/(*kl*) with $\gcd (m,kl)=\gcd (k,n)=1$ and $l\mid n$. The main new ingredient of our proof to Theorem 1.8 is the following decomposition of the sample points which generalizes (3.19).

#### Proposition 7.7

Fix $n\in {\mathbb {N}}$, $z=x+iy\in {\mathbb {H}}$ and $\mathfrak {c}\in \Omega _n^{\mathrm{sim}}$. Then

$$\begin{aligned} {\mathcal {R}}_n(x,y)=\bigcup _{d\mid n}{\mathcal {R}}_{n/d}^{\mathrm{pr}}(x'_{\mathfrak {c}, d}, d^2y'/\omega _{\mathfrak {c}}), \end{aligned}$$

where $z'=x'+iy'\in {\mathbb {H}}$ is such that $\Gamma _n z=\Gamma _n \tau _{\mathfrak {c}}z'$, and $x'_{d,\mathfrak {c}}\in {\mathbb {R}}/{\mathbb {Z}}$ depends only on $x'$, $\mathfrak {c}$ and *d*.

We first prove a simple lemma computing the width of elements in the orbits $\langle u_{1/n}\rangle \mathfrak {c}$ when $\mathfrak {c}\in \Omega _n^{\mathrm{sim}}$ is of simple type.

#### Lemma 7.8

Fix $n\in {\mathbb {N}}$ and $\mathfrak {c}\in \Omega _n^{\mathrm{sim}}$ a simple type cusp. Then for any $0\le j\le n-1$ we have

$$\begin{aligned} \omega _{u_{j/n}\mathfrak {c}}=\gcd \left( m\tfrac{n}{l}+jk,n\right) ^2, \end{aligned}$$

where *m*/(*kl*) is a representative for $\mathfrak {c}$ with $\gcd (m,kl)=\gcd (k,n)=1$ and $l\mid n$.

#### Proof

For any $$0\le j\le n-1$$,

$$\begin{aligned} u_{j/n}\mathfrak {c}=\frac{m}{kl}+\frac{j}{n}=\frac{m\tfrac{n}{l}+jk}{kn}=:\frac{p_j}{q_j} \end{aligned}$$

with $\gcd (p_j,q_j)=1$. Let $d_{j}:=\gcd (m\tfrac{n}{l}+jk,kn)$ such that $q_j=kn/d_{j}$. Since $\gcd (mn,k)=1$, we have $\gcd (m\tfrac{n}{l}+jk,k)=\gcd (m\tfrac{n}{l},k)=1$. Hence $d_{j}=\gcd (m\tfrac{n}{l}+jk,n)\mid n$. Now by Lemma 7.3 and the assumption that $\gcd (k,n)=1$ we have

$$\begin{aligned} \omega _{u_{j/n}\mathfrak {c}}=\frac{n^2}{\gcd (n,kn/d_{j})^2}=d_{j}^2=\gcd (m\tfrac{n}{l}+jk,n)^2. \end{aligned}$$

$$\square $$

We can now combine ideas from Sects. 3.3 and 6 to give the

#### Proof of Proposition 7.7

Assume $\mathfrak {c}=m/(kl)$ with $\gcd (m,kl)=\gcd (k,n)=1$ and $l\mid n$. Up to changing the representatives for $\mathfrak {c}$, we may assume $mkl\ne 0$. Let $$\tau _{\mathfrak {c}}=\left( {\begin{matrix} m &{} a\\ kl &{} b\end{matrix}}\right) \in \Gamma _1$$, and for each $1\le j\le n-1$ let $$\tau _{u_{j/n}\mathfrak {c}}=\left( {\begin{matrix} p_j &{} v_j\\ q_j &{} w_j\end{matrix}}\right) \in \Gamma _1$$, where $p_j,q_j$ are as in the proof of Lemma 7.8, $a, b, v_j, w_j$ are some integers such that $\tau _{\mathfrak {c}}, \tau _{u_{j/n}\mathfrak {c}}\in \Gamma _1$, that is,

7.20 $$\begin{aligned} mb-kla=1\quad \text {and}\quad \left( m\tfrac{n}{l}+jk\right) w_j-knv_j=d_{j} \end{aligned}$$

with $d_j=\gcd (m\tfrac{n}{l}+jk,n)$ as in the proof of Lemma 7.8. By direct computation and using Lemmas 6.3 and  7.8 (and the relation $\omega _{\mathfrak {c}}=d_0^2=n^2/l^2$) we have

$$\begin{aligned} \tau _{u_{j/n}\mathfrak {c}}^{-1}u_{j/n}\tau _{\mathfrak {c}}=\begin{pmatrix} d_{j}l/n &{} w_ja+b\left( \tfrac{jw_j}{n}-v_j\right) \\ 0 &{} n/(d_{j}l)\end{pmatrix}. \end{aligned}$$

Using the relations in (7.20) the top right entry becomes

$$\begin{aligned} w_ja+b\left( \frac{jw_j}{n}-v_j\right)&=w_ja+\frac{1+kla}{m}\left( \frac{jw_j}{n}-v_j\right) \\&=\frac{a(w_jmn+jw_jkl-klv_jn)+jw_j-v_jn}{mn}\\&=\frac{ad_{j}l}{mn}+\frac{1}{mn}\left( \frac{d_{j}l-w_jmn}{kl}\right) =\frac{bd_{j}}{nk}-\frac{w_j}{kl}. \end{aligned}$$

(Here we used the assumption that $mkl\ne 0$.) Hence we have for any $0\le j\le n-1$

7.21 $$\begin{aligned} \Gamma _n u_{j/n}z&=\Gamma _n u_{j/n}\tau _{\mathfrak {c}}(x'+iy')=\Gamma _n\tau _{u_{j/n}\mathfrak {c}}\tau _{u_{j/n}\mathfrak {c}}^{-1}u_{j/n}\tau _{\mathfrak {c}}(x'+iy')\\&=\Gamma _n\tau _{u_{j/n}\mathfrak {c}}\left( \tfrac{d_{j}^2l^2}{n^2}x'+\tfrac{d^2_{j}lb}{n^2k}-\tfrac{d_{j}w_j}{kn}+i\tfrac{d_{j}^2l^2}{n^2}y'\right) .\nonumber \end{aligned}$$

Here for the first equality we used the assumption that $\Gamma _n z=\Gamma _n \tau _{\mathfrak {c}} z'$ and the fact that $u_{j/n}$ normalizes $\Gamma _n$. Now as in the proof of Proposition 3.7 for any $d\mid n$, we define

$$\begin{aligned} D_d:=\{0\le j\le n-1: d_{j}=d\} \end{aligned}$$

so that

7.22 $$\begin{aligned} {\mathcal {R}}_n(x,y)=\bigcup _{d\mid n}\left\{ \Gamma _1 u_{j/n}z\in {\mathcal {M}}: j\in D_d\right\} , \end{aligned}$$

and

7.23 $$\begin{aligned} \left\{ [(m\tfrac{n}{l}+jk)/d]\in ({\mathbb {Z}}/(n/d){\mathbb {Z}})^{\times }: j\in D_d\right\} =({\mathbb {Z}}/(n/d){\mathbb {Z}})^{\times }. \end{aligned}$$

Use the second relation in (7.20) to get for $j\in D_d$,

$$\begin{aligned} w_j\left( (m\tfrac{n}{l}+jk)/d\right) \equiv 1\ (\mathrm {mod}\ k\tfrac{n}{d}). \end{aligned}$$

Solving the above congruence equation as in the proof of Lemma 3.6 we get

$$\begin{aligned} w_j\equiv dl{\overline{mn}}\tfrac{n}{d}e+\left( \left( m\tfrac{n}{l}+jk\right) /d\right) ^*kf \ (\mathrm {mod}\ k\tfrac{n}{d}), \end{aligned}$$

where for any integer *t*, ${\overline{t}}$ denotes the multiplicative inverse modulo *k*, $t^*$ denotes the multiplicative inverse modulo *n*/*d*, and $e=e_d, f=f_d\in {\mathbb {Z}}$ are two fixed integers such that $e\tfrac{n}{d}+fk=1$. Plugging this relation into (7.21) and using the relation $\omega _{\mathfrak {c}}=n^2/l^2$ we get for any $d\mid n$ and for any $j\in D_d$,

$$\begin{aligned} \Gamma _n u_{j/n}z= \Gamma _n\tau _{u_{j/n}\mathfrak {c}}\left( x'_{\mathfrak {c},d}-\tfrac{\left( \left( m\tfrac{n}{l}+jk\right) /d\right) ^* f}{n/d}+i\tfrac{d^2y'}{\omega _{\mathfrak {c}}}\right) , \end{aligned}$$

where $x'_{\mathfrak {c},d}:=\tfrac{d^2l^2}{n^2}x'+\tfrac{d^2lb}{n^2k}-\tfrac{dl{\overline{mn}}e}{k} \ (\mathrm {mod}\ {\mathbb {Z}})\in {\mathbb {R}}/{\mathbb {Z}}$. Since $\tau _{u_{j/n}\mathfrak {c}}\in \Gamma _1$ we have

$$\begin{aligned} \left\{ \Gamma _1 u_{j/n}z\in {\mathcal {M}}: j\in D_d\right\} {=}\left\{ \Gamma _1\left( x'_{\mathfrak {c},d}{-}\tfrac{\left( \left( m\tfrac{n}{l}+jk\right) /d\right) ^* f}{n/d}+i\tfrac{d^2y'}{\omega _{\mathfrak {c}}}\right) \in {\mathcal {M}}: j\in D_d\right\} . \end{aligned}$$

Thus in view of (7.22) and the above relation it suffices to show

$$\begin{aligned} \left\{ -[\left( (m\tfrac{n}{l}+jk)/d\right) ^*f]\in ({\mathbb {Z}}/(n/d){\mathbb {Z}})^{\times }: j\in D_d\right\} =({\mathbb {Z}}/(n/d){\mathbb {Z}})^{\times }. \end{aligned}$$

But this follows from (7.23) and the fact that $\gcd (f, \tfrac{n}{d})=1$ (since $\gcd (f, \tfrac{n}{d})=\gcd (fk,\tfrac{n}{d})=\gcd (1-e\tfrac{n}{d}, \tfrac{n}{d})=1$), and we have thus finished the proof. $\square$

We will also need the following lemma estimating the number of cusps in $\Omega _n^{\mathrm{sim}}$ satisfying certain restrictions on the width.

#### Lemma 7.9

Let $m\in {\mathbb {N}}$ be a fixed integer and let $n=m\ell \ge 3$ for some prime number $\ell$ not dividing *m*. Then we have

$$\begin{aligned} \#\left\{ \mathfrak {c}\in \Omega ^{\mathrm{sim}}_{n}: \omega _{\mathfrak {c}}\ge m^2\right\} \ge \frac{\varphi (m)(\ell -1)^2}{2}. \end{aligned}$$

#### Proof

Recall from the proof of Lemma 7.5 that $\Omega _n$ is in bijection with the disjoint union $\bigsqcup _{d\mid n^2} J_n\backslash Z_d$. On the other hand, by definition of the simple type cusps, $\Omega _n^{\mathrm{sim}}$ corresponds to the subset $\sqcup _{d\mid n} J_n\backslash Z_d$. Moreover, let $\mathfrak {c}=m/l\in \Omega _n^{\mathrm{sim}}$ with $\gcd (m,l)=1$ be a simple type cusp corresponding to an element in $J_n\backslash Z_d$ for some $d\mid n$, that is, $d=\gcd (n^2,l)$. Since $d\mid n$, this implies that $d=\gcd (n^2,l)=\gcd (n,l)$. Hence by Lemma 7.3, $\omega _\mathfrak {c}= n^2/d^2$. Therefore for each $d\mid n$

$$\begin{aligned} \#\{\mathfrak {c}\in \Omega _n^{\mathrm{sim}} : \omega _\mathfrak {c}=n^2/d^2 \} = |J_n\backslash Z_d|\ =\ \frac{\varphi (n^2/d)\varphi (d)\gcd (n^2/d,d)}{2n} = \frac{\varphi (n)\varphi (d)}{2}, \end{aligned}$$

$\square$ where for the last equality we used the identities $\gcd (n^2/d,d)=d$ (since $d\mid n$) and

$$\begin{aligned} \varphi \left( \tfrac{n^2}{d}\right) =\frac{n^2}{d}\prod _{\begin{array}{c} p\mid (n^2/d)\\ prime \end{array}}(1-p^{-1})=\frac{n}{d}\times n\prod _{\begin{array}{c} p\mid n\\ prime \end{array}}(1-p^{-1})=\frac{n\varphi (n)}{d}, \end{aligned}$$

where for the second equality we used the fact that $n^2/d$ and *n* share the same set of prime divisors. Hence for $n=m\ell$ we have

$$\begin{aligned} \#\left\{ \mathfrak {c}\in \Omega ^{\mathrm{sim}}_{n}: \omega _{\mathfrak {c}}\ge m^2\right\}&=\frac{\varphi (n)}{2}\sum _{\begin{array}{c} d\mid n\\ n^2/d^2\ge m^2 \end{array}}\varphi (d)\ge \frac{\varphi (n)\varphi (\ell )}{2}=\frac{\varphi (m)(\ell -1)^2}{2}. \end{aligned}$$

$$\square $$

#### Lemma 7.10

Let $m\in {\mathbb {N}}$ and $Y>0$ satisfy that $m^2Y>1$. Let

$$\begin{aligned} {\mathbb {P}}_m=\{n=m\ell \in {\mathbb {N}}: \ell \,\text {is a prime number and}\, \ell \not \mid m\} \end{aligned}$$

be as in (1.8). Then there exist sequences of positive numbers $\{Y_n\}_{n\in {\mathbb {P}}_m}$ and $\{Y_n'\}_{n\in {\mathbb {P}}_m}$ satisfying that

1. $Y_n'>Y>Y_n>m^{-2}$ for any $n\in {\mathbb {P}}_m$ and $$\lim \nolimits _{\begin{array}{c} n\in {\mathbb {P}}_m\\ n\rightarrow \infty \end{array}}Y_n=\lim \nolimits _{\begin{array}{c} n\in {\mathbb {P}}_m\\ n\rightarrow \infty \end{array}}Y'_n=Y$$;
2. $$\sum _{n\in {\mathbb {P}}_m}\frac{1}{n}\left( \frac{1}{Y_n}-\frac{1}{Y_n'}\right) =\infty $$.

#### Proof

For each $n=m\ell \in {\mathbb {P}}_m$, take $Y'_n:=(1-(2t_n)^{-1})^{-1}Y$ and $Y_n:=(1+(2t_n)^{-1})^{-1}Y$ with

$$\begin{aligned} t_n=\max \{(m^2Y-1)^{-1}, \log \log \ell \}. \end{aligned}$$

We note that the first condition is guaranteed by the facts that $t_n\ge (m^2Y-1)^{-1}$ and that $$\lim \nolimits _{\begin{array}{c} n\in {\mathbb {P}}_m\\ n\rightarrow \infty \end{array}}t_n=\infty $$. For the second condition, we note that by the definitions of $Y_n$ and $Y_n'$, $\frac{1}{Y_n}-\frac{1}{Y_n'}=\frac{1}{Yt_n}$. Moreover, using the fact that there are only finitely many prime numbers dividing *m* we get

$$\begin{aligned} \sum _{n\in {\mathbb {P}}_m}\frac{1}{n}\left( \frac{1}{Y_n}-\frac{1}{Y_n'}\right) \asymp _{m,Y}\sum _{\begin{array}{c} \ell \in {\mathbb {P}}_1\\ \ell \not \mid m \end{array}}\frac{1}{\ell \log \log \ell }=\sum _{\ell \in {\mathbb {P}}_1}\frac{1}{\ell \log \log \ell }+O_m(1)=\infty , \end{aligned}$$

where the divergence of the rightmost series follows from the estimate $\ell _j\asymp j\log j$ which is an easy consequence of the prime number theorem. Here $\ell _j\in {\mathbb {P}}_1$ denotes the *j*-th prime number. $\square$

We now give the

#### Proof of Theorem 1.8

Fix throughout the proof $m\in {\mathbb {N}}$ and $Y>0$ with $m^2Y>1$ and let ${\mathbb {P}}_m$ be as above. Let $\{Y_n\}_{n\in {\mathbb {P}}_{m}}$ and $\{Y_n'\}_{n\in {\mathbb {P}}_m}$ be two sequences satisfy the conditions in Lemma 7.10. For any $n\in {\mathbb {P}}_m$, let $\Psi _n\in L^2(\Gamma _n\backslash {\mathbb {H}})$ such that $\Psi _n$ is the indicator function of the union

$$\begin{aligned} \bigcup _{\begin{array}{c} \mathfrak {c}\in \Omega _{n}^{\mathrm{sim}}\\ \omega _{\mathfrak {c}}\ge m^2 \end{array}}{\mathcal {C}}_{\omega _{\mathfrak {c}}Y_n, \omega _{\mathfrak {c}}Y'_n}^{n,\mathfrak {c}}\subset \Gamma _{n}\backslash {\mathbb {H}}. \end{aligned}$$

Since $Y_n>m^{-2}$ for any $n\in {\mathbb {P}}_m$, $\omega _{\mathfrak {c}}Y_n>1$ for any $\mathfrak {c}\in \Omega _{n}^{\mathrm{sim}}$ with $\omega _{\mathfrak {c}}\ge m^2$. Hence similar as in the proof of Theorem 1.7, by Lemmas 6.1 and  6.2 the above union is disjoint and $\Psi _n$ is the indicator function of a Borel set with boundary of measure zero. By the disjointness and the volume formula (6.2) we have for any $n\in {\mathbb {P}}_m$

$$\begin{aligned} \mu _{\Gamma _{n}}\left( \Psi _n\right) = \frac{3}{\pi }\frac{\#\left\{ \mathfrak {c}\in \Omega ^{\mathrm{sim}}_{n}: \omega _{\mathfrak {c}}\ge m^2\right\} }{[\Gamma _1: \Gamma _{n}]}\left( \frac{1}{Y_n}-\frac{1}{Y_n'}\right) . \end{aligned}$$

Note that for $n=m\ell \in {\mathbb {P}}_m$, by Lemma 7.2, $[\Gamma _1: \Gamma _{n}]\asymp _m \ell ^3$. Hence by Lemma 7.9 and the above relation we get for any $n=m\ell \in {\mathbb {P}}_m$

7.24 $$\begin{aligned} \mu _{\Gamma _{n}}\left( \Psi _n\right) \gg _{m,Y} \frac{1}{\ell }\left( \frac{1}{Y_n}-\frac{1}{Y_n'}\right) \asymp _m\frac{1}{n}\left( \frac{1}{Y_n}-\frac{1}{Y_n'}\right) . \end{aligned}$$

Similar as in the proof of Theorem 1.7 for any $n\in {\mathbb {P}}_m$ and $0<y<1$ we define

$$\begin{aligned} I_n(y):=\left\{ x\in {\mathbb {R}}/{\mathbb {Z}}:\Psi _n(x+iy)=1\right\} . \end{aligned}$$

We first show that there exists a sequence $\{y_n\}_{n\in {\mathbb {P}}_m}$ satisfying that $0<y_n<c_n$ for all $n\in {\mathbb {P}}_m$ and that the limsup set $$\mathop {{\overline{\lim }}}_{\begin{array}{c} n\in {\mathbb {P}}_m\\ n\rightarrow \infty \end{array}}I_n(y_{n})\subset {\mathbb {R}}/{\mathbb {Z}}$$ is of full measure. As in the proof of Theorem 1.7, we can use Remark 2.17, together with Remark 6.3 and Lemma 6.1, to construct a sequence $\{y_{n}\}_{n\in {\mathbb {P}}_m}$ successively satisfying for any $n\in {\mathbb {P}}_m$, $0<y_n<c_n$ and that

7.25 $$\begin{aligned} \left| \frac{1}{\left| I\right| }\int _{I}\Psi _n(x+iy_{n})dx-\mu _{\Gamma _{n}}(\Psi _n)\right| \le \frac{\mu _{\Gamma _{n}}(\Psi _n) }{2n^2} \end{aligned}$$

for all subsets $I\subset {\mathbb {R}}/{\mathbb {Z}}$ taken from the finite set $\left\{ (0,1)\right\} \bigcup \left\{ I_{l}(y_l): l\in {\mathbb {P}}_m,\ l<n\right\}$. Again as before one can show that condition (7.25) implies that the sequence $\{I_n(y_{n})\}_{n\in {\mathbb {P}}_m}\subset {\mathbb {R}}/{\mathbb {Z}}$ satisfies the quasi-independence condition (2.20) (with the subset ${\mathbb {S}}={\mathbb {P}}_m$ and exponent $\eta =2$). Moreover, using the estimate (7.24) and our assumptions on $\{Y_n\}_{n\in {\mathbb {P}}_m}$ and $\{Y_n'\}_{n\in {\mathbb {P}}_m}$) we have

$$\begin{aligned} \sum _{n\in {\mathbb {P}}_m}\left| I_n(y_{n})\right| \asymp \sum _{n\in {\mathbb {P}}_m}\mu _{\Gamma _{n}}\left( \Psi _n\right) \gg _{m,Y} \sum _{n\in {\mathbb {P}}_m}\frac{1}{n}\left( \frac{1}{Y_n}-\frac{1}{Y_n'}\right) =\infty . \end{aligned}$$

Hence by Corollary 2.6, $$\mathop {{\overline{\lim }}}_{\begin{array}{c} n\in {\mathbb {P}}_m\\ n\rightarrow \infty \end{array}}I_n(y_{n})\subset {\mathbb {R}}/{\mathbb {Z}}$$ is of full Lebesgue measure.

Now take $$x\in \mathop {{\overline{\lim }}}_{\begin{array}{c} n\in {\mathbb {P}}_m\\ n\rightarrow \infty \end{array}}I_n(y_{n})$$, then there exists an unbounded subsequence ${\mathcal {N}}_x\subset {\mathbb {P}}_m$ such that $x\in I_{n}(y_{n})$ for all $n\in {\mathcal {N}}_x$. It thus suffices to show that for any $\Psi \in C_c^{\infty }({\mathcal {M}})$,

$$\begin{aligned} \lim \limits _{\begin{array}{c} n\in {\mathcal {N}}_x\\ n\rightarrow \infty \end{array}}\delta _{n,x,y_{n}}(\Psi )=\nu _{m,Y}(\Psi ) \end{aligned}$$

with $\nu _{m,Y}$ defined as in (1.9). For any $n\in {\mathcal {N}}_x\subset {\mathbb {P}}_m$, since $x\in I_n(y_n)$ by definition we have $\Gamma _{n}(x+iy_n)\in {\mathcal {C}}_{\omega _{\mathfrak {c}}Y_n, \omega _{\mathfrak {c}}Y'_n}^{n,\mathfrak {c}}$ for some $\mathfrak {c}\in \Omega _{n}^{\mathrm{sim}}$ of simple type, that is, there exist some $\mathfrak {c}\in \Omega _{n}^{\mathrm{sim}}$ and $z_n'=x'_n+i\omega _{\mathfrak {c}}y'_n\in {\mathbb {H}}$ satisfying that $\Gamma _{n}(x+iy_{n})=\Gamma _{n}\tau _{\mathfrak {c}} z'_n$ with $Y_n< y'_n<Y_n'$. Then by Proposition 7.7, we have

$$\begin{aligned} {\mathcal {R}}_{n}(x,y_{n})=\bigcup _{d\mid n}{\mathcal {R}}_{n/d}^{\mathrm{pr}}(x'_{n,\mathfrak {c}, d}, d^2y'_n) \end{aligned}$$

for some $x'_{n,\mathfrak {c},d}\in {\mathbb {R}}/{\mathbb {Z}}$. This implies that for any $n\in {\mathcal {N}}_x$

$$\begin{aligned} \delta _{n,x, y_{n}}(\Psi )=\frac{1}{n}\sum _{d\mid n}\varphi \left( \tfrac{n}{d}\right) \delta ^{\mathrm{pr}}_{n/d, x'_{n,\mathfrak {c},d}, d^2y'_n}(\Psi ). \end{aligned}$$

Since $y'_n, Y\in (Y_n , Y_n')$, $\max \{y'_n/Y, Y/y'_n\}\le Y_n'/Y_n$. Thus by the intermediate value theorem we can estimate for $n\in {\mathcal {N}}_x$

$$\begin{aligned} \delta _{n,x, y_{n}}(\Psi )&=\frac{1}{n}\sum _{d\mid n}\varphi \left( \tfrac{n}{d}\right) \left( \delta ^{\mathrm{pr}}_{n/d, x'_{n,\mathfrak {c},d}, d^2Y}(\Psi )+O\left( {\mathcal {S}}^{\Gamma _1}_{\infty ,1}(\Psi )\log \left( Y_n'/Y_n\right) \right) \right) \\&=\frac{1}{n}\sum _{d\mid n}\varphi \left( \tfrac{n}{d}\right) \delta ^{\mathrm{pr}}_{n/d, x'_{n,\mathfrak {c},d}, d^2Y}(\Psi )+O_{\Psi }\left( \log \left( Y_n'/Y_n\right) \right) , \end{aligned}$$

where for the second estimate we used the identity $\sum _{d\mid n}\varphi (n/d)=n$. Thus for $n=m\ell \in {\mathcal {N}}_x$ sufficiently large such that $\Psi$ vanishes on the cusp neighborhood ${\mathcal {C}}_{\ell ^2Y}$ we have

$$\begin{aligned} \delta _{n,x, y_{n}}(\Psi )&=\frac{1}{m\ell }\sum _{d\mid m}\varphi \left( \tfrac{m\ell }{d}\right) \delta ^{\mathrm{pr}}_{m\ell /d, x'_{n,\mathfrak {c},d}, d^2Y}(\Psi )+O_{\Psi }\left( \log \left( Y_n'/Y_n\right) \right) \\&=\frac{\ell -1}{m\ell }\sum _{d\mid m}\varphi \left( \tfrac{m}{d}\right) \left( \mu _{d^2Y}(\Psi )+O_{\Psi , m,Y,\epsilon }\left( \ell ^{-1+\epsilon }\right) \right) +O_{\Psi }\left( \log \left( Y_n'/Y_n\right) \right) \\&=\frac{\ell -1}{\ell }\nu _{m,Y}(\Psi )+O_{\Psi , m,Y,\epsilon }\left( \ell ^{-1+\epsilon }+\log \left( Y_n'/Y_n\right) \right) , \end{aligned}$$

where for the second equality we used the facts that $\ell$ is a prime number and $\gcd (m,\ell )=1$ and applied the effective estimate (3.13) to each of the term $\delta ^{\mathrm{pr}}_{m\ell /d, x'_{n,\mathfrak {c},d}, d^2Y}(\Psi )$. We now conclude by taking $n\rightarrow \infty$ along the subsequence ${\mathcal {N}}_x$ and noting that $$\lim \nolimits _{\begin{array}{c} n\in {\mathcal {N}}_x\\ n\rightarrow \infty \end{array}}\log \left( Y_n'/Y_n\right) =0$$ (since $$\lim \nolimits _{\begin{array}{c} n\in {\mathbb {P}}_m\\ n\rightarrow \infty \end{array}}Y_n'/Y_n=1$$ which follows from the assumption $$\lim \nolimits _{\begin{array}{c} n\in {\mathbb {P}}_m\\ n\rightarrow \infty \end{array}}Y_n=\lim \nolimits _{\begin{array}{c} n\in {\mathbb {P}}_m\\ n\rightarrow \infty \end{array}}Y'_n=Y$$). $\square$

#### Remark 7.26

It is clear that we can take a sequence $\{y_n\}_{n\in {\mathbb {N}}}$ decaying sufficiently fast such that the conditions (7.9) and (7.25) (for any finitely many pairs (*m*, *Y*) with $m^2Y>1$) are all satisfied and hence (noting that the intersection of finitely many full measure sets is still of full measure) for such a sequence the conclusions of Theorems 1.7 and  1.8 (for any finitely many pairs (*m*, *Y*) with $m^2Y>1$) hold simultaneously.
